# Metal‐Mediated Oligomerization Reactions of the Cyaphide Anion

**DOI:** 10.1002/anie.202218047

**Published:** 2023-02-01

**Authors:** Eric S. Yang, Daniel W. N. Wilson, Jose M. Goicoechea

**Affiliations:** ^1^ Department of Chemistry University of Oxford Chemistry Research Laboratory 12 Mansfield Road Oxford OX1 3TA UK; ^2^ Department of Chemistry Indiana University—Bloomington 800 E. Kirkwood Ave. Bloomington IN-47405-7102 USA

**Keywords:** Cyaphide, Ligands, Metal Complexes, Oligomerization, Phosphorus

## Abstract

The cyaphide anion, CP^−^, is shown to undergo three distinct oligomerization reactions in the coordination sphere of metals. Reductive coupling of Au(IDipp)(CP) (IDipp=1,3‐bis(2,6‐diisopropylphenyl)imidazol‐2‐ylidene) by Sm(Cp*)_2_(OEt_2_) (Cp*=1,2,3,4,5‐pentamethylcyclopentadienyl), was found to afford a tetra‐metallic complex containing a 2,3‐diphosphabutadiene‐1,1,4,4‐tetraide fragment. By contrast, non‐reductive dimerization of Ni(SIDipp)(Cp)(CP) (SIDipp=1,3‐bis(2,6‐diisopropylphenyl)‐imidazolidin‐2‐ylidene; Cp=cyclopentadienyl), gives rise to an asymmetric bimetallic complex containing a 1,3‐diphosphacyclobutadiene‐2,4‐diide moiety. Spontaneous trimerization of Sc(Cp*)_2_(CP) results in the formation of a trimetallic complex containing a 1,3,5‐triphosphabenzene‐2,4,6‐triide fragment. These transformations show that while cyaphido transition metal complexes can be readily accessed using metathesis reactions, many such species are unstable to further oligomerization processes.

## Graphical abstract

We describe three distinct oligomerization reactions of metal complexes bonded to the cyaphide ligand (CP^−^). Reductive coupling of a gold cyaphido complex was found to afford a species containing a C_2_P_2_^4−^ dimer. By contrast, sterically unprotected metal complexes with more polar M−CP (M=Ni, Sc) bonds were found to spontaneously oligomerize giving rise to species with bridging C_2_P_2_^2−^ and C_3_P_3_^3−^ ligands.

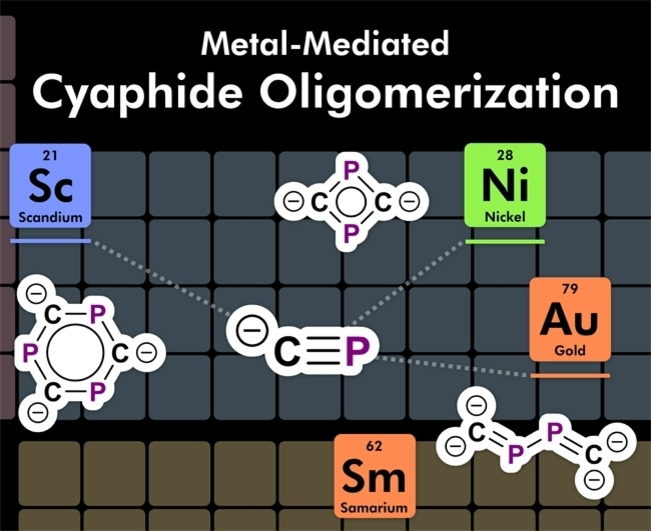

## Introduction

**Figure 1:**
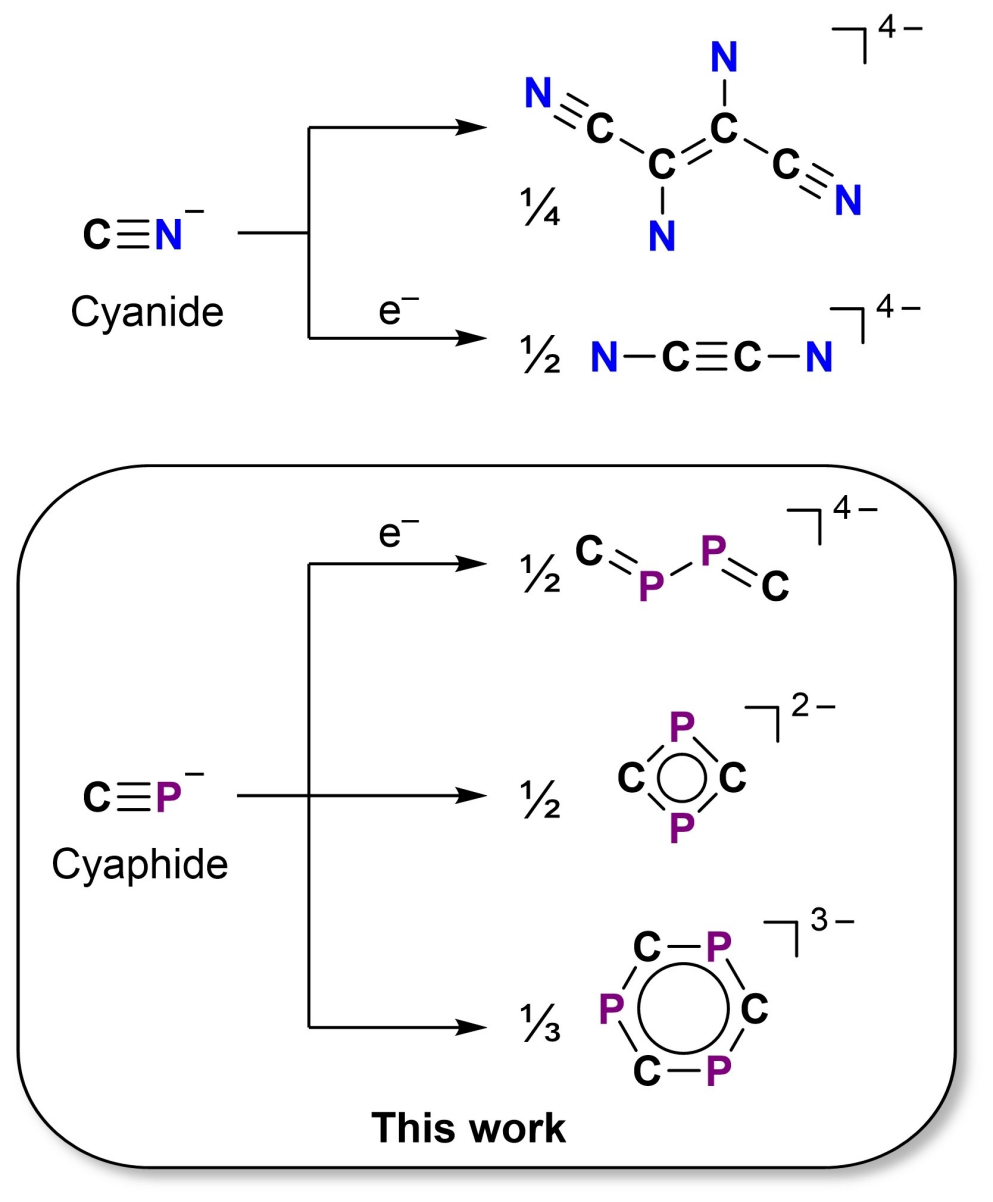
Top: Oligomerization reactions of cyanides; Bottom: Oligomerization reactions of the cyaphide ion reported in this work.

The cyanide ion is an important component of technologically relevant molecules and materials, such as Prussian Blue Analogues.[^1^, ^2^, ^3^, ^4^, ^5^, ^6^] Cyanide‐containing organic molecules, or nitriles, are also highly important in materials chemistry as monomer precursors to triazines, which are privileged scaffolds in reticular chemistry for the construction of framework materials, e.g. Covalent Organic Frameworks (COFs) and Metal‐Organic Frameworks (MOFs).[^7^, ^8^, ^9^, ^10^, ^11^] While the oligomerization of nitriles is achievable by several methods, including acid catalysis and at elevated temperatures,[^12^, ^13^, ^14^] the predominant oligomerization pathway for the cyanide ion is by oxidation (to afford cyanogen).^[15]^ More uncommonly, the cyanide ion will afford a tetrameric C_4_N_4_^4−^ moiety,[^16^, ^17^] or the reduced dimer C_2_N_2_^4−^ (Figure 1).^[18]^

Phosphaalkynes are phosphorus‐containing heavy analogues of nitriles.^[19]^ As with nitriles, the oligomerization of phosphaalkynes is well studied, and can be achieved both stoichiometrically and catalytically using transition metal complexes.[^20^, ^21^, ^22^, ^23^, ^24^, ^25^, ^26^, ^27^, ^28^] The cyaphide ion, CP^−^, is the heavier analogue of cyanide, and also capable of bonding to metal centers.^[29]^ It is much rarer than its lighter congener, and relatively few examples of cyaphide‐containing molecules have been reported in the three decades since it was first discovered.[^30^, ^31^, ^32^, ^33^, ^34^] As a result, there are only a handful of reports exploring the reactivity of the cyaphide ion,[^34^, ^35^, ^36^] and its chemistry remains poorly understood in comparison to cyanides or phosphaalkynes.

We recently reported a general synthetic route to cyaphide‐containing molecules using a magnesium(II) cyaphide transfer reagent Mg(^Dipp^NacNac)(dioxane)(CP) (**A**; ^Dipp^NacNac=CH{C(CH_3_)N(Dipp)}_2_; Dipp=2,6‐diisopropylphenyl), enabling the isolation of reactive cyaphido transition metal complexes.^[37]^ In contrast to the relatively inert behavior of cyanide ligands, metal cyaphide complexes are significantly more reactive due to the weak nature of the C(2p)−P(3p) π‐bond. While this presents some challenges for the isolation of cyaphido metal complexes, it also provides an opportunity for the generation of longer chained, conjugated organophosphorus anions that may serve as linkers between metal atoms, for example in MOFs. Further, through the study of these oligomerization processes we can gain understanding of the factors that govern the stability of transition metal cyaphide complexes, which will inform the design of cyaphide‐containing materials such as analogues of Prussian Blue.

In this work, we report a pattern of reactivity of the cyaphide ion toward oligomerization to form di‐, tri‐, and tetra‐anionic cyaphide oligomers.

## Results and Discussion

**Scheme 1:**
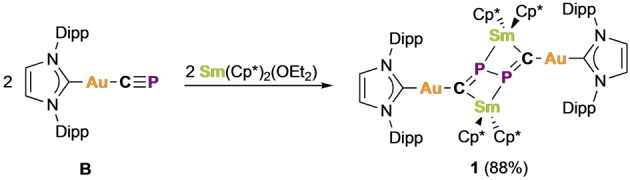
Synthesis of **1** by reductive coupling of **B**.

Many fragments isolobal to the cyaphide ion are known to oligomerize by reductive coupling, including phosphaalkynes,^[19]^ cyanides,^[18]^ alkynes,^[38]^ and carbon monoxide.^[39]^ Prompted by the phosphaalkyne‐like reactivity of the gold(I) cyaphide complex Au(IDipp)(CP) (**B**) in heterometallic complexes,^[36]^ we reasoned that it too may oligomerize on one‐electron reduction. The reaction of **B** with the samarium(II) reagent Sm(Cp*)_2_(OEt_2_)^[40]^ under slow‐diffusion conditions results in the formation of the reductively coupled product {Au(IDipp)}_2_{Sm(Cp*)_2_}_2_(μ_4_‐C_2_P_2_) (**1**) as insoluble dark yellow‐brown crystals (Scheme 1).^[41]^ Although its insolubility in highly polar organic solvents (e.g. 1,2‐difluorobenzene and dimethylsulfoxide), even upon heating, precluded characterization by nuclear magnetic resonance (NMR) spectroscopy, its structure could be confirmed by single crystal X‐ray crystallography.

**Figure 2:**
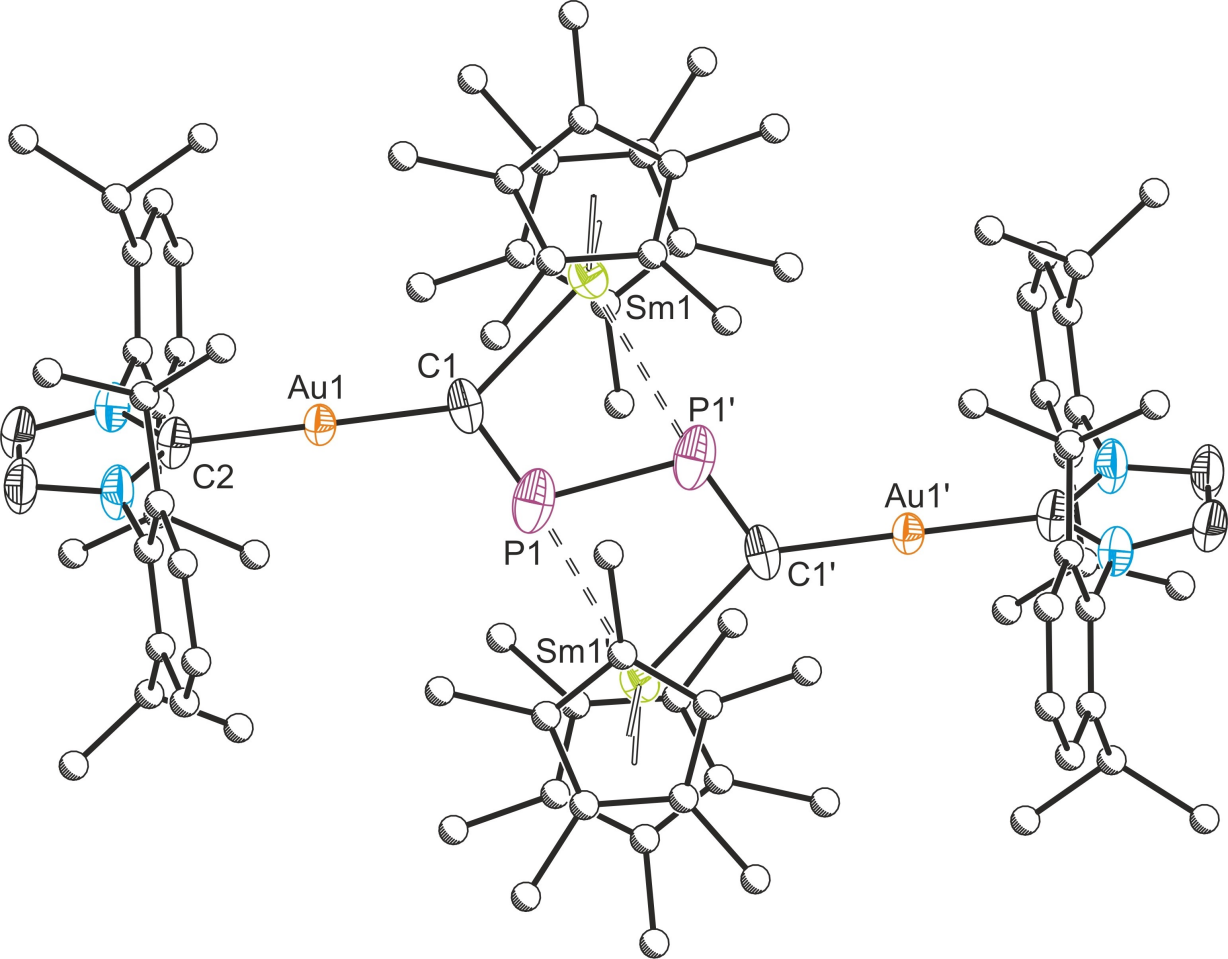
Solid‐state structure of **1**. Anisotropic displacement ellipsoids set at 50 % probability. Hydrogen atoms omitted for clarity. Selected bond lengths (Å) and angles (°): Au1−C1: 2.031(5), Au1−C2: 2.033(5), C1−P1: 1.623(6), P1−P1′: 2.306(3), Sm1−C1: 2.461(6), Sm1−P1′: 2.894(2); C1−Au1−C2: 179.3(2), Au1−C1−P1: 118.2(3), C1−P1−P1′: 110.4(2), Au1−C1−Sm1: 141.0(3), C1−Sm1−P1′: 74.24(14). Symmetry operation ′: 1−*x*, 1−*y*, −*z*. The crystal structure reveals positional disorder of **1** in the asymmetric unit (approx. 10 % occupancy for the minor component).

The solid‐state structure of **1** contains a reduced cyaphide dimer, C_2_P_2_^4−^, in which the two halves of the molecule are joined by a single bond between the phosphorus atoms (Figure 2). This is in contrast to the behavior observed for the cyanide ion which forms a C−C bond on reductive coupling.^[18]^ The P1−P1′ bond length is 2.306(3) Å, and the C1−P1 bond lengths are 1.623(6) Å. The central C_2_P_2_^4−^ moiety is bonded to the two gold centers by its carbon atoms, and to two samarium centers by both carbon and phosphorus. The Au1−C1 bond length is 2.031(5) Å, the Sm1−C1 bond length is 2.461(6) Å, and the Sm1−P1 bond length is 2.894(2) Å. The C1−P1−P1′ bond angle is 110.4(2)°, and the Au1−C1−P1 angle is 118.2(3)°, suggesting sp^2^ hybridization of the carbon atom. **1** is isostructural to a previously reported 2,3‐diphosphabutdiene‐1,4‐diide ion,^[20]^ and is also structurally similar to reported carbene‐stabilized moieties.^[42]^

At room temperature **B** does not oligomerize spontaneously despite poor steric protection of the C≡P triple bond. However, we have found that the attempted syntheses of other sterically unprotected cyaphide complexes frequently results in uncontrolled reactivity. We hypothesized that this could be due to poor electronic stabilization of the cyaphide ion, which in turn allows for oligomerization or polymerization to occur. With the aim of investigating this possibility, we targeted the synthesis of cyaphide complexes featuring a range of different transition metals.

**Scheme 2:**
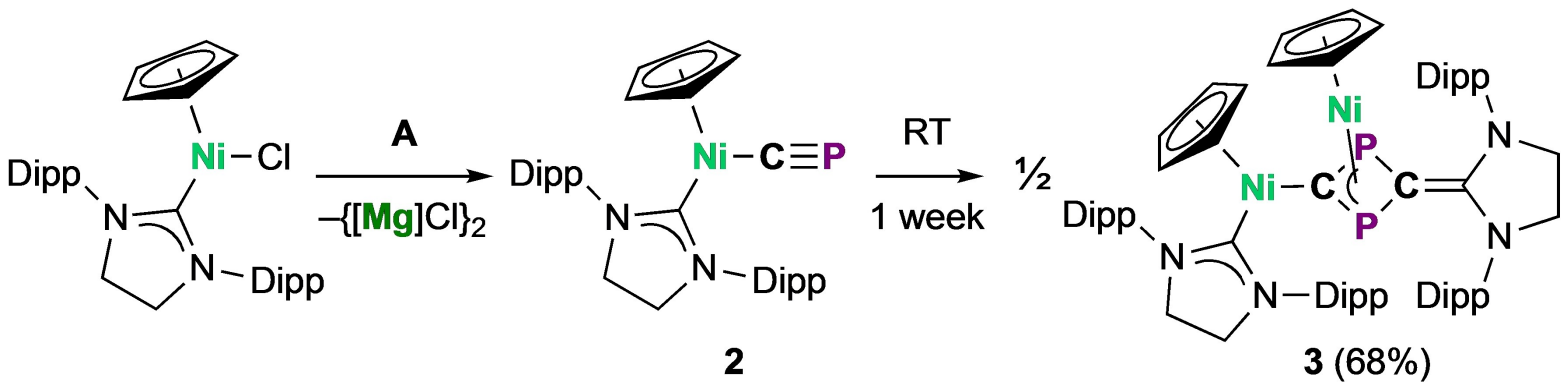
Synthesis of **2** and subsequent spontaneous dimerization to form **3**; [Mg]=Mg(^Dipp^NacNac).

The reaction of **A** with Ni(SIDipp)(Cp)Cl results in the formation of the nickel(II) cyaphide complex Ni(SIDipp)(Cp)(CP) (**2**) (Scheme 2).^[43]^ Compound **2** exhibits a singlet resonance in its ^31^P{^1^H} NMR spectrum at 181.2 ppm corresponding to the cyaphide phosphorus atom, as well as a doublet in its ^13^C{^1^H} spectrum at 224.02 ppm (^1^ *J*_C‐P_=10.4 Hz) corresponding to the cyaphide carbon atom. The C≡P stretching frequency of **2** appears at 1307 cm^−1^ in its infra‐red (IR) spectrum (cf. 1342 cm^−1^ for **B**) (Figure S4).^[37]^ To our surprise, attempts to grow X‐ray quality crystals of **2** at room temperature over 1 week (or at −35 °C over several weeks) resulted in the crystallization of an asymmetric bimetallic complex, {Ni(SIDipp)(Cp)}{Ni(Cp)}{μ_2_‐(SIDipp)C_2_P_2_} (**3**), the product of spontaneous dimerization of **2**. A closely related complex has been invoked by Wolf as an intermediate in the nickel‐mediated dimerization of *tert*‐butyl phosphaalkyne to afford a diphosphatetrahedrane.^[26]^

**Figure 3:**
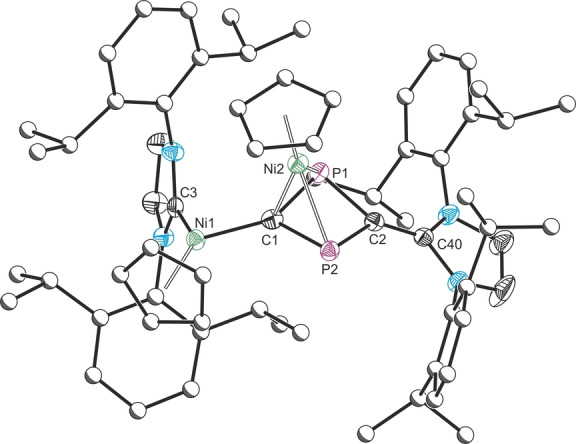
Solid‐state structure of **3**. Anisotropic displacement ellipsoids set at 50 % probability. Hydrogen atoms omitted for clarity. Selected bond lengths (Å) and angles (°): Ni1−C1: 1.892(3), C1−P1: 1.820(3), C1−P2: 1.794(3), P1−C2: 1.815(3), C2−P2: 1.824(3), Ni2−C1: 1.992(3), Ni2−P1: 2.253(1), Ni2−P2: 2.266(1), Ni2−C2: 2.861(3), P1⋅⋅⋅P2: 2.588(2), C2−C40: 1.352(4); Ni1−C1−P1: 127.2(2); Ni1−C1−P2: 141.3(2); C1−P1−C2: 85.4(2); C1−P2−C2: 85.9(2); P1−C1−P2: 91.5(2); P1−C2−P2: 90.6(2).

The ^31^P{^1^H} NMR spectrum of **3** reveals an AX spin system with doublets at 130.3 and 119.9 ppm (^2^ *J*_P‐P_=65 Hz), consistent with the presence of two inequivalent phosphorus environments (due to restricted rotation of the [Ni(SIDipp)(Cp)] moiety). The ^1^H and ^13^C{^1^H} spectra of **3** show two inequivalent Cp ligands, as well as substantial desymmetrization of the two SIDipp ligands. The solid‐state structure of **3** reveals a dimeric product containing a dianionic C_2_P_2_^2−^ ring that has also inserted between a nickel‐carbene bond (Figure 3). The four‐membered ring has C−P bond lengths between 1.794(3) and 1.824(3) Å, lying in between the predicted values for carbon‐phosphorus single (1.96 Å) and double bonds (1.69 Å).^[44]^ The ring is slightly puckered (mean deviation from plane: 0.15 Å), with P1−C−P2 bond angles of 91.5(2)° and 90.6(2)°, and C1−P−C2 bond angles of 85.4(2)° and 85.9(2)°. The C−C bond length between the cyclic dimer and the carbene is 1.352(4) Å, in good agreement with a double bond (1.34 Å). The C_2_P_2_ ring is bonded to the two nickel atoms in different manners. It coordinates to the Ni(SIDipp)(Cp) fragment with a η^1^ hapticity and a Ni1−C1 bond length of 1.892(3) Å. The Ni(Cp) fragment is instead bonded facially to the C_2_P_2_ ring with a η^3^ hapticity; the Ni2−C1 bond length is 1.992(2) Å and the Ni2−P bond lengths are 2.253(1) Å and 2.266(1) Å.

**Figure 4:**
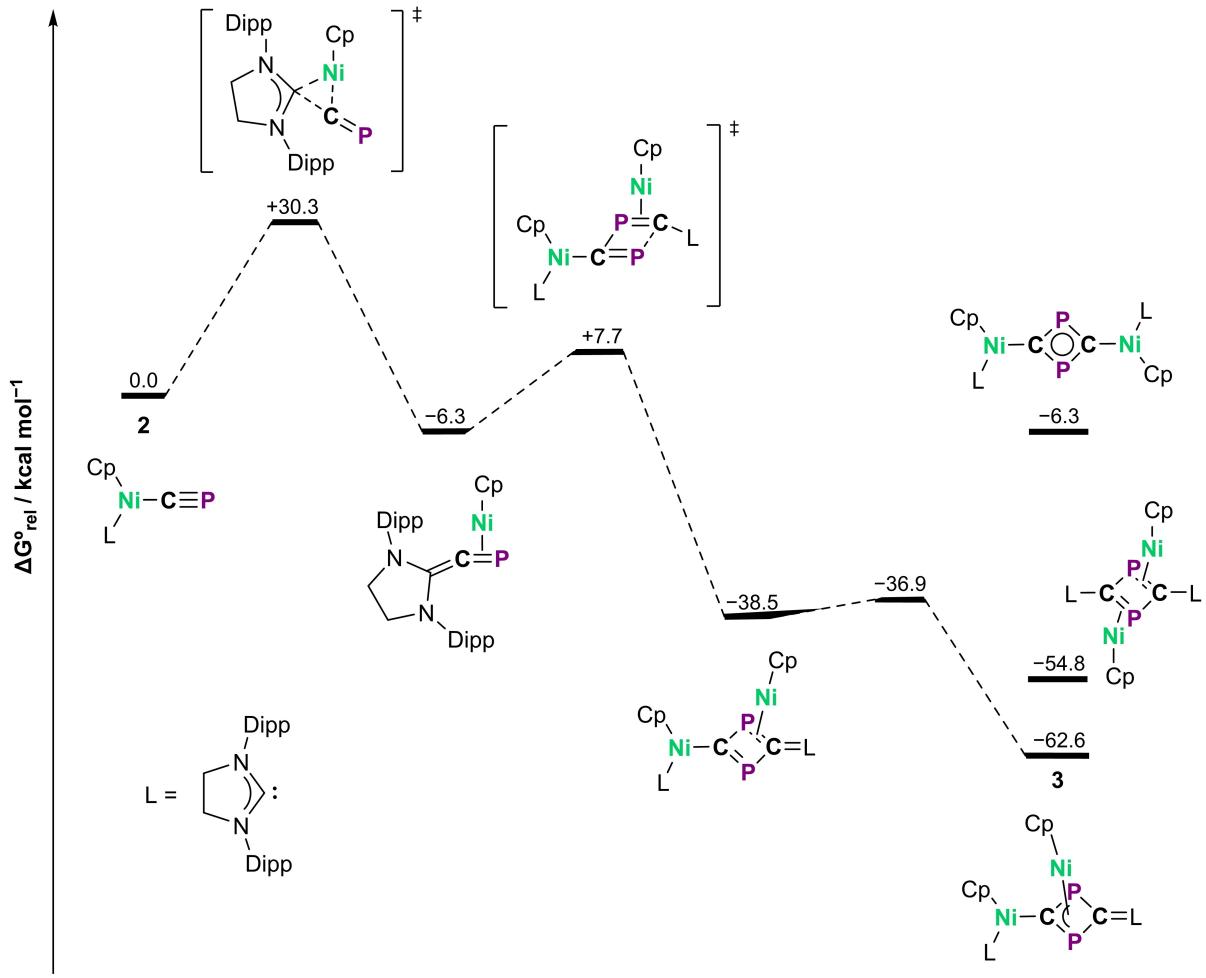
Calculated mechanism for the formation of **3** from **2** (ZORA‐ωB97X‐D3/ZORA‐def2‐TZVP). The energies of other plausible dimerization products are also shown for comparison.

The mechanism of the dimerization of **2** was investigated by density functional theory (DFT) calculations (see the Supporting Information for details). The mechanism proceeds via an initial rate‐limiting migratory insertion of the cyaphide ion into the nickel carbene bond (Figure 4), forming a η^2^‐phosphaallenide intermediate. Related migratory insertions of isocyanides on palladium(II) have previously been reported.^[45]^ This insertion step can be compared to the reverse reaction of the recently reported oxidative addition of the sp^2^‐sp C−C bond of Ar−CP (Ar=2,4,6‐trimethylphenyl) by the heavier group 10 complex LPt (L=1,2‐bis(dicyclohexylphosphino)ethane).^[34]^ The transient η^2^‐phosphaallenide reacts with another equivalent of **2** in a [2+2] cycloaddition reaction, followed by a η^2^–η^3^ hapticity change to afford **3**. Alternate plausible dimerization products with η^1^ : η^1^ and η^2^ : η^2^ hapticities were both found to be higher in energy than **3**.

**Figure 5:**
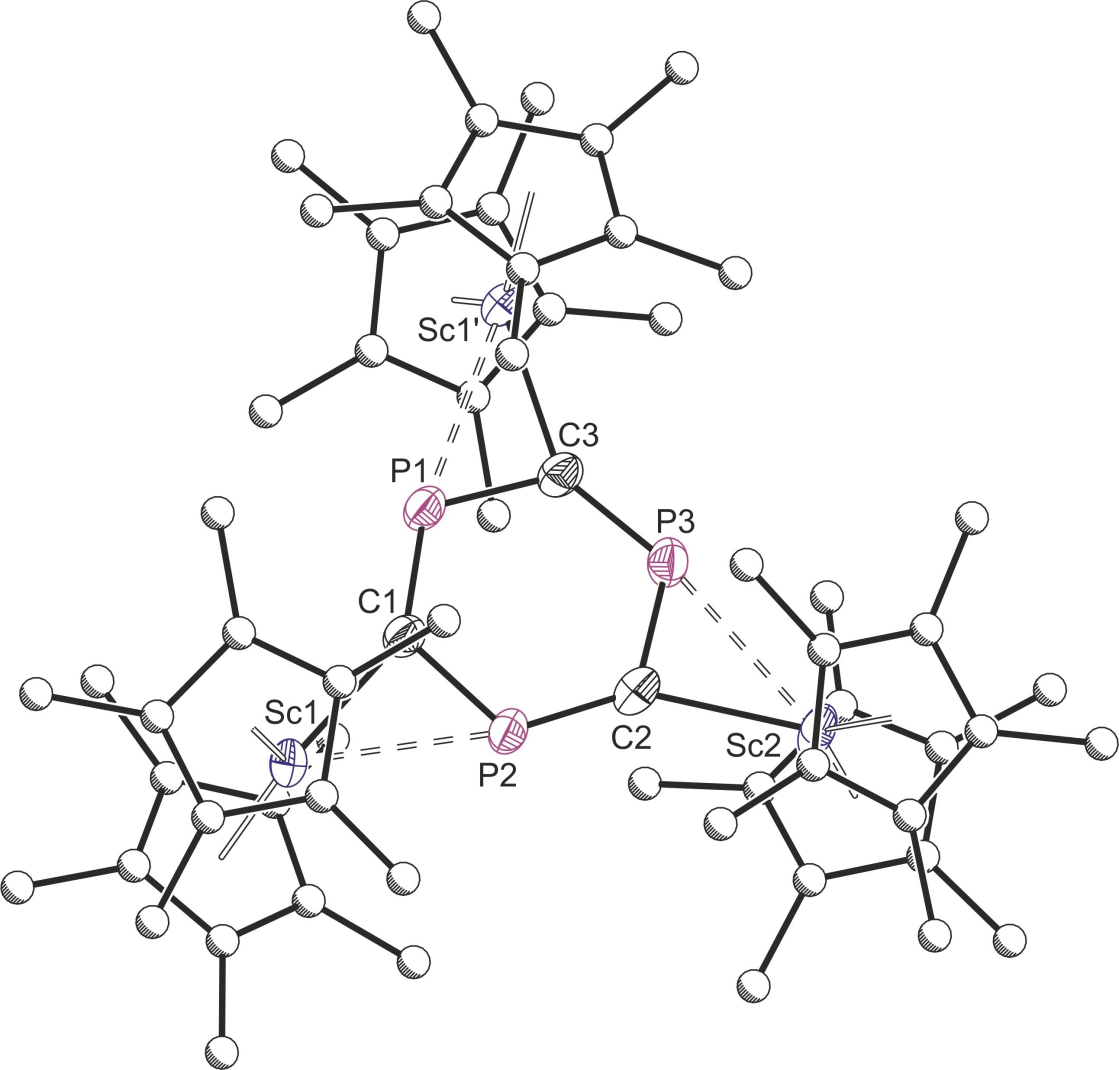
Solid‐state structure of **5**. Anisotropic displacement ellipsoids set at 50 % probability. Hydrogen atoms omitted for clarity. Selected bond lengths (Å) and angles (°): Sc1−C1: 2.221(6), Sc1−P2: 2.756(2), Sc2−C2: 2.263(7), Sc2−P3: 2.769(2), Sc1′−C3: 2.270(6), Sc1′−P1: 2.842(6), C1−P1: 1.718(8), C1−P2: 1.726(7), P1−C3: 1.746(8), C3−P3: 1.992(3), C2−P3: 2.253(1), C2−P2: ; 1.704(7); Sc1−C1−P1: 148.8(3), Sc1′−C3−P3: 147.7(3), Sc2−C2−P2: 148.8(4), C1−P1−C3: 116.1(3), P1−C3−P3: 123.1(3), C3−P3−C2: 116.7(4), P3−C2−P2: 124.1(4), C2−P2−C1: 116.4(3), P2−C1−P1: 123.4(3).

The attempted synthesis of the scandium(III) cyaphide complex Sc(Cp*)_2_(CP) (**4**) further demonstrates the considerable reactivity of the cyaphide ion. The reaction of **A** with Sc(Cp*)_2_Cl^[46]^ under slow‐diffusion conditions results in the formation of the trimeric product {Sc(Cp*)_2_}_3_(μ_3_‐C_3_P_3_) (**5**) as red crystals (Figure 5, top). Complex **5** shows a single broad resonance in its ^31^P{^1^H} NMR spectrum at 245.5 ppm (*ν*_1/2_=99 Hz), as well as a single Cp* environment in its ^1^H and ^13^C{^1^H} NMR spectra.

**Scheme 3:**
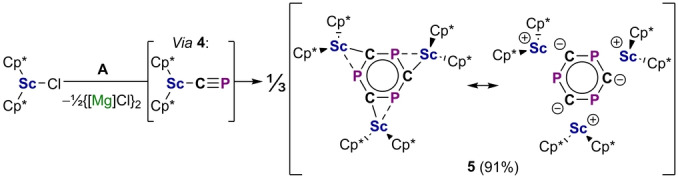
Synthesis of **5** by trimerization of proposed intermediate **4**.

The solid‐state structure of **5** reveals three Sc(Cp*)_2_ centers coordinated to a central C_3_P_3_^3−^ ion (Scheme 3). The C_3_P_3_^3−^ ring is disordered over two chemically equivalent positions in the solid‐state, preventing reliable discussion of its experimentally determined bond metrics. However, DFT calculations are in good agreement with the modeled disorder components and show that (in the gas phase) they are related by a low energy ring‐rotation barrier.

**Figure 6:**
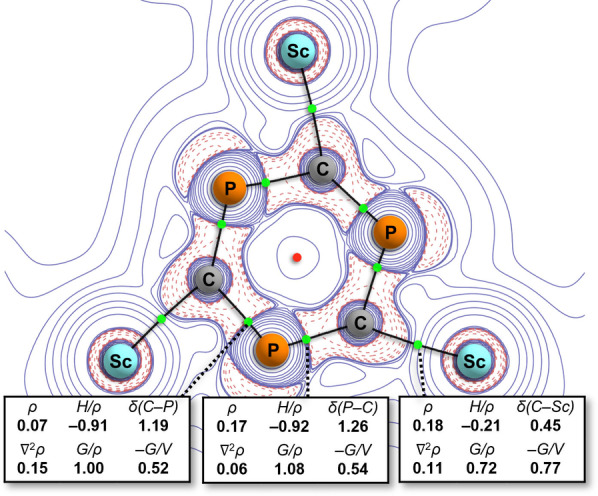
Quantum Theory of Atoms‐In‐Molecules (QTAIM) analysis of **5** (full details provided in the Supporting Information).

In both the solid‐state and calculated geometries, the C_3_P_3_^3−^ ring is planar. Nucleus‐Independent Chemical Shift (NICS) calculations suggest that the ring is aromatic (NICS(0)=−5.41 ppm; NICS(1)=−7.86 ppm; NICSZZ(0)=−12.49 ppm; NICSZZ(1)=−20.58 ppm) with six π electrons, consistent with the C_3_P_3_^3−^ ring being isolobal to benzene. Compared to 2,4,6‐tris(*tert*‐butyl)‐1,3,5‐triphosphabenzene,^[22]^ **5** has smaller P−C−P bond angles (XRD 124.1(4)°, Calcd. 124.8°; *t*Bu_3_C_3_P_3_: XRD 130.3(3)°, Calcd. 130.4°) (Figure S15), in line with the values calculated for the C_3_P_3_^3−^ ion (the computed Hirshfeld partial charges for **5** lie between those of C_3_P_3_^3−^ and *t*Bu_3_C_3_P_3_). To further investigate the bonding in **5**, a topological analysis of its DFT calculated electron density was carried out using the Quantum Theory of Atoms‐In‐Molecules (QTAIM) approach (Figure 6). This revealed highly polar Sc−C bonds with relatively low covalency as indicated by their delocalization index (δ(C−Sc)=0.45). Interestingly, no bond critical points or bond paths were found between scandium and phosphorus atoms, suggesting that the η^2^ bonding in **5** is largely due to relatively weak electrostatic interactions between the scandium ions and phosphorus lone pairs. This is consistent with the calculated low energy ring rotation barrier for **5** that gives rise to the two disorder components in its solid‐state structure as well as its broadened ^31^P{^1^H} NMR signal.

**Figure 7:**
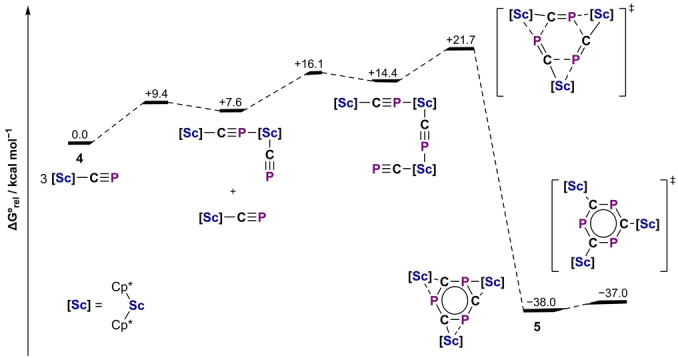
Calculated mechanism for the formation of **5** from **4** (ZORA‐ωB97X‐D3/ZORA‐def2‐TZVP). The barrier for C_3_P_3_^3−^ ring rotation relating the two disorder components in the solid‐state structure of **5** is also shown.

The formation of **5** suggests the intermediacy of a monomeric cyaphide complex Sc(Cp*)_2_(CP) (**4**). DFT calculations show that donor‐acceptor association of three coordinatively unsaturated monomer units facilitates a low barrier [2+2+2] cycloaddition reaction (Δ*G* ^≠^_calc_=21.7 kcal mol^−1^) to afford **5** (Figure 7). Notably, when **A** is instead reacted with the coordinatively saturated complex Sc(Cp*)_2_(THF)Cl, an intractable mixture of products results and compound **5** cannot be isolated, suggesting donor‐acceptor preorganization is important for favoring trimerization over other uncontrolled reactivity.

In comparison to the gold(I) cyaphide complex **B**, which has only been observed to oligomerize on reduction, the spontaneous trimerization of **4** stands in stark contrast. Neither **B** nor **4** afford the cyaphide ion any significant steric protection. However, the two complexes are opposites in terms of their M−C bond character. Scandium(III), an early 3d metal, is expected to form bonds with highly ionic character, contrasting the highly covalent M−C bonds formed by the late 5d metal gold(I). The Sc−CP bond in **4** has a substantially lower covalency than the Au−CP bond in **B**, as shown by their QTAIM delocalization indices (δ(Sc−C)=0.46, δ(Au−C)=1.13; Figure S17–S20). Their Natural Localized Molecular Orbital (NLMO) bond orders follow a similar pattern with a value of 0.31 for Sc−CP and a value of 0.41 for Au−CP (Table S2). In addition, Natural Bond Order analysis identifies a Au−CP covalently bonded Lewis structure for **B**, but instead describes the Sc−CP bond as a dative bond from a carbon‐centered lone pair (Table S2). These data suggest that the difference in reactivity between **B** and **4** may be due to the higher degree of electronic stabilization of the cyaphide ion by covalent bonding in **B** when compared to **4**, which is also reflected in a larger C≡P π–π* orbital energy gap (Figure S16). In addition, the coordinative assembly of **4** into Lewis acid‐base adducts in the calculated trimerization mechanism may further activate the C≡P bond. This suggests that strong Lewis acids would, in general, facilitate oligomerization.

Complex **2** falls between **B** and **4** in terms of M−C covalency. Nickel(II), a late 3d‐transition metal, is likely capable of forming stable cyaphide complexes given a suitable coordination environment (we recently reported an isolable cobalt(I) cyaphide complex supported by a NNN‐pincer ligand).^[37]^ However, in the case of **2** a different oligomerization pathway is available in which migratory insertion yields a transient η^2^‐phosphaallenide responsible for the subsequent cyclization to **3** (Figure 4). This observation, in combination with the recent work of Görlich et al.,^[34]^ implies that (at least for the group 10 metals) ligands coordinated to the metal through an sp^2^‐C should be avoided if the intention is to form stable M−CP complexes.

## Conclusion

To conclude, we have shown that the cyaphide ion is highly reactive in transition metal complexes, readily oligomerizing to form C_*n*_P_*n*_^*x*−^ units (*n*=2, *x*=2, 4; *n*=3, *x*=3). This mode of reactivity shows that the cyaphide ion can act as a synthetic precursor to larger conjugated organophosphorus molecules. The propensity for cyaphide oligomerization is heavily dependent on electronic stabilization, proceeding most readily for the ionic scandium(III) complex **4**. Importantly, this shows that the cyaphido ligand can remain reactive in the coordination sphere of metals with low steric protection, which will be of consequence in the design of cyaphide containing materials such as heavy analogues of Prussian Blue.

## Conflict of interest

The authors declare no conflict of interest.

1

## Supporting information

As a service to our authors and readers, this journal provides supporting information supplied by the authors. Such materials are peer reviewed and may be re‐organized for online delivery, but are not copy‐edited or typeset. Technical support issues arising from supporting information (other than missing files) should be addressed to the authors.

Supporting Information

Supporting Information

Supporting Information

## Data Availability

The data that support the findings of this study are available in the supplementary material of this article.
