# Supplementary material for: Metal‐Mediated Oligomerization Reactions of the Cyaphide Anion
Source: Angew Chem Int Ed Engl. 2023 Feb 1;62(11):e202218047. doi: 10.1002/anie.202218047 (PMC10946887; doi:10.1002/anie.202218047)

checkCIF/PLATON report

You have not supplied any structure factors. As a result the full set of tests cannot be run.

THIS REPORT IS FOR GUIDANCE ONLY. IF USED AS PART OF A REVIEW PROCEDURE FOR PUBLICATION, IT SHOULD NOT REPLACE THE EXPERTISE OF AN EXPERIENCED CRYSTALLOGRAPHIC REFEREE.

No syntax errors found. CIF dictionary Interpreting this report

Datablock: ESY373

```
Bond precision:   C-C = 0.0137 A          Wavelength=1.54184

Cell:             a=13.3207(2)     b=22.0522(3)        c=16.0911(2)
                  alpha=90         beta=96.152(1)      gamma=90
Temperature:      150 K
```

	Calculated	Reported
Volume	4699.55(11)	4699.55(11)
Space group	P 21/n	P 21/n
Hall group	-P 2yn	-P 2yn
Moiety formula	C96 H132 Au2 N4 P2 Sm2	C96 H132 Au2 N4 P2 Sm2
Sum formula	C96 H132 Au2 N4 P2 Sm2	C96 H132 Au2 N4 P2 Sm2
Mr	2098.66	2098.62
Dx,g cm-3	1.483	1.483
Z	2	2
Mu (mm-1)	15.617	15.617
F000	2096.0	2096.0
F000'	2063.07	
h,k,lmax	16,27,20	16,27,20
Nref	9873	9796
Tmin,Tmax	0.192,0.287	0.571,1.000
Tmin'	0.066	

```
Correction method= # Reported T Limits: Tmin=0.571 Tmax=1.000
AbsCorr = MULTI-SCAN

Data completeness= 0.992          Theta(max)= 76.427

R(reflections)= 0.0385( 8123)           wR2(reflections)= 0.0994( 9796)

S = 1.066                    Npar= 515
```

The following ALERTS were generated. Each ALERT has the format
test-name_ALERT_alert-type_alert-level.
Click on the hyperlinks for more details of the test.

Alert level B

PLAT220_ALERT_2_B NonSolvent Resd 1 C Ueq(max)/Ueq(min) Range 6.7 Ratio

Author Response: There is some positional disorder of the molecule in the asymmetric unit. A minor disordered component (approx. 10% occupancy) can be modeled for elements with large scattering factors (Au and Sm), however the disordered components of the light atoms could not be located in the Fourier difference map. This affects the carbene substituents in particular. This is an inherent issue with the sample as it was found for every crystal we analyzed.

PLAT242_ALERT_2_B Low 'MainMol' Ueq as Compared to Neighbors of C14 Check

Author Response: As above.

Alert level C

```
PLAT213_ALERT_2_C Atom C34             has ADP max/min Ratio .....         3.1 prolat
PLAT213_ALERT_2_C Atom C35             has ADP max/min Ratio .....         3.8 prolat
PLAT213_ALERT_2_C Atom C36             has ADP max/min Ratio .....         3.4 prolat
PLAT222_ALERT_3_C NonSolvent Resd 1  H   Uiso(max)/Uiso(min) Range         7.0 Ratio
PLAT230_ALERT_2_C Hirshfeld Test Diff for    C41      --C42      .         6.6 s.u.
PLAT234_ALERT_4_C Large Hirshfeld Difference C11      --C12      .        0.17 Ang.
PLAT242_ALERT_2_C Low    'MainMol' Ueq as Compared to Neighbors of         C11 Check
```

Author Response: As above.

PLAT242_ALERT_2_C Low 'MainMol' Ueq as Compared to Neighbors of C23 Check

Author Response: As above.

PLAT242_ALERT_2_C Low 'MainMol' Ueq as Compared to Neighbors of C26 Check

Author Response: As above.

PLAT242_ALERT_2_C Low 'MainMol' Ueq as Compared to Neighbors of C33 Check

Author Response: As above.

```
PLAT342_ALERT_3_C Low Bond Precision on  C-C Bonds ...............    0.01373 Ang.
PLAT360_ALERT_2_C Short  C(sp3)-C(sp3) Bond  C14      - C15      .       1.43 Ang.
PLAT601_ALERT_2_C Unit Cell Contains Solvent Accessible VOIDS of .         34 Ang**3
```

Alert level G

```
PLAT083_ALERT_2_G SHELXL Second Parameter in WGHT  Unusually Large      11.77 Why ?
PLAT232_ALERT_2_G Hirshfeld Test Diff (M-X)  Sm1      --P1       .       59.1 s.u.
PLAT232_ALERT_2_G Hirshfeld Test Diff (M-X)  Sm1      --P1_a     .       25.3 s.u.
PLAT232_ALERT_2_G Hirshfeld Test Diff (M-X)  Sm1A     --P1_a     .       18.1 s.u.
PLAT301_ALERT_3_G Main Residue  Disorder ..............(Resd  1  )         4% Note
PLAT367_ALERT_2_G Long?  C(sp?)-C(sp?) Bond  C29      - C34      .       1.51 Ang.
PLAT367_ALERT_2_G Long?  C(sp?)-C(sp?) Bond  C30      - C35      .       1.51 Ang.
PLAT367_ALERT_2_G Long?  C(sp?)-C(sp?) Bond  C31      - C36      .       1.52 Ang.
PLAT367_ALERT_2_G Long?  C(sp?)-C(sp?) Bond  C32      - C37      .       1.51 Ang.
PLAT367_ALERT_2_G Long?  C(sp?)-C(sp?) Bond  C40      - C45      .       1.52 Ang.
PLAT367_ALERT_2_G Long?  C(sp?)-C(sp?) Bond  C41      - C46      .       1.51 Ang.
PLAT367_ALERT_2_G Long?  C(sp?)-C(sp?) Bond  C42      - C47      .       1.51 Ang.
PLAT367_ALERT_2_G Long?  C(sp?)-C(sp?) Bond  C43      - C48      .       1.50 Ang.
```

---

0 **ALERT level A** = Most likely a serious problem - resolve or explain
2 **ALERT level B** = A potentially serious problem, consider carefully
13 **ALERT level C** = Check. Ensure it is not caused by an omission or oversight
13 **ALERT level G** = General information/check it is not something unexpected

0 ALERT type 1 CIF construction/syntax error, inconsistent or missing data
24 ALERT type 2 Indicator that the structure model may be wrong or deficient
3 ALERT type 3 Indicator that the structure quality may be low
1 ALERT type 4 Improvement, methodology, query or suggestion
0 ALERT type 5 Informative message, check

Datablock: EYNICP

Bond precision:	C-C = 0.0058 A		Wavelength=0.71073
Cell:	a=10.8597(2)	b=30.8769(5)	c=19.7884(4)
	alpha=90	beta=94.019(2)	gamma=90
Temperature:	150 K		

	Calculated	Reported
Volume	6619.0(2)	6619.0(2)
Space group	P 21/n	P 21/n
Hall group	-P 2yn	-P 2yn
Moiety formula	C66 H86 N4 Ni2 P2, C7 H8	C66 H86 N4 Ni2 P2, C7 H8
Sum formula	C73 H94 N4 Ni2 P2	C73 H94 N4 Ni2 P2
Mr	1206.85	1206.88
Dx,g cm-3	1.211	1.211
Z	4	4
Mu (mm-1)	0.661	0.661
F000	2584.0	2584.0
F000'	2588.17	
h,k,lmax	12,36,23	12,36,23
Nref	11650	11648
Tmin,Tmax	0.895,0.961	0.803,1.000
Tmin'	0.853	

Correction method= # Reported T Limits: Tmin=0.803 Tmax=1.000
AbsCorr = MULTI-SCAN

Data completeness= 1.000 Theta(max)= 24.999

R(reflections)= 0.0487(8775) wR2(reflections)= 0.1285(11648)

S = 1.075 Npar= 746

The following ALERTS were generated. Each ALERT has the format
test-name_ALERT_alert-type_alert-level.
Click on the hyperlinks for more details of the test.

Alert level C

```
PLAT220_ALERT_2_C NonSolvent   Resd 1  C   Ueq(max)/Ueq(min) Range        4.1 Ratio
PLAT231_ALERT_4_C Hirshfeld Test (Solvent)   C2S      --C7S      .        6.3 s.u.
PLAT231_ALERT_4_C Hirshfeld Test (Solvent)   C6S      --C7S      .        8.4 s.u.
PLAT232_ALERT_2_C Hirshfeld Test Diff (M-X)  Ni1      --C1       .        5.2 s.u.
PLAT234_ALERT_4_C Large Hirshfeld Difference C1S      --C2S      .       0.17 Ang.
PLAT234_ALERT_4_C Large Hirshfeld Difference C2S      --C3S      .       0.17 Ang.
PLAT234_ALERT_4_C Large Hirshfeld Difference C4S      --C5S      .       0.18 Ang.
PLAT241_ALERT_2_C High   'MainMol' Ueq as Compared to Neighbors of        C41 Check
PLAT242_ALERT_2_C Low    'MainMol' Ueq as Compared to Neighbors of        C52 Check
PLAT243_ALERT_4_C High   'Solvent' Ueq as Compared to Neighbors of        C4S Check
PLAT243_ALERT_4_C High   'Solvent' Ueq as Compared to Neighbors of        C6S Check
PLAT244_ALERT_4_C Low    'Solvent' Ueq as Compared to Neighbors of        C2S Check
PLAT244_ALERT_4_C Low    'Solvent' Ueq as Compared to Neighbors of        C5S Check
PLAT250_ALERT_2_C Large U3/U1 Ratio for Average U(i,j) Tensor ....        2.5 Note
PLAT260_ALERT_2_C Large Average Ueq of Residue Including       C1S      0.129 Check
PLAT332_ALERT_2_C Large Phenyl C-C Range     C2S       -C7S      .       0.18 Ang.
PLAT601_ALERT_2_C Unit Cell Contains Solvent Accessible VOIDS of .         44 Ang**3
```

Alert level G

```
PLAT002_ALERT_2_G Number of Distance or Angle Restraints on AtSite         15 Note
PLAT171_ALERT_4_G The CIF-Embedded .res File Contains EADP Records          5 Report
PLAT175_ALERT_4_G The CIF-Embedded .res File Contains SAME Records          2 Report
PLAT301_ALERT_3_G Main Residue  Disorder .............(Resd  1  )          7% Note
PLAT328_ALERT_4_G Possible Missing H on sp3? Phosphorus ..........         P1 Check
PLAT328_ALERT_4_G Possible Missing H on sp3? Phosphorus ..........         P2 Check
PLAT343_ALERT_2_G Unusual sp?      Angle Range in Main Residue for         C1 Check
PLAT380_ALERT_4_G Incorrectly? Oriented X(sp2)-Methyl Moiety .....        C1S Check
PLAT412_ALERT_2_G Short Intra XH3 .. XHn     H53A     ..H38A     .       2.06 Ang.
                                                    x,y,z  =      1_555 Check
PLAT720_ALERT_4_G Number of Unusual/Non-Standard Labels ..........          3 Note
PLAT779_ALERT_4_G Suspect or Irrelevant (Bond) Angle(s) in CIF ...      44.83 Deg.
              C2   -P1   -P2      1_555   1_555   1_555 ......     #   66 Check
PLAT779_ALERT_4_G Suspect or Irrelevant (Bond) Angle(s) in CIF ...      43.86 Deg.
              C1   -P1   -P2      1_555   1_555   1_555 ......     #   67 Check
PLAT779_ALERT_4_G Suspect or Irrelevant (Bond) Angle(s) in CIF ...      44.68 Deg.
              C1   -P2   -P1      1_555   1_555   1_555 ......     #   72 Check
PLAT779_ALERT_4_G Suspect or Irrelevant (Bond) Angle(s) in CIF ...      44.54 Deg.
              C2   -P2   -P1      1_555   1_555   1_555 ......     #   73 Check
PLAT790_ALERT_4_G Centre of Gravity not Within Unit Cell: Resd.  #          2 Note
              C7 H8
PLAT860_ALERT_3_G Number of Least-Squares Restraints .............         30 Note
PLAT941_ALERT_3_G Average HKL Measurement Multiplicity ...........        3.3 Low
PLAT965_ALERT_2_G The SHELXL WEIGHT Optimisation has not Converged     Please Check
PLAT967_ALERT_5_G Note: Two-Theta Cutoff Value in Embedded .res ..       50.0 Degree
```

---

0 **ALERT level A** = Most likely a serious problem - resolve or explain
0 **ALERT level B** = A potentially serious problem, consider carefully
17 **ALERT level C** = Check. Ensure it is not caused by an omission or oversight
19 **ALERT level G** = General information/check it is not something unexpected

0 ALERT type 1 CIF construction/syntax error, inconsistent or missing data
12 ALERT type 2 Indicator that the structure model may be wrong or deficient
3 ALERT type 3 Indicator that the structure quality may be low
20 ALERT type 4 Improvement, methodology, query or suggestion
1 ALERT type 5 Informative message, check

Datablock: ESY045

Bond precision: C-C = 0.0041 A Wavelength=1.54184

Cell:	a=20.7447(6)	b=14.3514(3)	c=20.3469(5)
	alpha=90	beta=105.036(3)	gamma=90
Temperature:	150 K		

	Calculated	Reported
Volume	5850.2(3)	5850.2(3)
Space group	C 2/c	C 2/c
Hall group	-C 2yc	-C 2yc
Moiety formula	C63 H90 P3 Sc3	C63 H90 P3 Sc3
Sum formula	C63 H90 P3 Sc3	C63 H90 P3 Sc3
Mr	1075.14	1075.13
Dx,g cm-3	1.221	1.221
Z	4	4
Mu (mm-1)	3.956	3.956
F000	2304.0	2304.0
F000'	2315.75	
h,k,lmax	24,17,24	24,17,24
Nref	5157	5157
Tmin,Tmax	0.653,0.789	0.790,1.000
Tmin'	0.432	

Correction method= # Reported T Limits: Tmin=0.790 Tmax=1.000
AbsCorr = MULTI-SCAN

Data completeness= 1.000 Theta(max)= 66.499

R(reflections)= 0.0532(4343) wR2(reflections)= 0.1593(5157)

S = 1.053 Npar= 355

The following ALERTS were generated. Each ALERT has the format
test-name_ALERT_alert-type_alert-level.
Click on the hyperlinks for more details of the test.

Alert level C

```
PLAT094_ALERT_2_C Ratio of Maximum / Minimum Residual Density ....       3.15 Report
PLAT413_ALERT_2_C Short Inter XH3 .. XHn     H20I      ..H20I     .       2.13 Ang.
                                                 1-x,-y,-z  =      5_655 Check
```

Alert level G

```
PLAT002_ALERT_2_G Number of Distance or Angle Restraints on AtSite          6 Note
PLAT172_ALERT_4_G The CIF-Embedded .res File Contains DFIX Records          3 Report
PLAT232_ALERT_2_G Hirshfeld Test Diff (M-X)  Sc1      --P2       .        6.0 s.u.
PLAT232_ALERT_2_G Hirshfeld Test Diff (M-X)  Sc2      --P3       .        8.0 s.u.
PLAT300_ALERT_4_G Atom Site Occupancy of P1          Constrained at       0.5 Check
PLAT300_ALERT_4_G Atom Site Occupancy of P2          Constrained at       0.5 Check
PLAT300_ALERT_4_G Atom Site Occupancy of P3          Constrained at       0.5 Check
PLAT300_ALERT_4_G Atom Site Occupancy of C1          Constrained at       0.5 Check
PLAT300_ALERT_4_G Atom Site Occupancy of C2          Constrained at       0.5 Check
PLAT300_ALERT_4_G Atom Site Occupancy of C3          Constrained at       0.5 Check
PLAT301_ALERT_3_G Main Residue  Disorder .............(Resd  1  )          9% Note
```

```
PLAT328_ALERT_4_G Possible Missing H on sp3? Phosphorus ..........         P2 Check
PLAT328_ALERT_4_G Possible Missing H on sp3? Phosphorus ..........         P3 Check
PLAT380_ALERT_4_G Incorrectly? Oriented X(sp2)-Methyl Moiety .....       C116 Check
PLAT789_ALERT_4_G Atoms with Negative _atom_site_disorder_group  #          6 Check
PLAT860_ALERT_3_G Number of Least-Squares Restraints ............          3 Note
PLAT933_ALERT_2_G Number of HKL-OMIT Records in Embedded .res File          5 Note
PLAT965_ALERT_2_G The SHELXL WEIGHT Optimisation has not Converged     Please Check
PLAT967_ALERT_5_G Note: Two-Theta Cutoff Value in Embedded .res ..      133.0 Degree
```

```
   0 ALERT level A = Most likely a serious problem - resolve or explain
   0 ALERT level B = A potentially serious problem, consider carefully
   2 ALERT level C = Check. Ensure it is not caused by an omission or oversight
  19 ALERT level G = General information/check it is not something unexpected

   0 ALERT type 1 CIF construction/syntax error, inconsistent or missing data
   7 ALERT type 2 Indicator that the structure model may be wrong or deficient
   2 ALERT type 3 Indicator that the structure quality may be low
  11 ALERT type 4 Improvement, methodology, query or suggestion
   1 ALERT type 5 Informative message, check
```

It is advisable to attempt to resolve as many as possible of the alerts in all categories. Often the minor alerts point to easily fixed oversights, errors and omissions in your CIF or refinement strategy, so attention to these fine details can be worthwhile. In order to resolve some of the more serious problems it may be necessary to carry out additional measurements or structure refinements. However, the purpose of your study may justify the reported deviations and the more serious of these should normally be commented upon in the discussion or experimental section of a paper or in the "special_details" fields of the CIF. checkCIF was carefully designed to identify outliers and unusual parameters, but every test has its limitations and alerts that are not important in a particular case may appear. Conversely, the absence of alerts does not guarantee there are no aspects of the results needing attention. It is up to the individual to critically assess their own results and, if necessary, seek expert advice.

Publication of your CIF in IUCr journals

A basic structural check has been run on your CIF. These basic checks will be run on all CIFs submitted for publication in IUCr journals (*Acta Crystallographica*, *Journal of Applied Crystallography*, *Journal of Synchrotron Radiation*); however, if you intend to submit to *Acta Crystallographica Section C* or *E* or *IUCrData*, you should make sure that full publication checks are run on the final version of your CIF prior to submission.

Publication of your CIF in other journals

Please refer to the *Notes for Authors* of the relevant journal for any special instructions relating to CIF submission.

PLATON version of 28/11/2022; check.def file version of 28/11/2022

Datablock ESY373 - ellipsoid plot

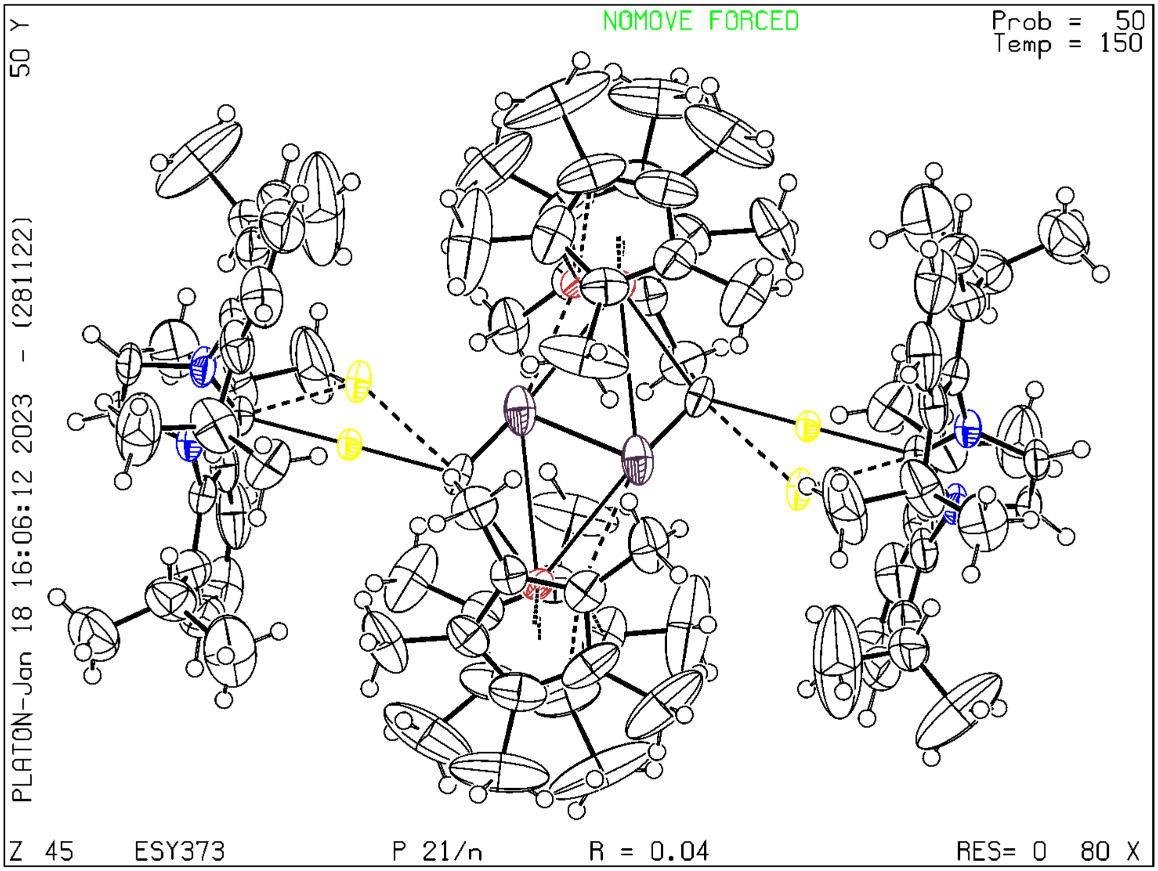

Datablock EYNICP - ellipsoid plot

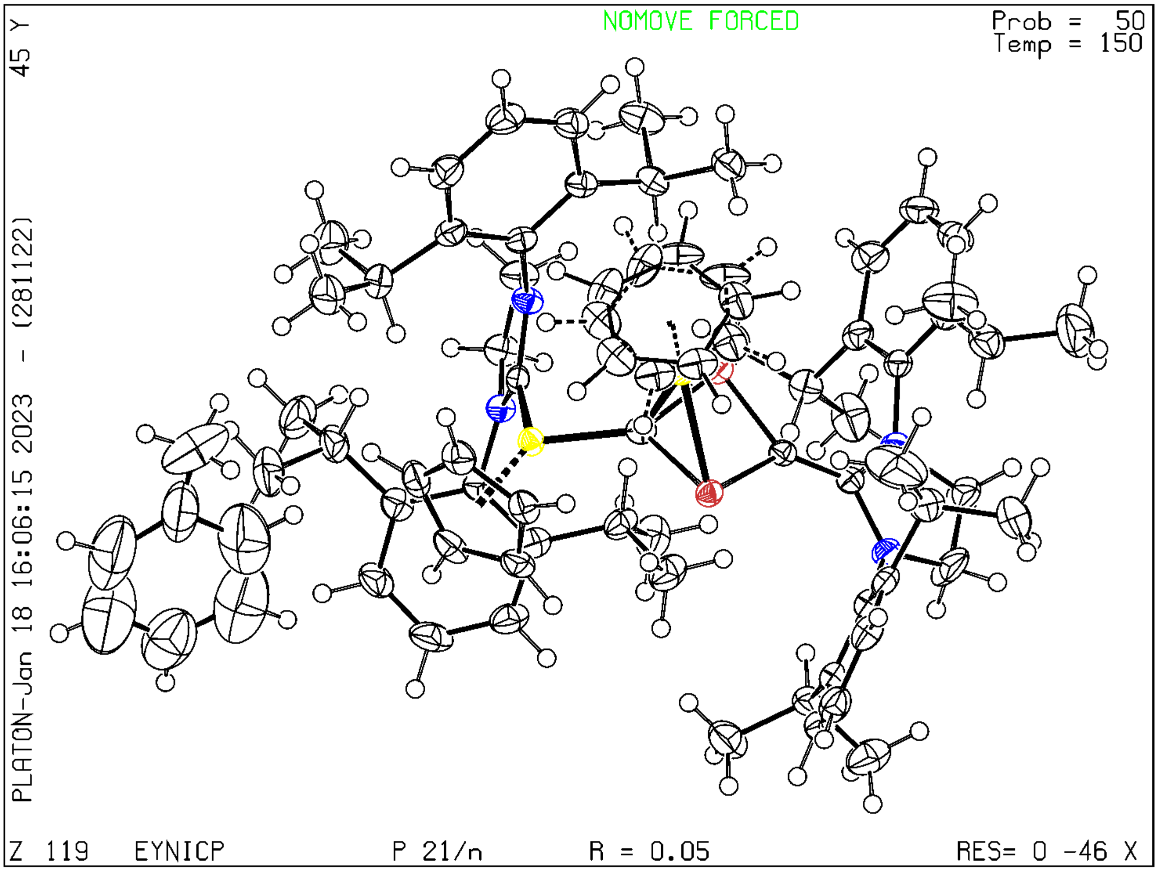

Datablock ESY045 - ellipsoid plot

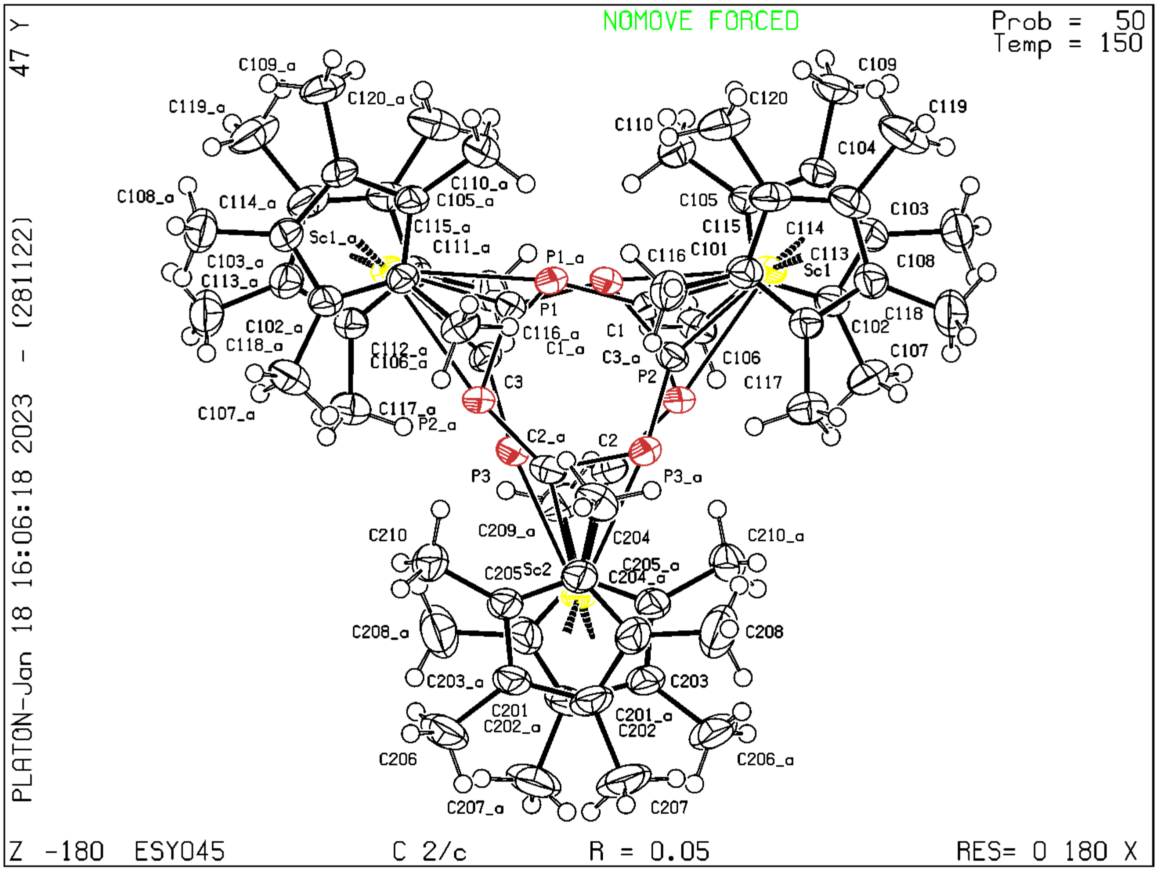

Supplement: Supplementary file 2 — Supporting Information [file ANIE-62-0-s001.pdf]
