# Supplementary material for: Metal‐Mediated Oligomerization Reactions of the Cyaphide Anion
Source: Angew Chem Int Ed Engl. 2023 Feb 1;62(11):e202218047. doi: 10.1002/anie.202218047 (PMC10946887; doi:10.1002/anie.202218047)
Supplement: Supplementary file 3 — Supporting Information [file ANIE-62-0-s003.pdf]

Angewandte Chemie
Eine Zeitschrift der Gesellschaft Deutscher Chemiker

Supporting Information

Metal-Mediated Oligomerization Reactions of the Cyaphide Anion

*E. S. Yang, D. W. N. Wilson, J. M. Goicoechea**

Contents

1. Experimental section 2
  1.1 General experimental methods 2
  1.2 Synthesis of reported compounds 3
2. Single crystal X-ray diffraction data 12
3. Computational details 13
  3.1. General computational methods 13
  3.2. Computed mechanisms 14
  3.3. Electronic structure calculations 15
  3.4. Topological analysis 17
  3.5. XYZ coordinates 21
4. References 44

1. Experimental section

1.1 General experimental methods

Synthetic methods. All reactions and product manipulations were carried out using standard Schlenk-line techniques under an inert atmosphere of argon, or in a dinitrogen filled glovebox (MBraun UNIlab glovebox maintained at < 0.1 ppm H_2O and < 0.1 ppm O_2). Au(IDipp)(CP),[1] Sm(Cp*)$_2$(OEt$_2$),[2] Ni(SIDipp)(Cp)Cl,[3] and Sc(Cp*)$_2$Cl[4] were synthesized according to previously reported synthetic procedures. Mg(^{Dipp}NacNac)(dioxane)(CP) was generated *in situ* according to a previously reported procedure.[1] Toluene (Sigma Aldrich HPLC grade), hexane (Sigma Aldrich HPLC grade), and pentane (Sigma Aldrich HPLC grade) were purified using an MBraun SPS-800 solvent system. THF (Sigma Aldrich HPLC grade) was distilled over sodium/benzophenone. C_6D_6 (Aldrich, 99.5%) was degassed dried over CaH_2. d_6-DMSO (Aldrich, 99.5%) was degassed and dried twice over activated 3 Å molecular sieves. All dry solvents were stored under argon in gas-tight ampoules over activated 3 Å molecular sieves.

Characterization techniques. NMR spectra were acquired on a Bruker AVIII 400 MHz NMR spectrometer (^{1}H 400 MHz, ^{31}P 162 MHz), Bruker AVIII 500 MHz NMR spectrometer (^{1}H 500 MHz, ^{13}C 126 MHz, ^{31}P 202 MHz) or a Bruker Avance NEO 600 MHz NMR spectrometer with a broadband helium cryoprobe (^{13}C 151 MHz). ^{1}H and ^{13}C NMR spectra were referenced to the most downfield solvent resonance (^{1}H NMR C_6D_6: δ = 7.16 ppm, ^{13}C NMR C_6D_6: δ = 128.06 ppm; ^{1}H NMR d_6-DMSO: δ = 2.50 ppm, ^{13}C NMR d_6-DMSO: δ = 39.52 ppm.). ^{31}P NMR spectra were externally referenced to an 85% solution of H_3PO_4 in H_2O. Infrared spectra were acquired on a Thermo Scientific iS5 FTIR spectrometer using an iD3 ATR stage. Elemental analyses were carried out by Elemental Microanalyses Ltd. (Devon, U.K.) or by London Metropolitan University (London, U.K.). Samples (approx. 5 mg) were submitted in flame sealed glass tubes.

1.2 Synthesis of reported compounds

1.2.1 Synthesis of {Au(IDipp)}$_2${Sm(Cp*)$_2$}$_2$(μ$_4$-C_2P_2) (1)

In a long, thin ampoule, a solution of Sm(Cp*)$_2$(OEt$_2$) (40 mg, 0.08 mmol) in toluene (1 mL) was carefully layered on top of a solution of Au(IDipp)(CP) (50 mg, 0.08 mmol) in toluene (1 mL). The solutions were allowed to mix by diffusion at room temperature over 5 days, resulting in the formation of large brown crystals of {Au(IDipp)}$_2${Sm(Cp*)$_2$}$_2$(μ$_4$-C_2P_2) suitable for X-ray crystallography. The supernatant solution was decanted, and the crystals washed with toluene (2 × 2 mL) and pentane (2 × 2 mL), then dried under vacuum. Yield: 74 mg, 0.07 mmol, 88%. Anal. Calcd. (%) for $C_{56}H_{72}Au_2N_4P_2Sm_2$: C, 54.94; H, 6.34; N, 2.67. Found: C, 54.68; H, 6.08; N, 2.26. The product is insoluble in all common organic solvents, including polar, coordinating organic solvents such as acetonitrile and DMSO, precluding characterization by NMR spectroscopy.

1.2.2 Synthesis of Ni(SIDipp)(Cp)(CP) (2)

Mg(^{Dipp}Nacnac)(dioxane)(CP) (approx. 130 mg, 0.23 mmol) was generated *in situ* in toluene (1 mL). Ni(SIDipp)(Cp)Cl (105 mg, 0.19 mmol) was added as a solid to the solution of Mg(^{Dipp}Nacnac)(dioxane)(CP), and the mixture stirred at room temperature for 4 hours. The mixture was filtered, and the solvent removed from the filtrate *in vacuo*. The residue was extracted with hexane (2 mL), which was then concentrated and stored at −35 °C for 1 day, yielding Ni(SIDipp)(Cp)(CP) as an off-white, microcrystalline solid. The crystals were isolated by filtration, and washed with cold hexane (2 × 0.5 mL). Yield: 72 mg, 0.13 mmol, 68%. Anal. Calcd. (%) for $C_{33}H_{43}N_2NiP$: C, 71.11; H, 7.78; N, 5.03. Found: C, 71.53; H, 8.47; N, 4.77.

^{1}H NMR (400 MHz, C_6D_6): δ(ppm) 7.26 (dd, $^3J_{H–H}$ = 8.6, 7 Hz, 2H, Dipp *para* C**H**), 7.20–7.16 (m, 4H, Dipp *meta* C**H**), 4.75 (s, 5H, Cp C_5**H_5**), 3.55 (sept, $^3J_{H–H}$ = 7 Hz, 4H, Dipp C**H**(CH_3)$_2$), 3.55 (s, 4H, SIDipp C_2**H_4**), 1.53 (d, $^3J_{H–H}$ = 7 Hz, 12H, Dipp CH(C**H_3**)(CH_3), 1.09 (d, $^3J_{H–H}$ = 7 Hz, 12H, Dipp CH(CH_3)(C**H_3**).

^{13}C{^{1}H} NMR (126 MHz, C_6D_6): δ(ppm) 224.02 (d, $^1J_{C–P}$ = 10 Hz, Ni*C*P), 211.39 (s, SIDipp (H_2CN(Dipp))$_2$**C**Ni), 147.91 (s, Dipp *para* **C**), 138.03 (s, Dipp *ipso* **C**), 129.56 (s, Dipp *ortho* **C**), 124.67 (s, *meta* **C**), 92.07 (s, Cp **C_5**H_5), 53.64 (s, SIDipp (H_2**C**N(Dipp))$_2$C), 28.70 (s, Dipp **C**H(CH_3)$_2$), 26.83 (s, Dipp CH(**C**H_3)(CH_3)), 24.42 (s, Dipp CH(CH_3)(**C**H_3)).

^{31}P{^{1}H} NMR (162 MHz, C_6D_6): δ(ppm) 181.2 (C≡**P**).

ATR-FTIR: 1307 cm^{-1} (m, *v*(C≡P)).

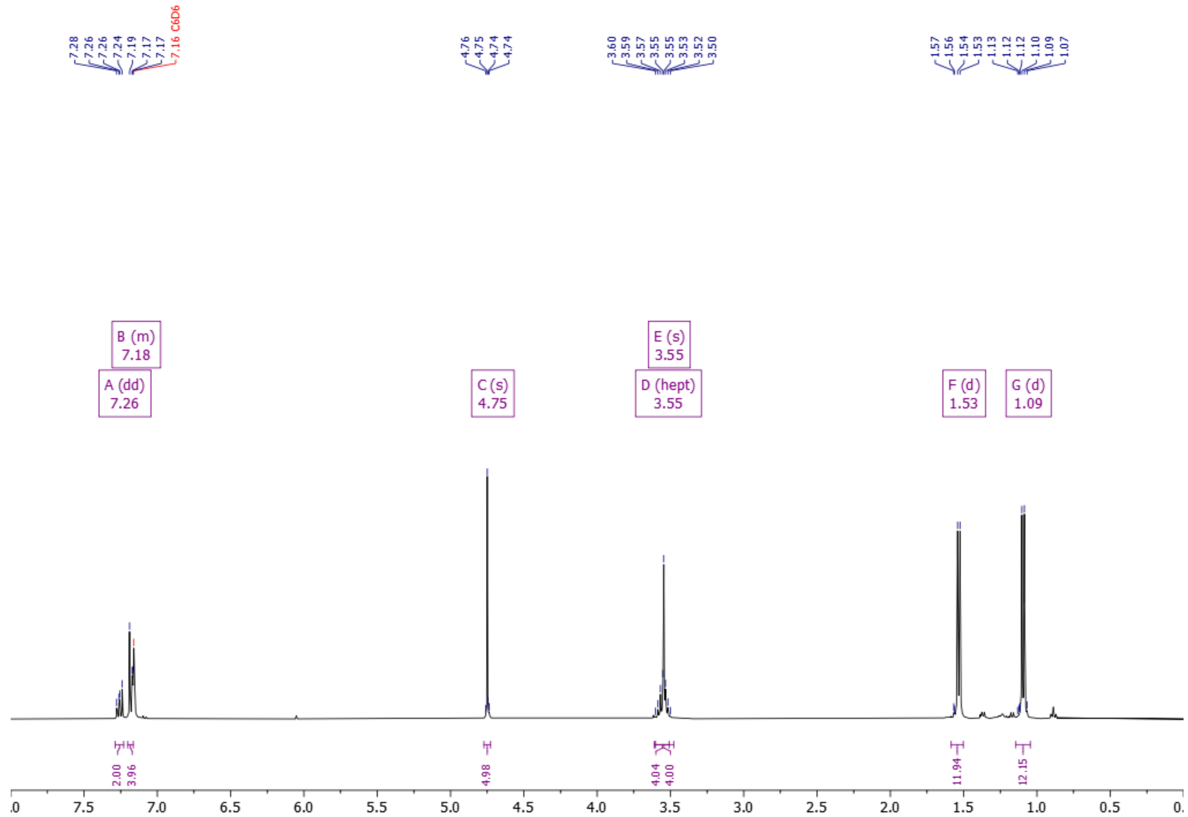

Figure S1. ^{1}H NMR (400 MHz) spectrum of **2** in C_6D_6.

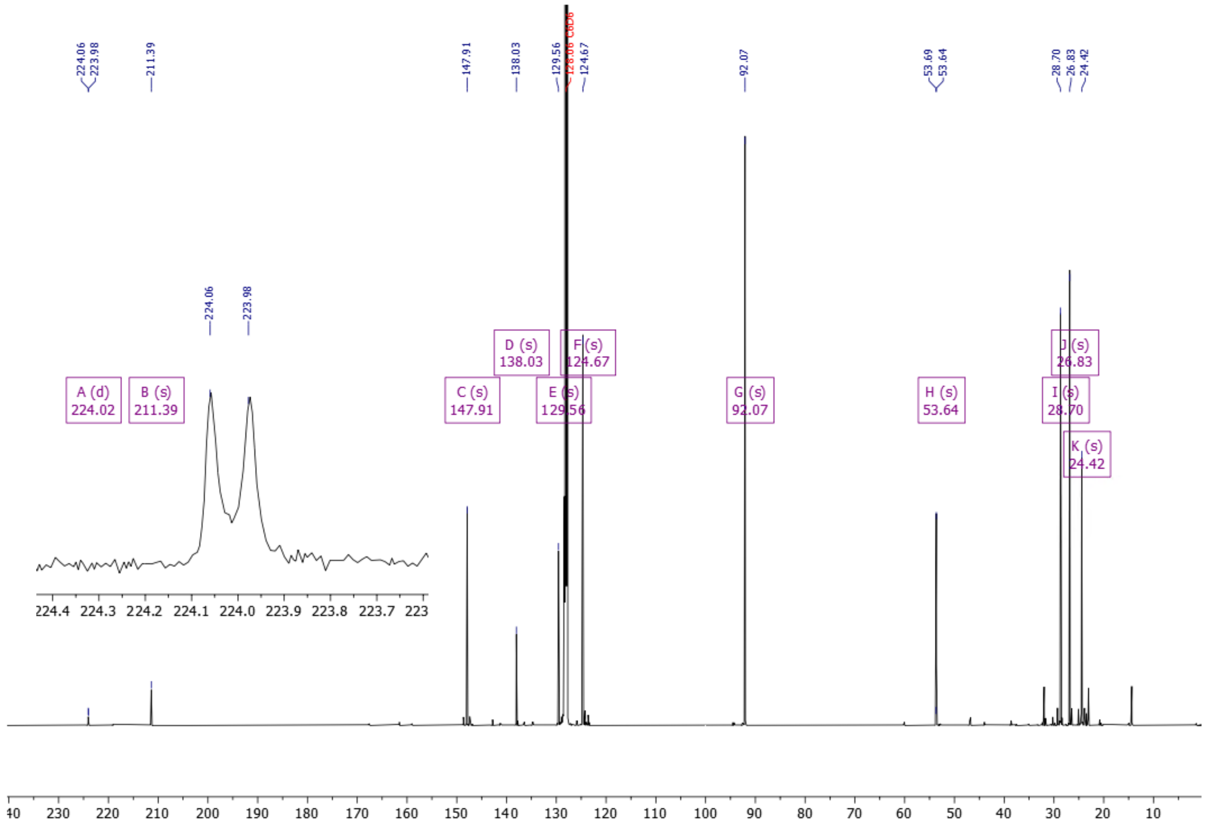

Figure S2. ^{13}C{^{1}H} NMR (126 MHz) spectrum of **2** in C_6D_6.

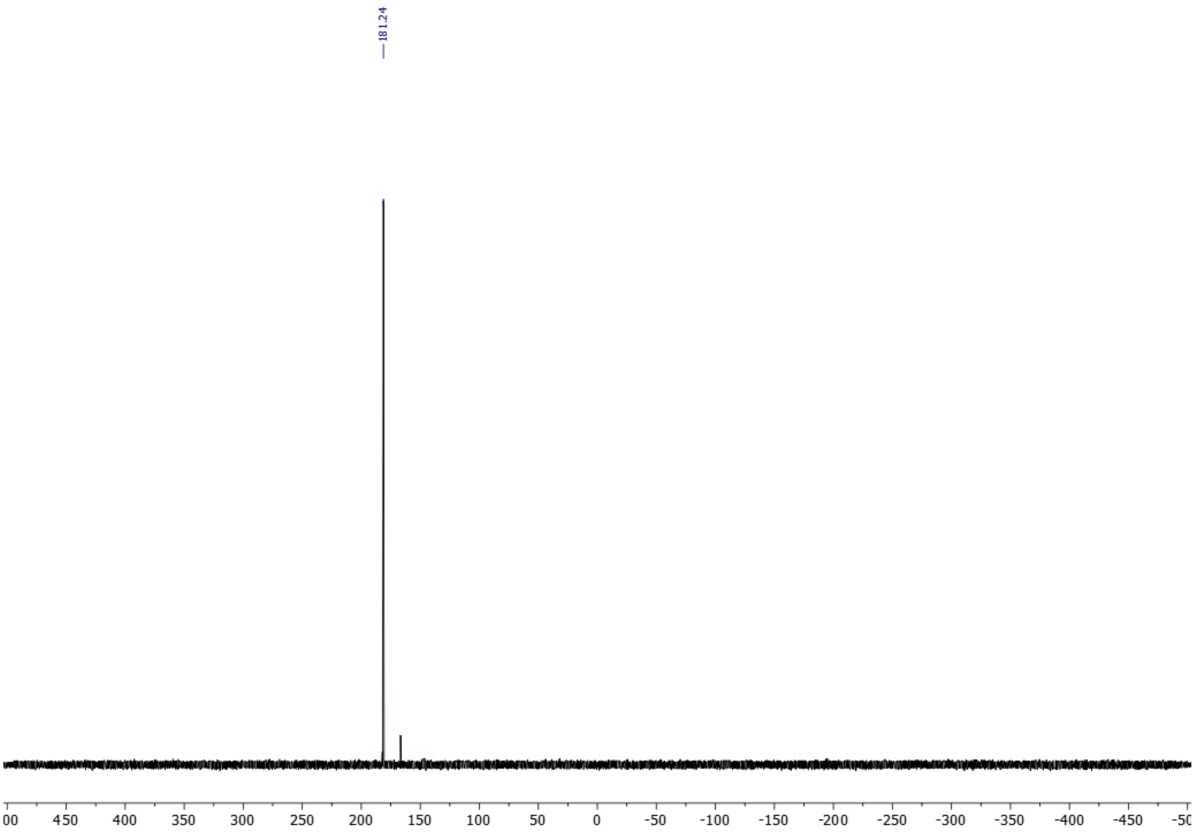

Figure S3. $^{31}P\{^{1}H\}$ NMR (162 MHz) spectrum of **2** in C_6D_6.

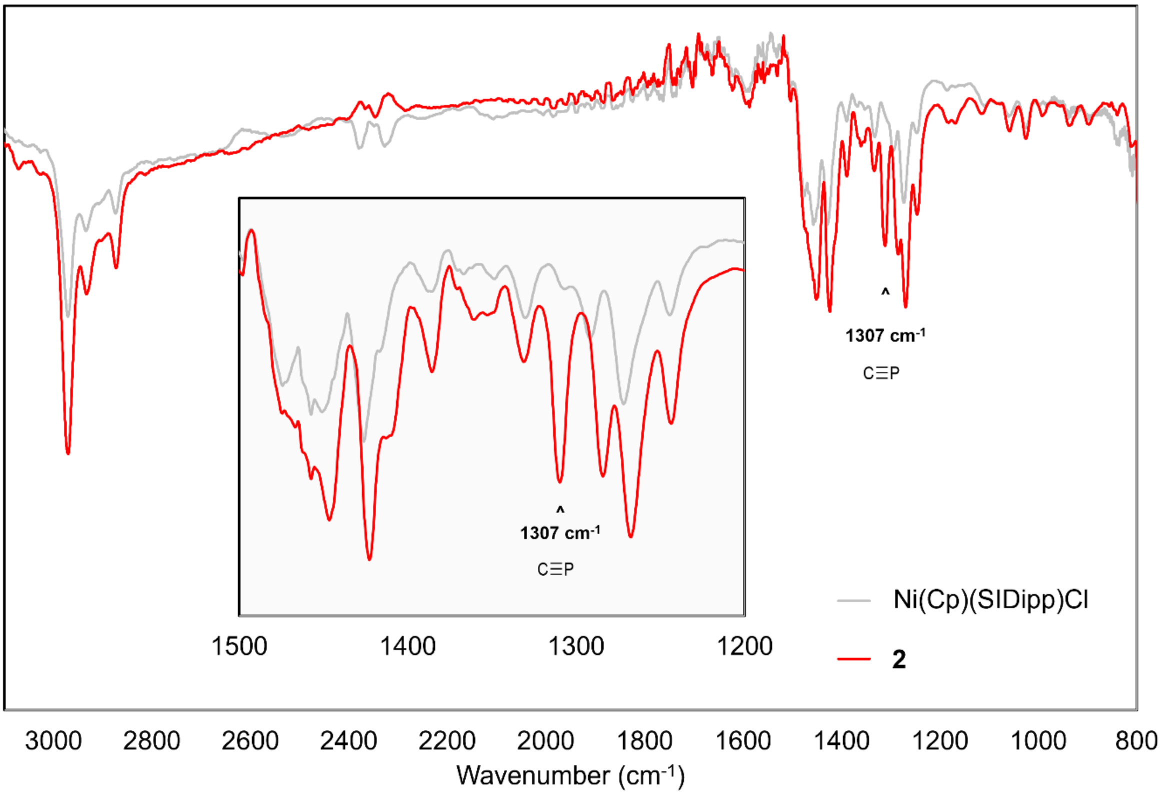

Figure S4. Solid-state ATR-FTIR spectrum of **2**.

1.2.3 Synthesis of {Ni(SIDipp)(Cp)}{Ni(Cp)}{μ$_2$-(SIDipp)C_2P_2} (3)

A solution of **2** (20 mg, 0.04 mmol) in toluene (0.5 mL) was concentrated by slow evaporation at room temperature over the course of 1 week, during which {Ni(SIDipp)(Cp)}{Ni(Cp)}{μ$_2$-(SIDipp)C_2P_2} crystallizes as black crystals suitable for X-ray crystallography. The crystals were isolated by filtration and washed with cold hexane (2 × 0.5 mL). Storage of the combined toluene and hexane solutions at −35 °C for 1 week yielded a second crop of crystals. Combined yield: 13 mg, 0.02 mmol, 64%. Anal. Calcd. (%) for $C_{66}H_{86}N_4Ni_2P_2 \cdot C_7H_8$: C, 72.65; H, 7.85; N 4.64. Found: C 73.35; H, 8.33; N, 4.17. The low symmetry of the complex, the broad appearance of peaks, as well as some overlap with the residual solvent signal prevented the complete explicit assignment of ^{1}H and ^{13}C spectra. However, in most cases the local chemical environment could be assigned to peaks using HSQC and HMBC 2D spectra.

^{1}H NMR (400 MHz, C_6D_6): δ(ppm) 7.62 (s, 1H, Dipp Ar-**H**), 7.30 (m, 4H, Dipp Ar-**H**), 7.08 (m, 6H, Dipp Ar-**H**), (s, 1H, Dipp Ar-**H**), 5.11 (s, 5H, Cp $C_5\mathbf{H}_5$), 4.68 (s, 5H, Cp $C_5\mathbf{H}_5$), 3.95 (b, 1H, SIDipp $C_2\mathbf{H}_4$), 3.77 (b, 2H, Dipp C**H**$(CH_3)_2$), 3.66 (b, 1H, (b, 1H, SIDipp $C_2\mathbf{H}_4$), 3.53 (m, 2H, SIDipp $C_2\mathbf{H}_4$), 3.45 (m, 2H, Dipp C**H**$(CH_3)_2$), 3.37 (m, 1H, SIDipp $C_2\mathbf{H}_4$), 3.15 (m, 1H, SIDipp $C_2\mathbf{H}_4$), 3.05 (b, 1H, SIDipp $C_2\mathbf{H}_4$), 2.83 (b, 1H, Dipp C**H**$(CH_3)_2$), 2.79 (b, 1H, Dipp C**H**$(CH_3)_2$), 2.51 (m, 2H, Dipp C**H**$(CH_3)_2$), 1.81 (b, 6H, Dipp CH(C**H**$_3$)$_2$), 1.68 (m, 3H, Dipp CH(C**H**$_3$)$_2$), 1.59 (m, 3H, Dipp CH(C**H**$_3$)$_2$), 1.55 (m, 3H, Dipp CH(C**H**$_3$)$_2$), 1.33 (b, 6H, Dipp CH(C**H**$_3$)$_2$), 1.24 (m, 3H, Dipp CH(C**H**$_3$)$_2$), 1.08 (m, 3H, Dipp CH(C**H**$_3$)$_2$), 1.03 (m, 3H, Dipp CH(C**H**$_3$)$_2$), 0.94 (m, 3H, Dipp CH(C**H**$_3$)$_2$), 0.84 (m, 3H, Dipp CH(C**H**$_3$)$_2$).

^{13}C{^{1}H} NMR (151 MHz, C_6D_6): δ(ppm) 217.48 (s, SIDipp $(H_2CNDipp)_2$**C**), 149.96 (s, Dipp Ar-**C**), 148.85 (s, Dipp Ar-**C**), 147.61 (s, Dipp Ar-**C**), 146.71 (s, Dipp Ar-**C**), 140.78 (s, Dipp Ar-**C**), 140.12 (s, Dipp Ar-**C**), 139.40 (s, Dipp Ar-**C**), 138.08 (s, Dipp Ar-**C**), 136.91 (s, Dipp Ar-**C**), 125.81 (s, Dipp Ar-**C**), 125.45 (s, Dipp Ar-**C**), 124.99 (s, Dipp Ar-**C**), 123.81 (m, **C**$_2P_2$), 94.56 (s, Cp **C**$_5H_5$), 92.04 (s, Cp **C**$_5H_5$), 54.99 (s, SIDipp (H_2**C**NDipp)$_2$C), 52.84 (s, SIDipp (H_2**C**NDipp)$_2$C), 51.63 (s, SIDipp (H_2**C**NDipp)$_2$C), 29.51 (s, Dipp **C**H(**C**H$_3$)$_2$), 29.07 (s, Dipp **C**H(**C**H$_3$)$_2$), 28.61 (s, Dipp **C**H(**C**H$_3$)$_2$), 28.35 (s, Dipp **C**H(**C**H$_3$)$_2$), 27.95 (s, Dipp **C**H(**C**H$_3$)$_2$), 27.09 (s, Dipp **C**H(**C**H$_3$)$_2$), 26.90 (s, Dipp **C**H(**C**H$_3$)$_2$), 26.43 (s, Dipp **C**H(**C**H$_3$)$_2$), 25.95 (s, Dipp **C**H(**C**H$_3$)$_2$), 25.43 (s, Dipp **C**H(**C**H$_3$)$_2$).

^{31}P{^{1}H} NMR (162 MHz, C_6D_6): δ(ppm) 130.3 (d, $^2J_{P–P}$ = 65 Hz), 119.9 (d, $^2J_{P–P}$ = 65 Hz).

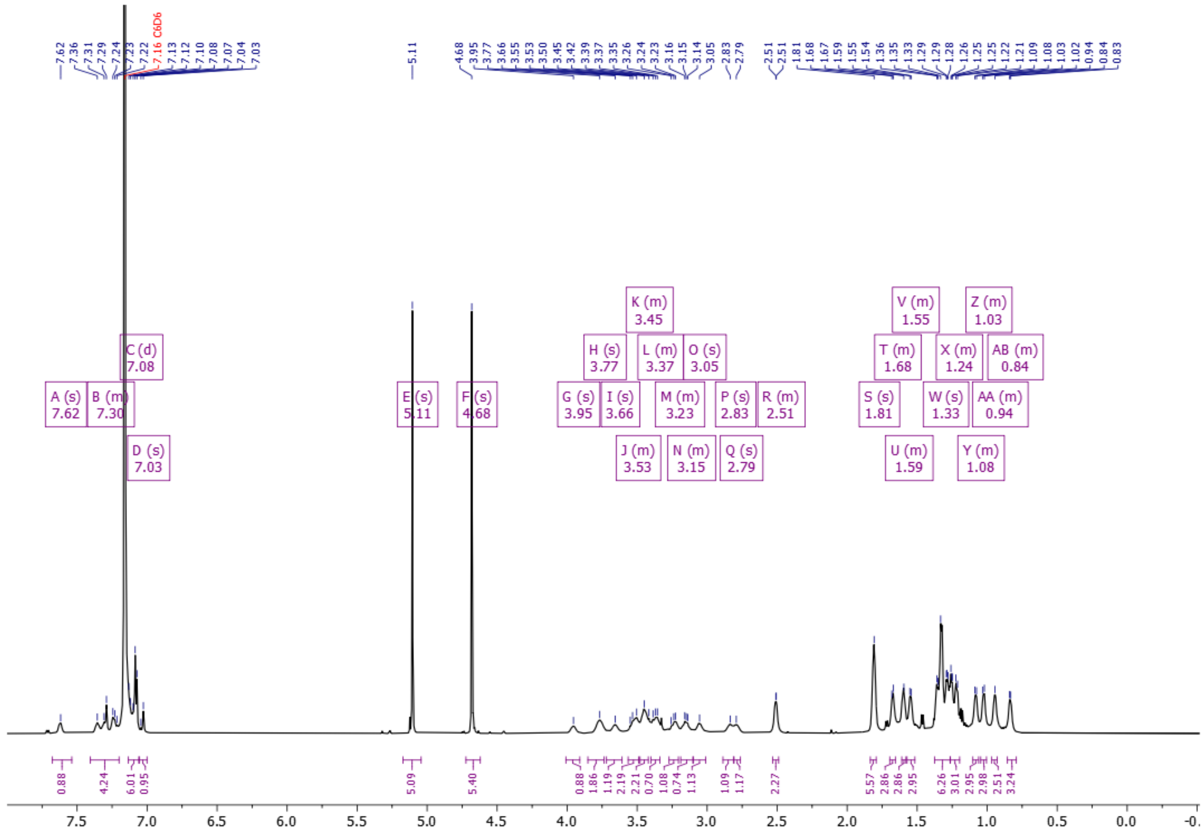

Figure S5. ^{1}H NMR (400 MHz) spectrum of **3** in C_6D_6.

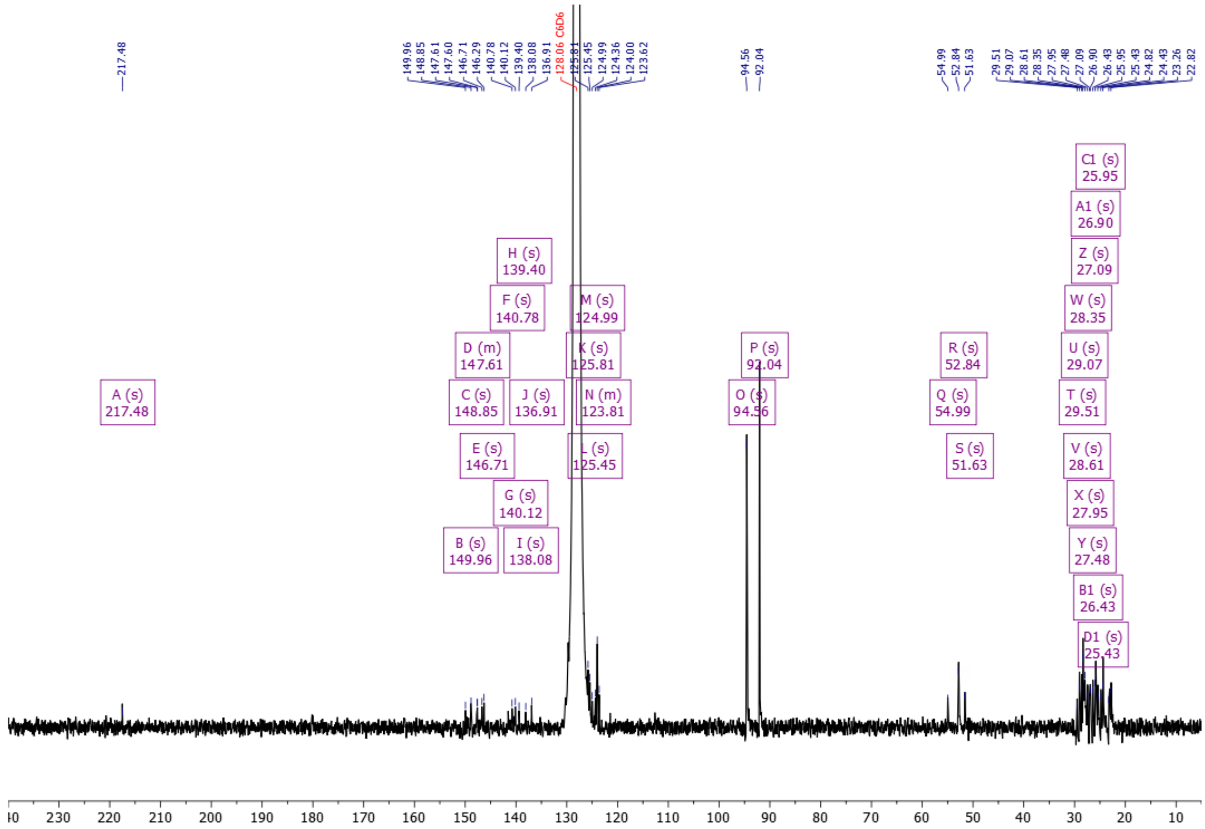

Figure S6. ^{13}C{^{1}H} NMR (151 MHz) spectrum of **3** in C_6D_6.

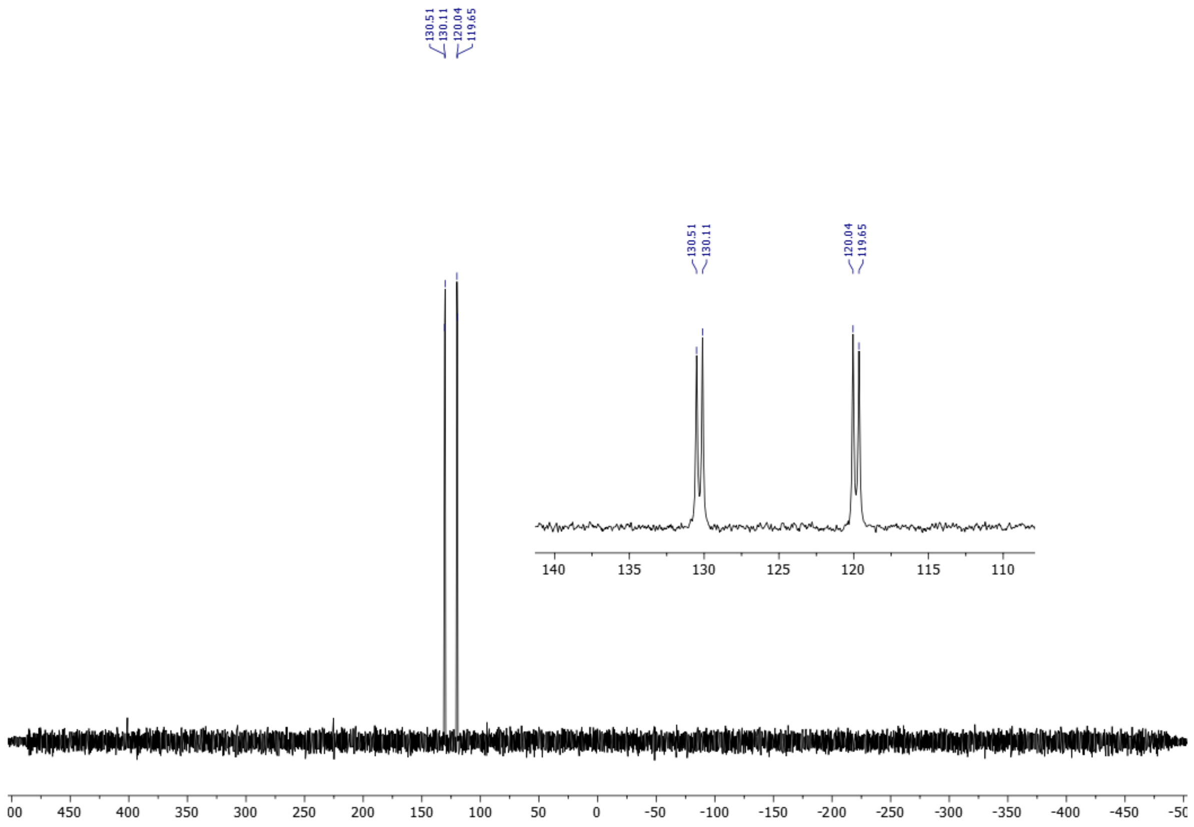

Figure S7. $^{31}P\{^1H\}$ NMR (162 MHz) spectrum of **3** in C_6D_6.

1.2.4 Synthesis of $\{Sc(Cp^*)_2\}_3(\mu_3\text{-}C_3P_3)$ (5)

Mg(^{Dipp}Nacnac)(dioxane)(CP) (approx. 321 mg, 0.56 mmol) was generated *in situ* in toluene (3 mL). A solution of $Sc(Cp^*)_2Cl$ (150 mg, 0.43 mmol) in hexane (5 mL) was carefully layered on top of the solution of Mg(^{Dipp}Nacnac)(dioxane)(CP) in a long, thin ampoule. The solutions were allowed to mix by diffusion at room temperature over 2 weeks, resulting in the formation of $\{Sc(Cp^*)_2\}_3(\mu_3\text{-}C_3P_3)$ as red, rod-like crystals suitable for X-ray crystallography. The crystals were isolated by filtration, and washed with THF (3 × 5 mL) to remove residual {Mg(^{Dipp}Nacnac)Cl}$_2$. Yield: 140 mg, 0.13 mmol, 91%. Anal. Calcd. (%) for $C_{63}H_{90}P_3Sc_3$: C, 70.38; H, 8.44; N, 0.00. Found: C, 69.87; H, 8.09; N, 0.00. The product is insoluble in many common organic solvents with the exception of DMSO, in which it decomposes over the course of several hours.

^{1}H NMR (500 MHz, d$_6$-DMSO): δ(ppm) 1.82 (s, 90H, (Cp* $C_5(C\mathbf{H}_3)_5$)).

$^{13}C\{^1H\}$ NMR (126 MHz, d$_6$-DMSO): δ(ppm) 115.75 (Cp* $\mathbf{C}_5(CH_3)_5$), 13.25 (Cp* $C_5(\mathbf{C}H_3)_5$).

$^{31}P\{^1H\}$ NMR (202 MHz, d$_6$-DMSO): δ(ppm) 245.5 (b, $v_{1/2}$ = 99 Hz, $C_3\mathbf{P}_3$).

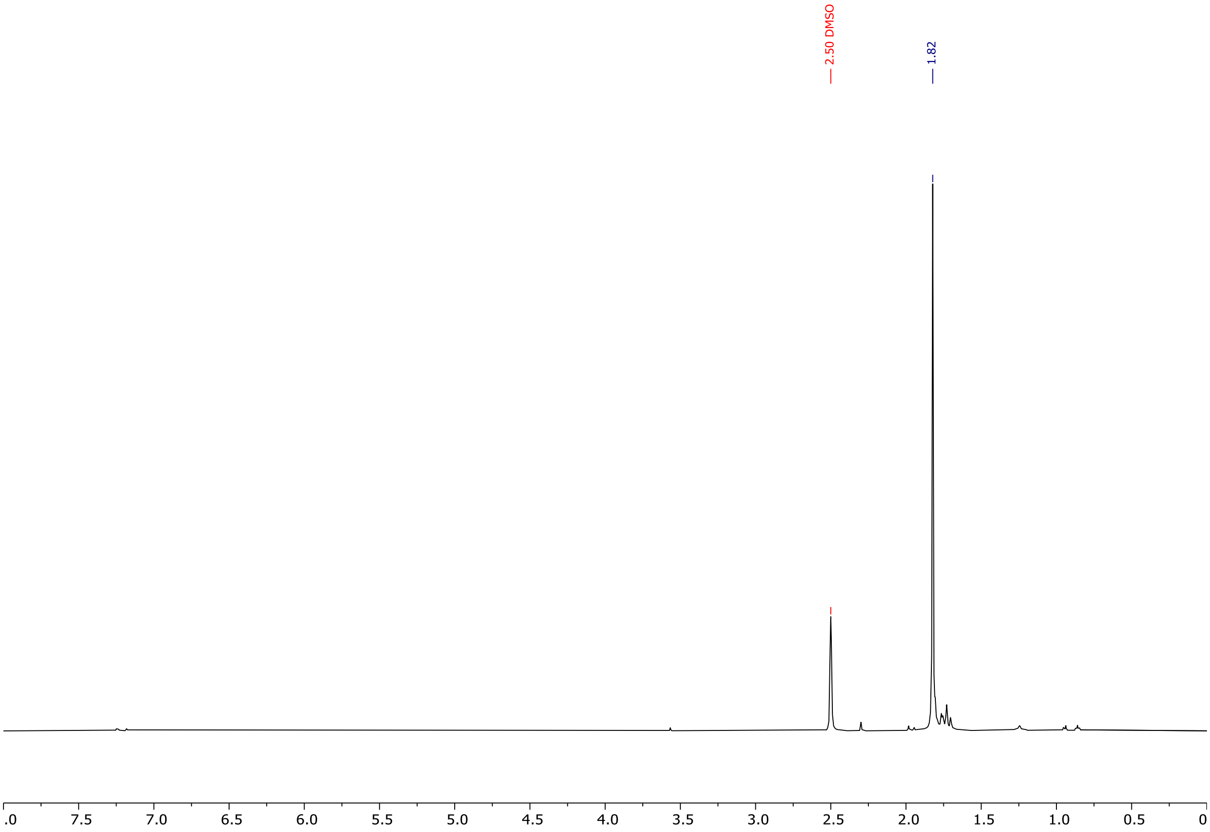

Figure S8. ^{1}H NMR (500 MHz) spectrum of **5** in d_6-DMSO.

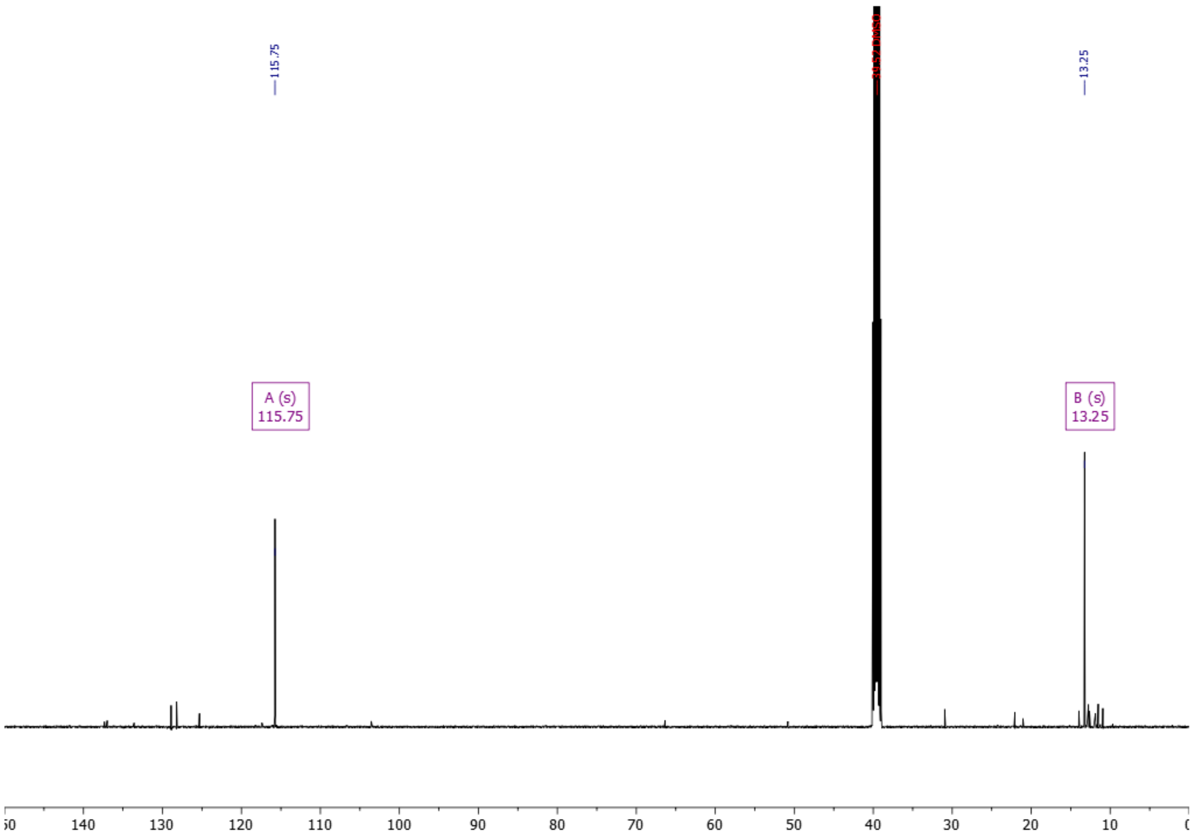

Figure S9. $^{13}C\{^{1}H\}$ NMR (126 MHz) spectrum of **5** in d_6-DMSO.

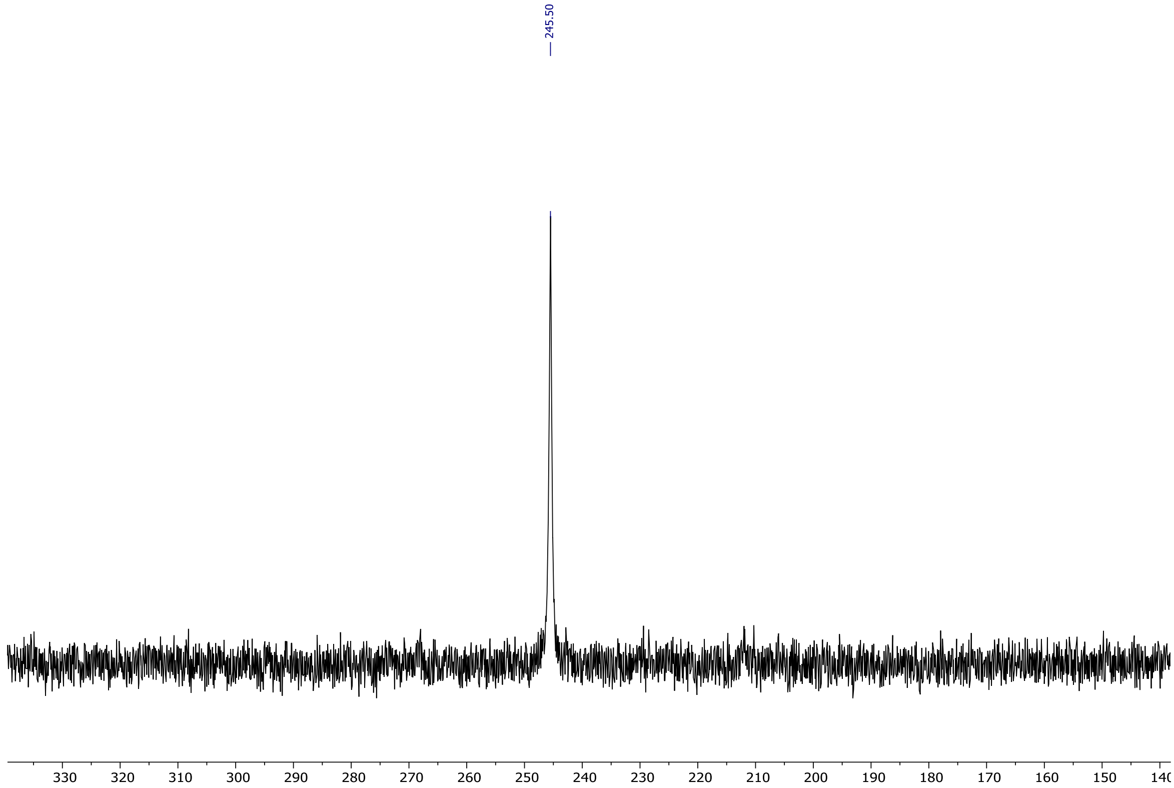

Figure S10. $^{31}P\{^1H\}$ NMR (202 MHz) spectrum of **5** in d_6-DMSO.

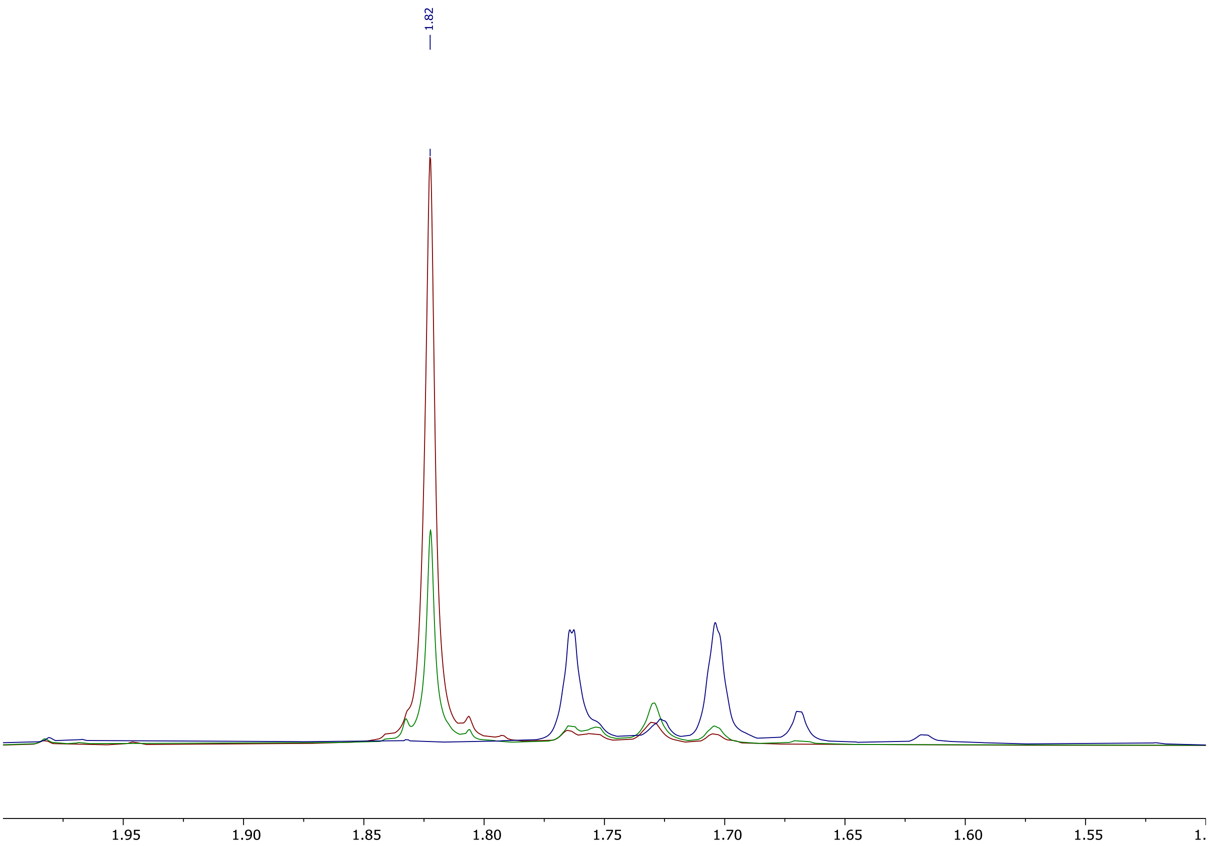

Figure S11. ^1H NMR spectrum of **5** in d_6-DMSO after 2 h (red), 6 h (green), and 16 h (blue) displaying loss of signal associated with **5** over time.

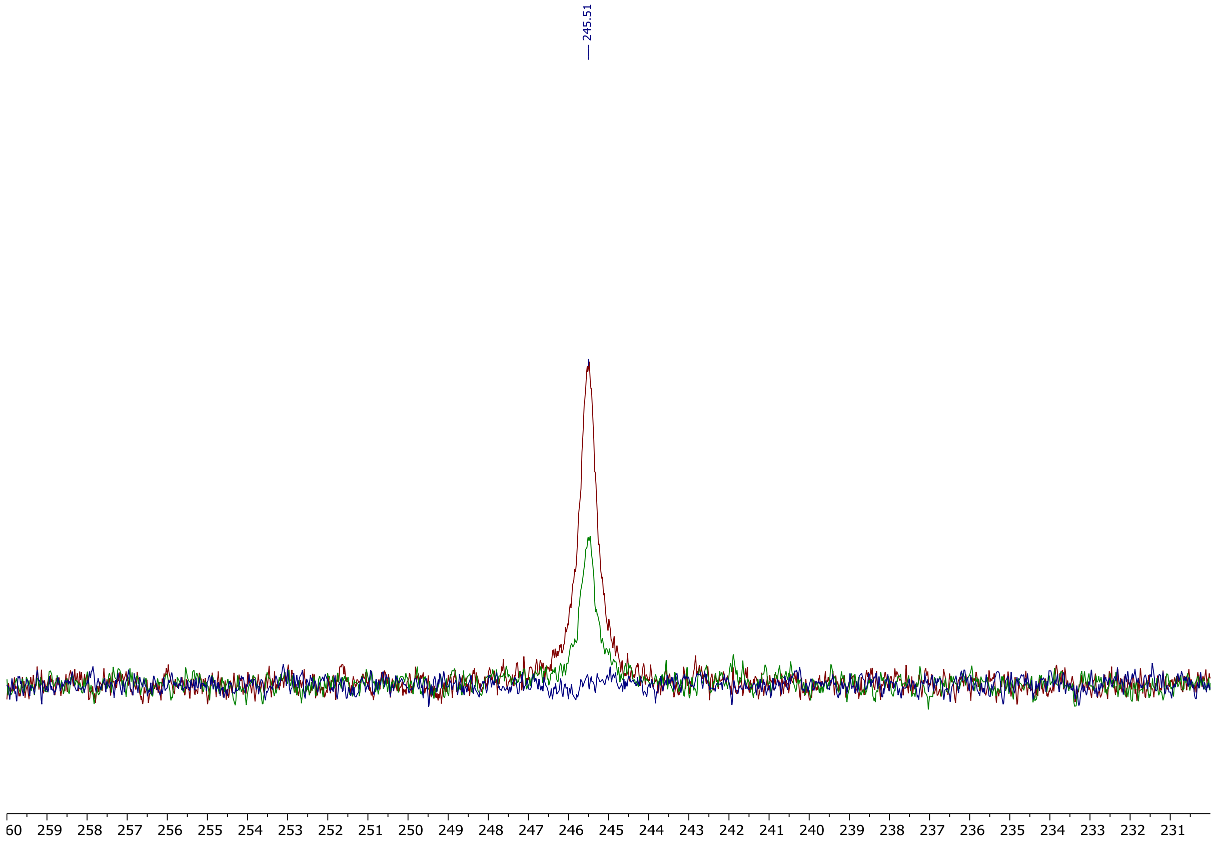

Figure S12. $^{31}P\{^1H\}$ NMR spectrum of **5** in d_6-DMSO after 2 h (red), 6 h (green), and 16 h (blue) displaying loss of signal associated with **5** over time.

2. Single crystal X-ray diffraction data

Single-crystal X-ray diffraction data were collected using an Oxford Diffraction Supernova dual-source diffractometer equipped with a 135 mm Atlas CCD area detector. Crystals were selected under Paratone-N oil, mounted on micromount loops and quench-cooled using an Oxford Cryosystems open flow N2 cooling device. Data were collected at 150 K using mirror monochromated Cu Kα (λ = 1.54184 Å) radiation and processed using the CrysAlisPro package, including unit cell parameter refinement and inter-frame scaling (which was carried out using SCALE3 ABSPACK within CrysAlisPro).[5] Structures were subsequently solved using direct methods.[6]

Table S1. Selected X-ray data collection/refinement parameters for **1, 3**·tol and **5**.

	**1**	**3·tol**	**5**
Formula	$C_{96}H_{132}Au_2N_4P_2Sm_2$	$C_{73}H_{94}N_4Ni_2P_2$	$C_{63}H_{90}P_3Sc_3$
CCDC	2219150	2219151	2219152
Fw [g mol^{-1}]	2098.62	1206.88	1075.13
Crystal system	monoclinic	monoclinic	monoclinic
Space group	$P2_1/n$	$P2_1/n$	$C2/c$
a (Å)	13.3207(2)	10.8597(2)	20.7447(6)
b (Å)	22.0522(3)	30.8769(5)	14.3514(3)
c (Å)	16.0911(2)	19.7884(4)	20.3469(5)
α (°)	90	90	90
β (°)	96.152(1)	94.019(2)	105.036(3)
γ (°)	90	90	90
V (Å^3)	4699.55(11)	6619.0(2)	5850.2(3)
Z	2	4	4
Radiation, λ (Å)	Cu Kα, 1.54184	Mo Kα, 0.71073	Cu Kα, 1.54184
Temp (K)	150(2)	150(2)	150(2)
ρ_{calc} (g cm^{-3})	1.483	1.211	1.221
μ (mm^{-1})	15.617	0.661	3.956
Reflections collected	57858	38385	25739
Indep. reflections	9796	8775	5157
Parameters	515	746	355
R(int)	0.0663	0.0485	0.0451
R1/wR2,[a] I ≥ 2σI (%)	3.85/9.01	4.87/11.72	5.32/14.98
R1/wR2,[a] all data (%)	5.33/9.94	7.54/12.85	6.30/15.93
GOF	1.066	1.075	1.053

[a] $R1 = [\Sigma||F_o| - |F_c||]/\Sigma|F_o|$; $wR2 = \{[\Sigma w[(F_o)^2 - (F_c)^2]^2]/[\Sigma w(F_o^2)^2]\}^{1/2}$; $w = [\sigma^2(F_o)^2 + (AP)^2 + BP]^{-1}$, where $P = [(F_o)^2 + 2(F_c)^2]/3$ and the A and B values are 0.0384 and 11.77 for **1**, 0.0546 and 4.85 for **2**·tol, and 0.1146 and 1.77 for **5**.

3. Computational details

3.1. General computational methods

Density functional theory (DFT) calculations were performed using the ORCA 5.0.2 software package.[5–7] All methods were used as implemented. Geometries were optimized using the B97-D3 functional and the def2-SVP basis set.[8,9] Analytical frequency calculations were carried out to verify all geometries as either true minima (N_{imag} = 0) or saddle points (N_{imag} = 1). Single point calculations were performed using the ωB97X-D3 functional[10] and the Resolution of Identity approximation (RIJCOSX),[13,14] and corrected for relativistic effects using the zeroth order regular approximation (ZORA). The segmented all-electron relativistically contracted basis set SARC-ZORA-TZVP was used for Au, and the relativistically contracted triple-zeta basis set ZORA-def2-TZVP was used for all other atoms, along with the SARC/J auxiliary basis set.[11,12] Nucleus-Independent Chemical Shifts (NICS) were computed using the PBE0 functional and def2-TZVP basis set,[9,13] and using the Gauge-Independent Atomic Orbitals (GIAO) method. Natural Bond Order analysis and Natural Population Analysis were carried out using the NBO 7.0 program.[14] Quantum Theory of Atoms in Molecules (QTAIM) topology analysis was carried out using Multiwfn 3.8.[15]

3.2. Computed mechanisms

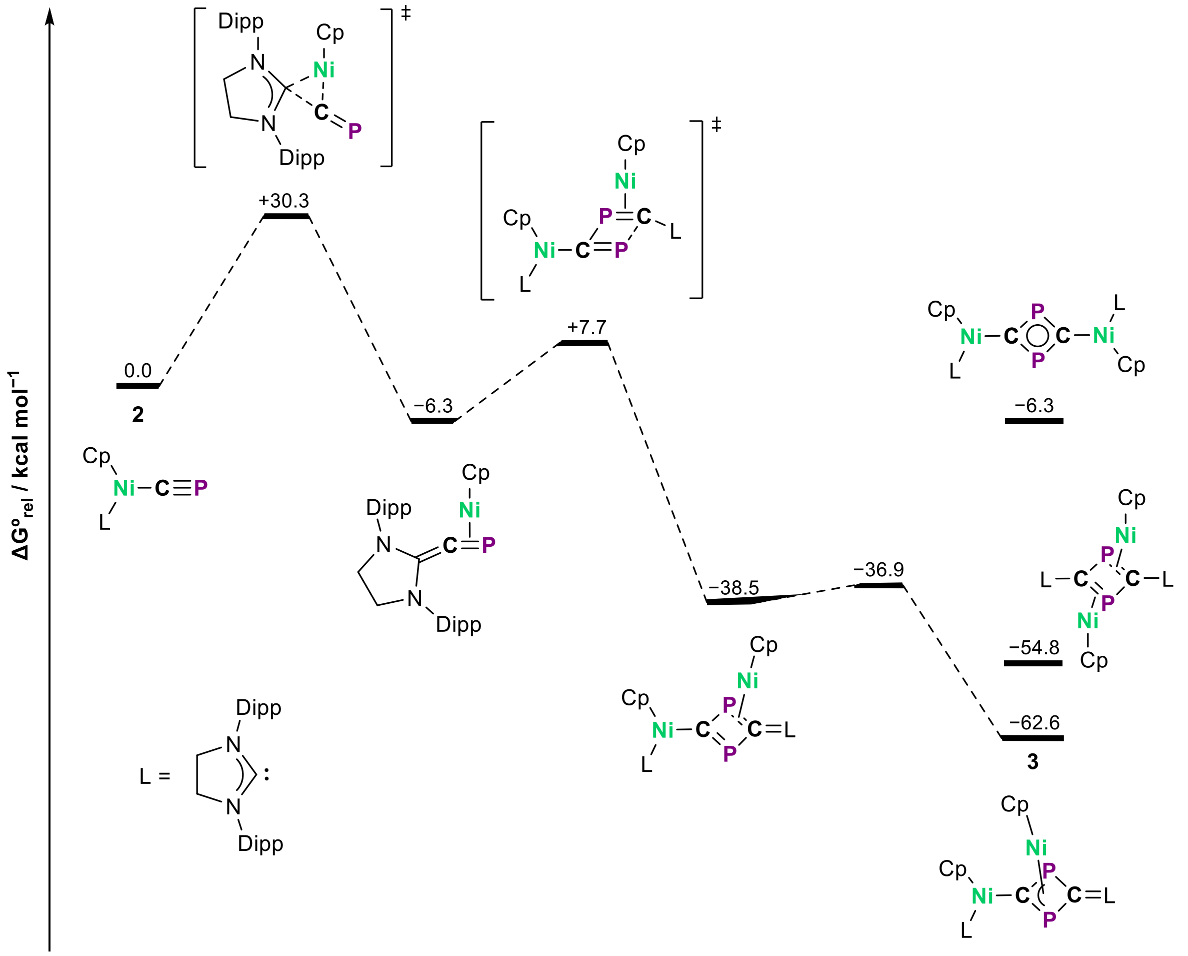

Figure S13. Computed mechanism for the dimerization of **2** to afford **3** (ZORA-ωB97X-D3/ZORA-def2-TZVP).

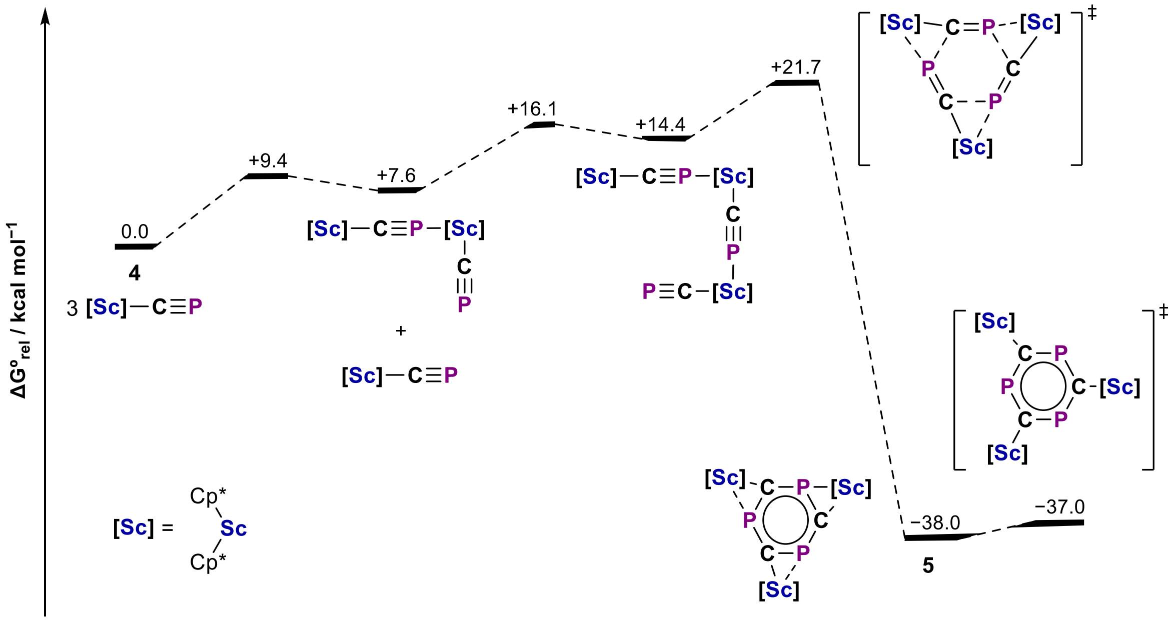

Figure S14. Computed mechanism for the trimerization of **4** to afford **5** (ZORA-ωB97X-D3/ZORA-def2-TZVP).

3.3. Electronic structure calculations

Table S2. Summary of NPA and NBO analyses of **B**, **2**, and **4**, as well as their M–C and C–P AIM delocalization indices.

	NBO Lewis structure	NPA charge			NLMO bond order		Delocalization Index	
		M	C	P	M−C	C−P	M−C	C−P
**B**	[Au]−C≡P:	+0.42	−0.98	+0.33	0.41	2.63	1.13	2.54
**2**	[Ni]−C≡P:	+0.72	−0.81	+0.24	0.52	2.67	1.01	2.42
**4**	⊕[Sc]←:C≡P: (⊖ on C)	+1.80	−1.09	+0.41	0.31	2.61	0.46	2.52

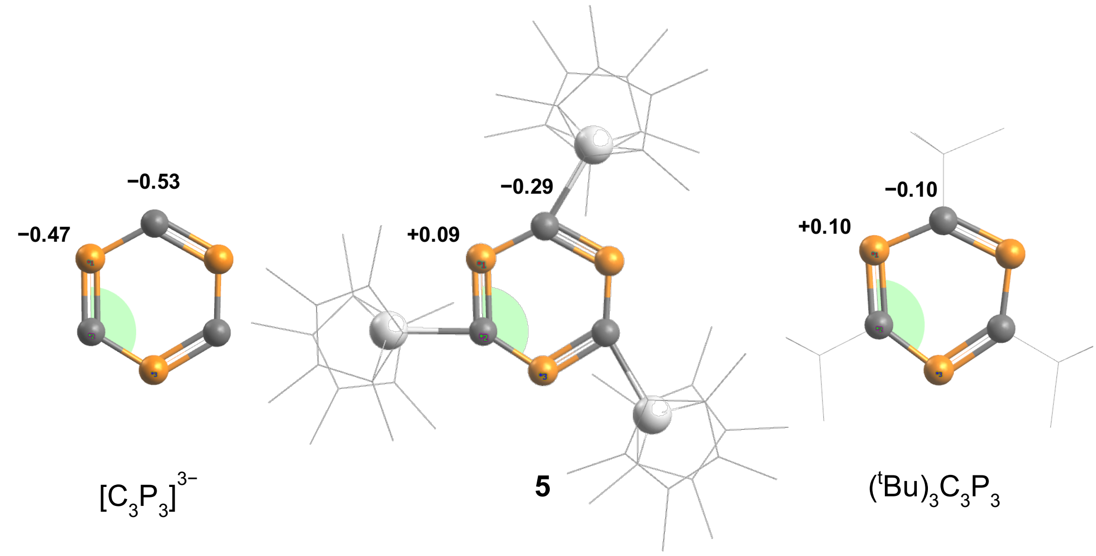

$[C_3P_3]^{3-}$ **5** $(^tBu)_3C_3P_3$

**Calcd:**	**122.1°**	**124.8°**	**130.4°**
XRD:		124.1(4)°	130.3(3)°

Figure S15. Calculated and measured P−C−P bond angles for $[C_3P_3]^{3-}$, **5**, and $(^tBu)_3C_3P_3$, as well as partial charges at C and P (Hirshfeld).

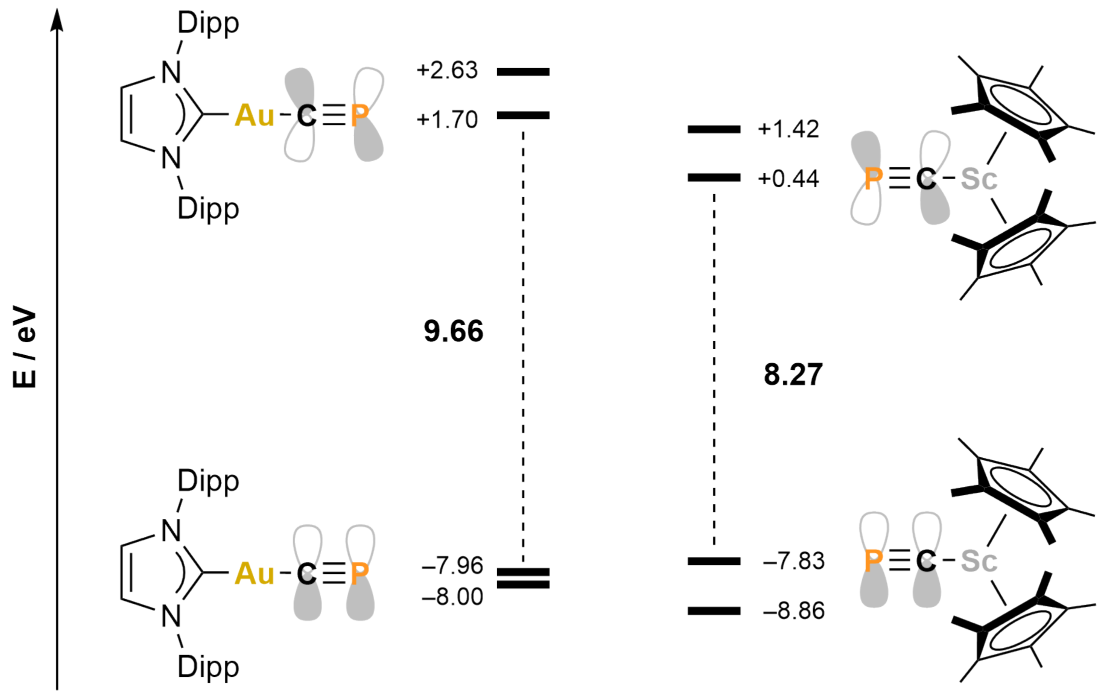

Figure S16. Energy level diagram comparing the C≡P π and π* orbitals in Au(IDipp)(CP) (**B**) with **4**.

3.4. Topological analysis

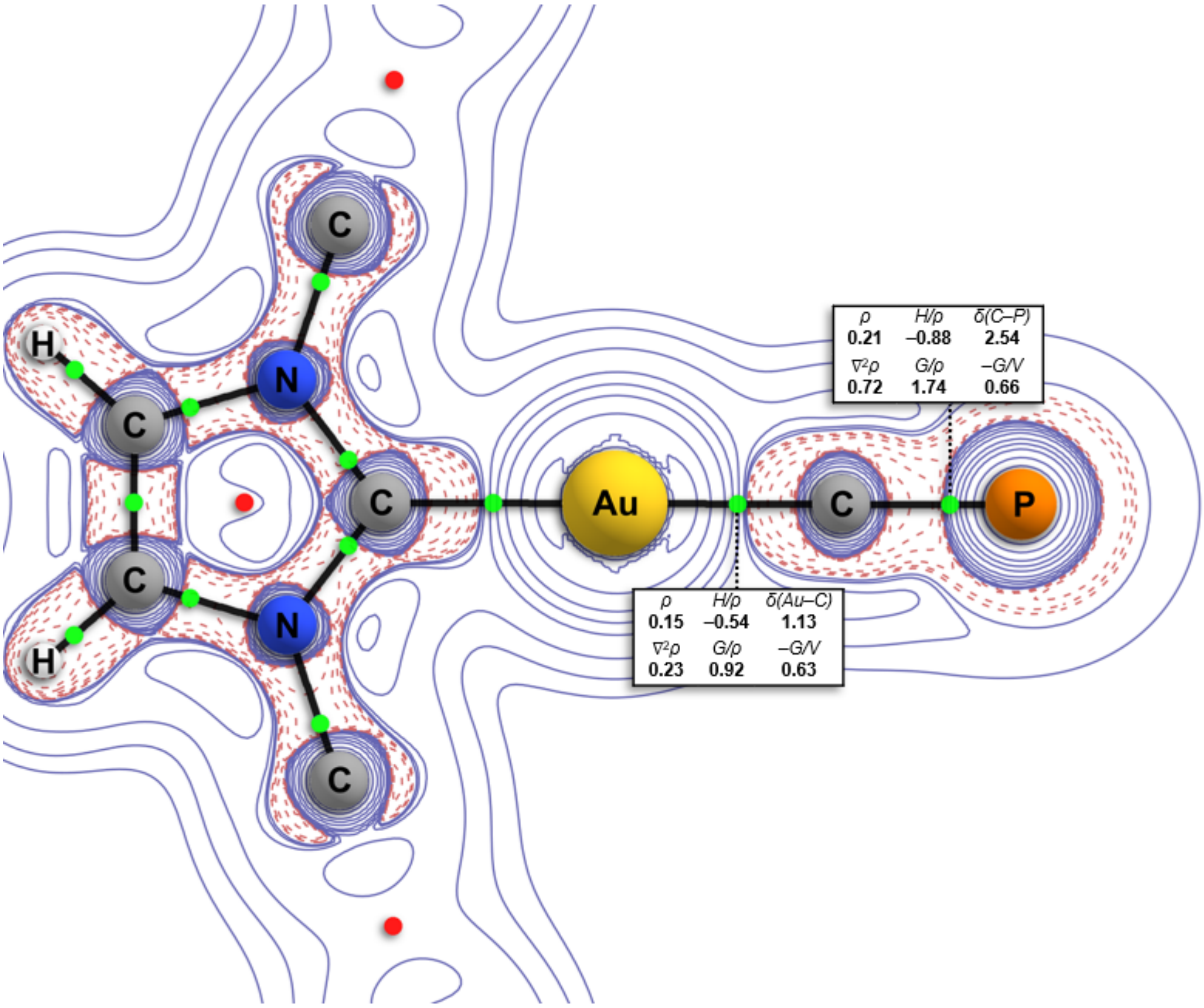

Figure S17. 2D Quantum Theory of Atoms in Molecules (QTAIM) analysis of **B**. Contour lines show the Laplacian of the electron density ($\nabla^2\rho$) (blue $\nabla^2\rho > 0$; red $\nabla^2\rho < 0$). Atomic critical points (3, +3) are displayed as atom labels. Bond critical points (3, −1) are displayed as green dots. Ring critical points (3, +1) are displayed as red dots. Bond paths are displayed as black lines. The values of the electron density (ρ), the Laplacian of the electron density ($\nabla^2\rho$), the relative energy density (H/ρ), the relative kinetic energy density (G/ρ), the ratio of kinetic and potential energy densities (−G/V), and the delocalization index (δ) at each bond critical point are given in atomic units (a.u.).

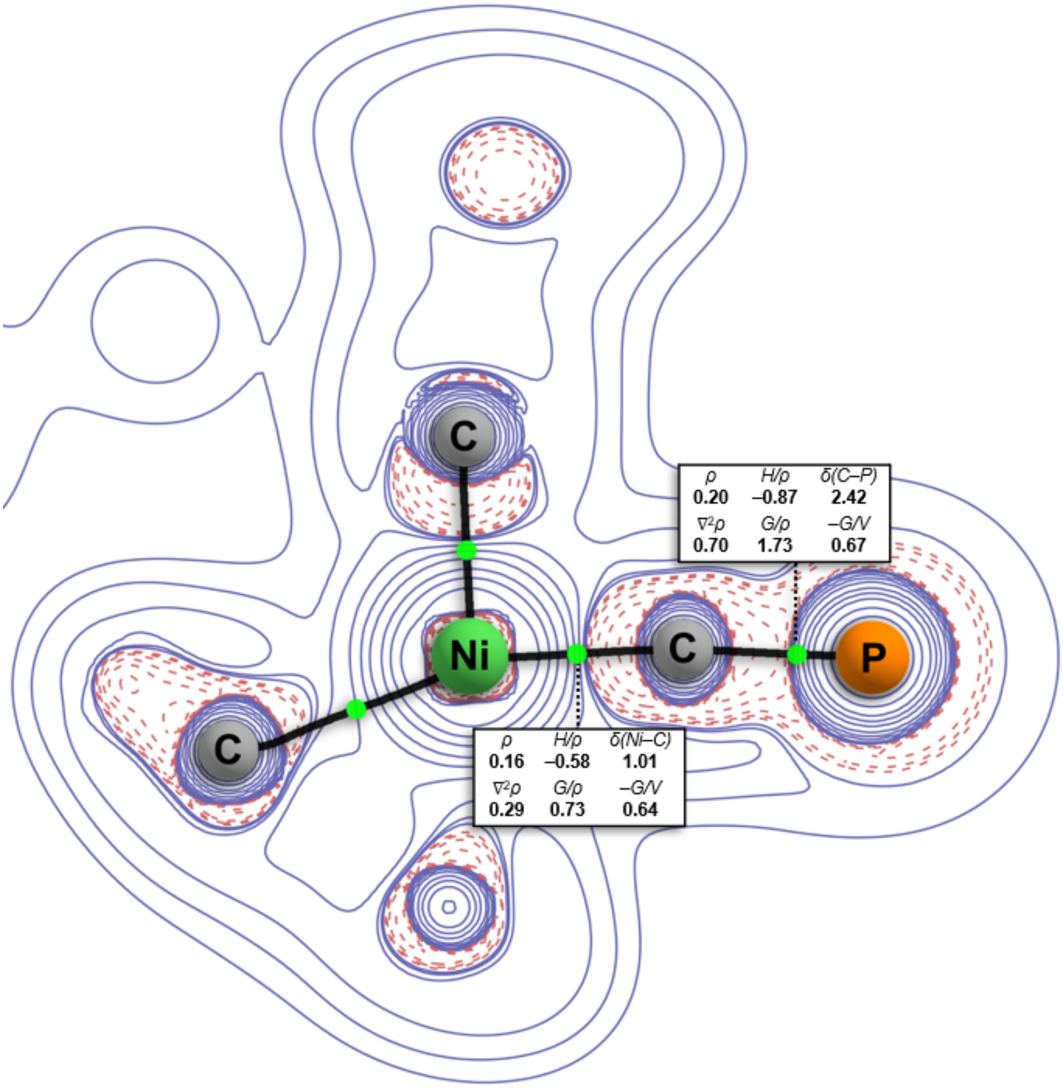

Figure S18. 2D Quantum Theory of Atoms in Molecules (QTAIM) analysis of **2**. Contour lines show the Laplacian of the electron density ($\nabla^2\rho$) (blue $\nabla^2\rho > 0$; red $\nabla^2\rho < 0$). Atomic critical points (3, +3) are displayed as atom labels. Bond critical points (3, −1) are displayed as green dots. Bond paths are displayed as black lines. The values of the electron density (ρ), the Laplacian of the electron density ($\nabla^2\rho$), the relative energy density (H/ρ), the relative kinetic energy density (G/ρ), the ratio of kinetic and potential energy densities (−G/V), and the delocalization index (δ) at each bond critical point are given in atomic units (a.u.).

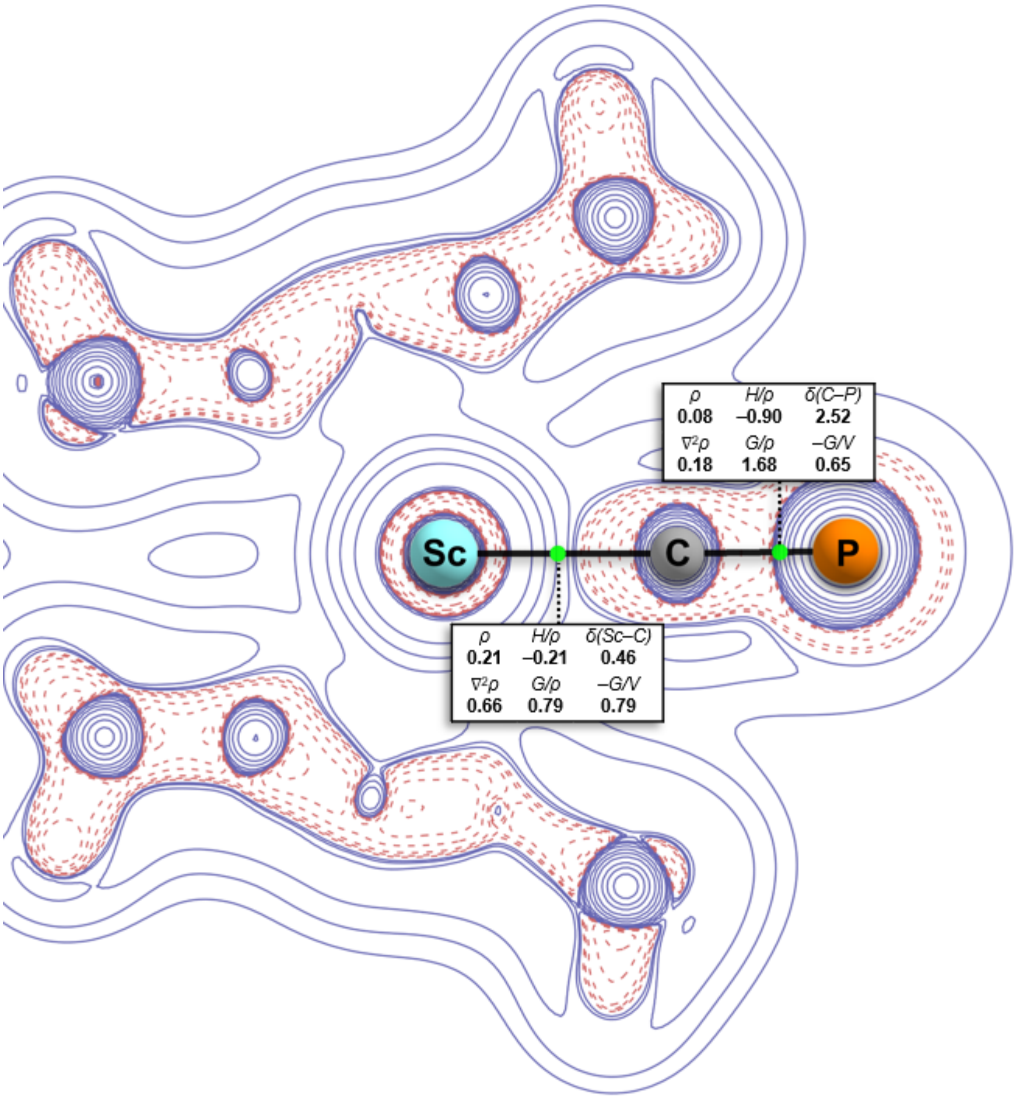

Figure S19. 2D Quantum Theory of Atoms in Molecules (QTAIM) analysis of **4**. Contour lines show the Laplacian of the electron density ($\nabla^2\rho$) (blue $\nabla^2\rho > 0$; red $\nabla^2\rho < 0$). Atomic critical points (3, +3) are displayed as atom labels. Bond critical points (3, −1) are displayed as green dots. Bond paths are displayed as black lines. The values of the electron density (ρ), the Laplacian of the electron density ($\nabla^2\rho$), the relative energy density (H/ρ), the relative kinetic energy density (G/ρ), the ratio of kinetic and potential energy densities (−G/V), and the delocalization index (δ) at each bond critical point are given in atomic units (a.u.).

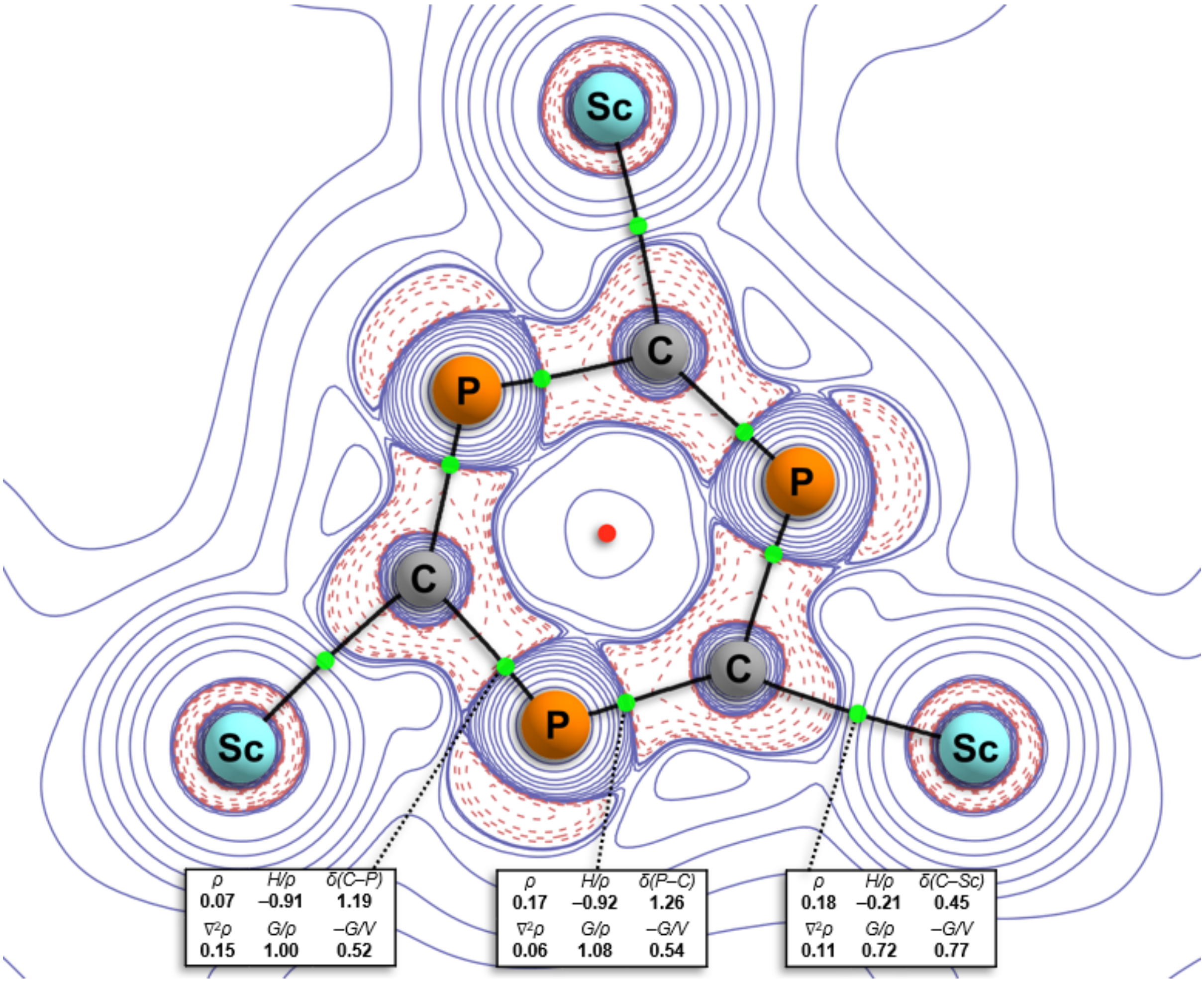

Figure S20. 2D Quantum Theory of Atoms in Molecules (QTAIM) analysis of **5**. Contour lines show the Laplacian of the electron density ($\nabla^2\rho$) (blue $\nabla^2\rho > 0$; red $\nabla^2\rho < 0$). Atomic critical points (3, +3) are displayed as atom labels. Bond critical points (3, −1) are displayed as green dots. Ring critical points (3, +1) are displayed as red dots. Bond paths are displayed as black lines. The values of the electron density (ρ), the Laplacian of the electron density ($\nabla^2\rho$), the relative energy density (H/ρ), the relative kinetic energy density (G/ρ), the ratio of kinetic and potential energy densities (−G/V), and the delocalization index (δ) at each bond critical point are given in atomic units (a.u.).

3.5. XYZ coordinates

Au(IDipp)(CP) (B)

Au	0.06531	-0.07596	-0.04735
C	0.02870	0.03166	-2.09734
P	0.12501	-0.27555	3.50688
N	1.09007	0.08849	-2.95572
C	0.67194	0.14497	-4.28246
C	-0.69700	0.12018	-4.25759
N	-1.06439	0.05320	-2.91652
C	0.09915	-0.18647	1.93183
H	1.38263	0.19370	-5.10585
H	-1.43818	0.14378	-5.05483
H	-1.45177	4.09742	-1.11812
H	-2.68098	4.47832	-3.37207
C	-2.01411	3.15472	-0.98881
H	-1.38400	2.44905	-0.41938
H	-1.46671	2.39050	-2.91650
C	-3.24773	3.54780	-3.18773
C	-2.40351	2.56430	-2.35890
H	-2.91250	3.37063	-0.38275
H	-4.17896	3.82999	-2.66430
H	-3.52757	3.11501	-4.16509
C	-3.09027	1.21538	-2.17617
C	-2.41854	-0.00159	-2.42886
H	-4.95463	2.05368	-1.45883
C	-4.40301	1.13331	-1.67885
C	-2.99356	-1.26906	-2.18913
C	-5.00774	-0.10748	-1.44384
C	-4.30975	-1.29457	-1.69364
H	-6.03034	-0.14896	-1.05051
H	-4.78830	-2.25848	-1.48827
H	-1.47146	-2.64645	-0.35660
C	-1.98356	-3.30170	-1.08292
H	-2.93579	-3.63392	-0.63125
H	-1.35511	-4.19595	-1.24567
C	-2.22276	-2.56461	-2.41413
H	-1.23109	-2.30014	-2.81883
C	-2.91388	-3.46315	-3.45567
H	-3.90808	-3.79730	-3.10741
H	-2.30809	-4.36718	-3.64892
H	-3.05414	-2.93139	-4.41417
H	3.42216	-2.85460	-4.57873
C	3.18996	-3.38178	-3.63582
H	2.60265	-4.28503	-3.88159
H	4.14476	-3.72117	-3.19531
H	1.44104	-2.24469	-3.15157
C	3.11929	-1.16233	-2.39263
C	2.40449	-2.48072	-2.66737
C	4.45059	-1.14349	-1.93992
C	5.08676	0.06346	-1.62522
H	4.99205	-2.08791	-1.81841
H	6.12336	0.05580	-1.26809
C	2.08115	-3.20303	-1.34452
H	1.50305	-4.12513	-1.53733
H	3.00674	-3.48443	-0.81045
H	1.48808	-2.55485	-0.67569
H	3.13583	3.21334	-4.22972
H	2.40198	4.56076	-3.30720
C	3.00370	3.63808	-3.21819
H	4.00150	3.92776	-2.84186
C	2.31199	2.63594	-2.27661
H	1.31489	2.42530	-2.69986
C	2.46215	0.08217	-2.51785
C	3.06977	1.31573	-2.19631
H	1.47821	4.14256	-0.92794
C	2.08936	3.22357	-0.87000
H	3.04929	3.48325	-0.38800
C	4.40293	1.27834	-1.74962
H	4.90686	2.21374	-1.48248
H	1.56631	2.50010	-0.22010

Ni(SIDipp)(Cp)(CP) (2)

Ni	0.00745	0.24522	1.95755
C	-0.32772	2.01126	1.89893
P	-0.50968	3.58578	1.96886
C	-0.05933	0.19664	0.10574
C	-0.83798	0.64558	-2.06545
C	0.65701	0.29485	-2.13883
C	0.32405	-1.76350	2.73862
H	0.21849	-2.67535	2.15062
C	1.56732	-1.06013	2.97574
H	2.53633	-1.30977	2.53987
C	1.26966	0.04996	3.78234
C	-0.15694	0.03003	4.07057
H	-0.68922	0.77571	4.66403
C	-0.71955	-1.13732	3.48611
H	-1.76596	-1.44427	3.52715
N	-1.17094	0.34341	-0.65831
N	1.02148	0.17796	-0.71323
H	1.96417	0.83011	4.09957
H	2.77911	2.57869	2.35137
H	-3.45922	3.32449	1.49263
H	-4.82072	3.64097	0.36591
H	4.20514	3.29811	1.52883
C	3.11329	3.13587	1.46111
C	-3.72030	3.57704	0.45229
H	2.62245	4.12389	1.48794
H	-3.31319	4.58106	0.23837
C	4.50267	0.63105	0.56326

C	-4.77807	0.78898	0.23594
H	5.15791	1.42913	0.92751
H	-5.52991	1.58277	0.29338
C	3.19268	0.94868	0.15981
C	-3.46477	1.11485	-0.15377
C	2.71600	2.39325	0.17694
C	-3.12794	2.55179	-0.52892
H	1.61523	2.38495	0.14846
H	-2.03054	2.65410	-0.47110
C	-3.59569	2.85895	-1.96755
H	-4.69858	2.81331	-2.03408
C	3.22108	3.13944	-1.07471
H	4.32563	3.19720	-1.08468
H	-3.28033	3.87350	-2.27354
H	2.82436	4.17088	-1.09473
H	-3.19625	2.13816	-2.70188
H	2.90866	2.63454	-2.00676
H	-2.23940	-3.30803	1.88903
H	1.76810	-3.56573	1.05524
H	2.96621	-4.33317	-0.01624
H	-3.02966	-4.18130	0.54663
C	1.98595	-3.82463	0.00717
C	-2.12317	-3.62012	0.83791
H	1.23131	-4.55621	-0.33371
H	-1.27575	-4.32631	0.78249
C	-4.17945	-1.54201	0.48764
C	4.15615	-1.70708	0.03504
H	-4.47302	-2.56684	0.73426
H	4.54018	-2.73198	-0.01097
C	-2.85939	-1.27546	0.08766
C	2.83575	-1.44419	-0.37085
C	-1.86390	-2.41985	-0.08438
C	1.96920	-2.58145	-0.89968
H	-0.87148	-2.02671	0.18459
H	0.93036	-2.21648	-0.92874
C	2.38385	-2.96584	-2.33518
H	3.40099	-3.39835	-2.34236
C	-1.80953	-2.87908	-1.55520
H	-2.79274	-3.26871	-1.87668
H	1.69147	-3.71787	-2.75629
H	-1.06288	-3.68326	-1.68888
H	2.39624	-2.09139	-3.00892
H	-1.54122	-2.05397	-2.23510
H	6.00713	-0.90574	0.82586
C	4.98263	-0.68122	0.50551
C	2.36560	-0.10690	-0.29755
H	-6.15985	-0.75121	0.87384
C	-5.13388	-0.52084	0.56254
C	-2.50930	0.07133	-0.21692
H	0.84398	-0.65750	-2.66651
H	1.25735	1.08109	-2.62471
H	-1.01310	1.71591	-2.27237
H	-1.46287	0.05447	-2.75437

{Ni(SIDipp)(Cp)}{Ni(Cp)}{μ_2-(SIDipp)C_2P_2} (3)

Ni	-0.02381	-0.02674	1.88111
Ni	-0.04539	1.85486	-0.78332
P	1.40155	0.11967	-1.10994
P	-1.24046	-0.01112	-1.35981
N	-2.97912	-0.07232	1.73823
N	-2.03857	-1.96016	2.36152
N	1.56302	-1.68110	-4.10145
N	-0.68440	-1.67773	-4.35577
C	-0.05492	0.07218	0.01508
C	0.22458	-0.50248	-2.37688
C	-1.77187	-0.66357	2.02133
C	-4.11502	-1.00441	1.95206
H	-4.82105	-0.97037	1.11272
H	-4.65983	-0.71678	2.87174
C	-3.42431	-2.35629	2.07238
H	-3.83438	-2.99569	2.86868
H	-3.45762	-2.92425	1.12445
C	-3.31568	1.32777	1.71988
C	-3.03043	2.14913	2.85014
C	-3.50727	3.47237	2.85483
H	-3.28532	4.11554	3.71144
C	-4.28719	3.97458	1.80959
H	-4.65683	5.00617	1.84199
C	-4.58832	3.15015	0.72573
H	-5.19748	3.53988	-0.09641
C	-4.11035	1.82830	0.64960
C	-2.33784	1.62337	4.10248
H	-1.65025	0.82368	3.79029
C	-1.48280	2.68024	4.81902
H	-0.84319	2.19728	5.57809
H	-0.83030	3.21411	4.10881
H	-2.10066	3.43134	5.34384
C	-3.37563	1.02659	5.07447
H	-2.88508	0.65382	5.99169
H	-4.11644	1.79178	5.37088
H	-3.92600	0.18481	4.62263
C	-4.48891	1.01332	-0.58221
H	-3.88672	0.08953	-0.56926
C	-4.13293	1.74876	-1.88544
H	-4.26647	1.07740	-2.74741
H	-4.77676	2.63359	-2.04154
H	-3.08273	2.07060	-1.87354
C	-5.98617	0.64128	-0.58965
H	-6.20606	-0.03511	-1.43586
H	-6.30958	0.14560	0.34269

H	-6.61330	1.54375	-0.70887
C	-1.11131	-2.94711	2.82943
C	-0.93536	-3.10705	4.23145
C	-0.08090	-4.12965	4.67719
H	0.07438	-4.27382	5.75096
C	0.59684	-4.95349	3.77007
H	1.27436	-5.73302	4.13861
C	0.40864	-4.78079	2.39693
H	0.94027	-5.43001	1.69241
C	-0.46413	-3.79352	1.90132
C	-1.63294	-2.19650	5.23649
H	-1.80222	-1.23694	4.72373
C	-0.78826	-1.91390	6.49171
H	-1.23447	-1.08281	7.06659
H	-0.75232	-2.78920	7.16534
H	0.24942	-1.64127	6.23963
C	-3.00739	-2.75424	5.65952
H	-3.49585	-2.07490	6.38127
H	-3.68913	-2.87592	4.80292
H	-2.89367	-3.74280	6.14101
C	-0.70128	-3.68842	0.40250
H	-1.38482	-2.84364	0.21914
C	0.59498	-3.38617	-0.35667
H	0.37762	-3.15525	-1.41284
H	1.10445	-2.50983	0.07123
H	1.28039	-4.24750	-0.32156
C	-1.35191	-4.98150	-0.12517
H	-1.52398	-4.91571	-1.21262
H	-0.69675	-5.85344	0.05207
H	-2.31697	-5.18847	0.37131
C	1.25839	1.17005	3.17937
H	0.97167	2.19608	3.40509
C	2.01625	0.72559	2.06184
H	2.42398	1.35654	1.27027
C	2.25693	-0.69922	2.21063
H	2.80870	-1.31886	1.49984
C	1.60319	-1.13403	3.36981
H	1.54804	-2.15930	3.73601
C	0.92618	0.01071	3.94234
H	0.34691	0.01037	4.86586
C	0.24428	3.61604	0.55843
H	0.47571	3.53484	1.62028
C	-1.05270	3.66984	-0.00713
H	-1.99289	3.66733	0.54049
C	-0.91159	3.72272	-1.44381
H	-1.73153	3.80016	-2.15924
C	0.47320	3.70486	-1.75833
H	0.91112	3.75186	-2.75369
C	1.19235	3.59134	-0.51644
H	2.27844	3.53273	-0.41283
C	0.36423	-1.21434	-3.52994
C	1.27927	-2.66256	-5.14678
H	1.30300	-3.70275	-4.76395
H	2.00956	-2.58182	-5.97003
C	-0.14401	-2.29133	-5.57055
H	-0.14046	-1.57731	-6.42149
H	-0.74022	-3.17095	-5.87005
C	2.88617	-1.35375	-3.67470
C	3.73246	-2.34070	-3.09692
C	5.05169	-1.98574	-2.75581
H	5.71348	-2.74170	-2.32048
C	5.52618	-0.68716	-2.93915
H	6.55182	-0.42736	-2.65112
C	4.68342	0.28160	-3.49273
H	5.06370	1.29605	-3.63998
C	3.37113	-0.02855	-3.88352
C	3.27958	-3.77316	-2.83823
H	2.17672	-3.77710	-2.85015
C	3.72557	-4.28803	-1.45648
H	3.23624	-5.25268	-1.23114
H	4.81554	-4.46304	-1.41992
H	3.46948	-3.57204	-0.65899
C	3.79184	-4.72584	-3.93938
H	3.40372	-5.75028	-3.78791
H	3.49943	-4.39231	-4.94873
H	4.89602	-4.77562	-3.91784
C	2.52001	1.01991	-4.58798
H	1.48216	0.88091	-4.24555
C	2.91324	2.46236	-4.24361
H	2.94373	2.61066	-3.15146
H	3.89757	2.73852	-4.66315
H	2.17608	3.16580	-4.66789
C	2.56383	0.79432	-6.11250
H	2.19629	-0.20895	-6.38378
H	1.93984	1.53909	-6.63739
H	3.59900	0.89036	-6.48826
C	-1.99373	-1.09709	-4.44685
C	-3.09693	-1.80504	-3.90359
C	-4.39810	-1.35510	-4.19026
H	-5.25831	-1.90714	-3.79693
C	-4.61272	-0.21238	-4.96662
H	-5.63403	0.12304	-5.18300
C	-3.51842	0.51985	-5.43533
H	-3.68972	1.43960	-6.00550
C	-2.19980	0.09768	-5.18752
C	-2.89706	-3.03387	-3.02862
H	-1.84467	-3.01658	-2.69661
C	-3.79269	-2.98356	-1.77880
H	-3.57654	-3.83532	-1.11502
H	-3.61693	-2.04733	-1.22202

H	-4.86542	-3.03613	-2.03681
C	-3.11742	-4.33041	-3.82938
H	-2.93607	-5.22118	-3.19994
H	-4.15426	-4.38810	-4.20932
H	-2.43915	-4.38237	-4.69893
C	-1.04307	0.95810	-5.67110
H	-0.11505	0.38988	-5.51648
C	-0.94209	2.22394	-4.80115
H	-0.09348	2.85311	-5.12260
H	-1.86219	2.83159	-4.87610
H	-0.79168	1.95717	-3.74326
C	-1.13753	1.29567	-7.16850
H	-0.23392	1.83785	-7.49937
H	-1.23494	0.38269	-7.78327
H	-2.00613	1.94216	-7.38853

$Sc(Cp^*)_2(CP)$ (4)

Sc	0.01297	0.09931	-0.08092
C	0.06682	0.64779	2.04876
P	0.10266	1.09104	3.57008
C	-2.32316	0.92037	0.17197
C	-2.35065	-0.50201	0.31371
C	-1.97881	-1.08253	-0.94418
C	-1.74053	-0.01520	-1.87101
C	-1.91239	1.21953	-1.16725
C	-2.76413	1.93829	1.18275
H	-3.79067	2.28850	0.95303
H	-2.10707	2.82509	1.18982
H	-2.75986	1.52778	2.20363
C	-2.74702	-1.26988	1.54299
H	-2.55089	-0.69221	2.46050
H	-2.19103	-2.22079	1.62840
H	-3.82450	-1.52923	1.52101
C	-1.96589	-2.55825	-1.23755
H	-1.43735	-3.13593	-0.45657
H	-1.47991	-2.78397	-2.20074
H	-2.99486	-2.96619	-1.28678
C	-1.58233	-0.14082	-3.35868
H	-1.31495	-1.16346	-3.66442
H	-0.81718	0.54096	-3.76567
H	-2.53595	0.11219	-3.86292
C	-1.81208	2.60265	-1.75139
H	-1.28463	2.60354	-2.71928
H	-1.27946	3.30005	-1.07774
H	-2.81598	3.03972	-1.92332
C	2.40888	-0.34045	0.42380
C	1.96000	-1.37476	-0.45929
C	1.67319	-0.78709	-1.73246
C	1.88443	0.62723	-1.61880
C	2.34048	0.89995	-0.28704
C	2.99579	-0.56404	1.78638
H	4.05132	-0.89168	1.69645
H	2.45242	-1.34310	2.34710
H	2.97094	0.34909	2.40016
C	1.90549	-2.84148	-0.12816
H	2.90847	-3.30947	-0.19163
H	1.24628	-3.39493	-0.81848
H	1.54017	-3.01838	0.90027
C	1.43508	-1.56340	-2.99575
H	1.09832	-0.92197	-3.82329
H	0.69025	-2.36751	-2.86862
H	2.37509	-2.05070	-3.32271
C	1.78983	1.64466	-2.72299
H	1.31091	2.58291	-2.38986
H	1.21242	1.26771	-3.58272
H	2.79504	1.91810	-3.10040
C	2.71940	2.24917	0.25594
H	2.19083	3.06506	-0.26818
H	3.80460	2.43996	0.13598
H	2.47953	2.33262	1.32947

$\{Sc(Cp^*)_2\}_3(\mu_3\text{-}C_3P_3)$ (5)

Sc	-0.00668	0.25598	-0.19813
C	-0.03408	-0.41880	1.93621
P	-0.02789	-1.79482	2.98316
C	-2.26809	1.32323	-0.35056
C	-2.47351	-0.00243	0.14110
C	-2.08711	-0.91806	-0.88955
C	-1.70424	-0.15868	-2.04060
C	-1.78751	1.22936	-1.69486
C	-2.63682	2.61115	0.32961
H	-3.54886	3.04997	-0.12345
H	-1.83853	3.37087	0.25029
H	-2.83730	2.46125	1.40081
C	-3.15013	-0.38446	1.41890
H	-2.89804	0.29533	2.24471
H	-2.85548	-1.39617	1.73420
H	-4.25210	-0.37814	1.29849
C	-2.12919	-2.41690	-0.76135
H	-1.57170	-2.76532	0.12855
H	-1.69401	-2.91611	-1.64311
H	-3.16655	-2.78879	-0.65112
C	-1.53057	-0.70371	-3.42817
H	-1.22843	-1.76207	-3.42637
H	-0.78826	-0.14542	-4.01971
H	-2.49113	-0.64114	-3.97779
C	-1.63335	2.40733	-2.61534
H	-1.20666	2.12215	-3.58861
H	-0.98836	3.19673	-2.18634
H	-2.61662	2.88030	-2.81127

C	2.48241	0.30466	0.20417
C	2.19707	-0.94824	-0.41968
C	1.74523	-0.69218	-1.75133
C	1.73969	0.72871	-1.94835
C	2.16987	1.34407	-0.72849
C	3.15517	0.46054	1.53167
H	4.22850	0.19341	1.45539
H	2.70670	-0.19384	2.29248
H	3.09968	1.49351	1.90470
C	2.43357	-2.29849	0.19109
H	3.49986	-2.43722	0.45551
H	2.15293	-3.11426	-0.49537
H	1.85119	-2.43303	1.12127
C	1.56281	-1.75217	-2.80324
H	1.21773	-1.33076	-3.75847
H	0.83978	-2.53133	-2.50280
H	2.52318	-2.26727	-3.00142
C	1.58557	1.44606	-3.25789
H	1.22949	2.48086	-3.12945
H	0.88858	0.93753	-3.94204
H	2.56246	1.50628	-3.77920
C	2.35658	2.82117	-0.51255
H	1.60551	3.41513	-1.06421
H	3.35251	3.16231	-0.86009
H	2.26746	3.09088	0.55230
P	-0.02850	0.18372	5.37315
C	-0.02350	-1.41053	4.70404
Sc	0.00092	-3.59912	5.17584
C	-2.25243	-4.27253	4.30869
C	-2.46886	-3.18350	5.20824
C	-2.09204	-3.61564	6.52011
C	-1.70715	-4.99211	6.44302
C	-1.77856	-5.38889	5.06837
C	-2.60680	-4.32887	2.84932
H	-3.52029	-4.93663	2.68931
H	-1.80482	-4.78429	2.24086
H	-2.79656	-3.32630	2.43823
C	-3.13969	-1.88562	4.88777
H	-2.85499	-1.50499	3.89718
H	-2.86971	-1.11079	5.62013
H	-4.24244	-1.99595	4.90579
C	-2.14553	-2.75742	7.75474
H	-1.68969	-1.76637	7.57592
H	-1.61021	-3.22110	8.60044
H	-3.18727	-2.57647	8.08613
C	-1.54871	-5.91504	7.61576
H	-1.22182	-5.38685	8.52451
H	-0.83440	-6.73286	7.42998
H	-2.52228	-6.38993	7.85195
C	-1.62453	-6.77620	4.51165
H	-1.19509	-7.47440	5.24545
H	-0.98285	-6.80179	3.61111
H	-2.60866	-7.18429	4.20494
C	2.49133	-3.28161	4.94880
C	2.20032	-3.19624	6.34478
C	1.74278	-4.47661	6.78562
C	1.73775	-5.35549	5.65234
C	2.17571	-4.60738	4.51232
C	3.16513	-2.20882	4.15213
H	4.24379	-2.15769	4.40338
H	2.73114	-1.22012	4.35781
H	3.08726	-2.38806	3.07012
C	2.43482	-1.99322	7.21068
H	3.50336	-1.70246	7.21139
H	2.14010	-2.17655	8.25710
H	1.86309	-1.11747	6.85261
C	1.55502	-4.86014	8.22775
H	1.20446	-5.89683	8.33790
H	0.83685	-4.20755	8.75569
H	2.51579	-4.78136	8.77350
C	1.57755	-6.84743	5.68089
H	1.24233	-7.24850	4.71103
H	0.86000	-7.18258	6.44577
H	2.54742	-7.33282	5.91195
C	2.36523	-5.16238	3.12671
H	1.56457	-5.87458	2.85487
H	3.32458	-5.71018	3.03473
H	2.36459	-4.36505	2.36663
P	-0.04094	1.26394	2.46302
C	-0.02752	1.48106	4.17871
Sc	0.00089	2.99102	5.83085
C	-2.26648	2.57245	6.82477
C	-2.46820	2.82121	5.43215
C	-2.08137	4.17411	5.16705
C	-1.70356	4.78089	6.40711
C	-1.78915	3.77896	7.42751
C	-2.63924	1.33326	7.58804
H	-3.56685	1.49540	8.17361
H	-1.85376	1.03023	8.30324
H	-2.81513	0.48036	6.91618
C	-3.14245	1.91107	4.45556
H	-2.88369	0.85583	4.62183
H	-2.85293	2.15550	3.42312
H	-4.24468	2.00564	4.52632
C	-2.12124	4.83086	3.81381
H	-1.64042	4.20150	3.04208
H	-1.60234	5.80412	3.81855
H	-3.16046	5.01398	3.47615
C	-1.53726	6.25460	6.63819
H	-1.20586	6.78524	5.73256

H	-0.82082	6.48556	7.44210
H	-2.50786	6.69944	6.93657
C	-1.63873	3.97429	8.91003
H	-1.21708	4.95968	9.15848
H	-0.99074	3.20725	9.37390
H	-2.62230	3.89897	9.41571
C	2.49101	2.62291	5.68226
C	2.20620	3.79018	4.90988
C	1.74993	4.81371	5.79787
C	1.73999	4.27118	7.12548
C	2.17206	2.90828	7.04797
C	3.16738	1.39572	5.15662
H	4.24645	1.58891	4.99102
H	2.73892	1.07471	4.19671
H	3.08734	0.55166	5.85673
C	2.44044	3.93743	3.43468
H	3.50484	3.77325	3.17763
H	2.16404	4.94232	3.07492
H	1.85231	3.20264	2.85420
C	1.56941	6.25626	5.41203
H	1.18963	6.86220	6.24738
H	0.87617	6.38958	4.56223
H	2.53820	6.69643	5.10360
C	1.57397	5.04094	8.40322
H	1.20159	4.40967	9.22582
H	0.88377	5.89207	8.29615
H	2.54899	5.45450	8.73186
C	2.35213	1.98340	8.22058
H	1.54670	2.10287	8.96858
H	3.30833	2.17438	8.74803
H	2.35135	0.92729	7.90721

TS1 (Ni)

Ni	-0.07916	-0.11939	-2.22852
P	-1.50308	-1.88499	-1.54710
N	1.67944	-0.86407	-4.37969
N	-0.43851	-0.99216	-4.97878
C	-0.16790	-2.15625	-2.45056
C	-0.47304	0.80895	-0.24229
H	-1.23631	0.45703	0.45483
C	-0.69326	1.69392	-1.34078
H	-1.64758	2.12454	-1.64236
C	0.56833	1.86334	-1.98774
H	0.75904	2.50853	-2.84497
C	1.59353	1.20204	-1.17943
H	2.66052	1.18229	-1.40430
C	0.95375	0.56846	-0.11486
H	1.41986	-0.07669	0.63310
C	0.39685	-0.72859	-3.92137
C	1.70352	-1.66974	-5.60862
H	1.82777	-2.74146	-5.35178
H	2.52993	-1.36934	-6.26994
C	0.31632	-1.38798	-6.18444
H	0.32687	-0.55764	-6.91788
H	-0.12974	-2.26755	-6.66906
C	2.87929	-0.40450	-3.75498
C	3.58897	-1.25137	-2.87288
C	4.79270	-0.77259	-2.32076
H	5.35740	-1.41041	-1.63143
C	5.27682	0.49975	-2.63875
H	6.21625	0.85552	-2.19856
C	4.56198	1.32430	-3.51793
H	4.94976	2.32013	-3.75531
C	3.35267	0.89424	-4.09075
C	3.09076	-2.64900	-2.53522
H	2.07441	-2.75334	-2.95274
C	2.93419	-2.85280	-1.01922
H	2.49908	-3.84725	-0.81462
H	3.90140	-2.78968	-0.48660
H	2.24866	-2.09713	-0.60180
C	3.99989	-3.72069	-3.16528
H	3.59316	-4.73140	-2.97796
H	4.08713	-3.58416	-4.25877
H	5.02237	-3.68530	-2.74421
C	2.58075	1.79861	-5.04855
H	1.51974	1.50673	-4.97794
C	2.68015	3.29276	-4.69437
H	2.49314	3.47738	-3.62325
H	3.67698	3.70449	-4.93476
H	1.94174	3.87037	-5.27894
C	3.02439	1.58291	-6.51010
H	2.90230	0.53587	-6.82980
H	2.43150	2.21665	-7.19468
H	4.09055	1.84766	-6.63352
C	-1.86283	-0.86767	-4.99286
C	-2.66126	-2.04088	-5.04279
C	-4.05771	-1.89095	-5.12690
H	-4.69100	-2.78381	-5.16948
C	-4.65004	-0.62604	-5.14322
H	-5.74102	-0.53044	-5.20156
C	-3.85012	0.52108	-5.08281
H	-4.32684	1.50611	-5.10027
C	-2.44936	0.42830	-5.01409
C	-2.05491	-3.44042	-5.04130
H	-0.99970	-3.33672	-4.73405
C	-2.71915	-4.37627	-4.01657
H	-2.16939	-5.33353	-3.96758
H	-2.71295	-3.92489	-3.01123
H	-3.76515	-4.60879	-4.28760
C	-2.12293	-4.07205	-6.44824

H	-1.59331	-5.04227	-6.46770
H	-3.17247	-4.25508	-6.74369
H	-1.67999	-3.42271	-7.22430
C	-1.58814	1.68783	-5.01819
H	-0.69904	1.45881	-4.41023
C	-2.27154	2.90586	-4.37843
H	-1.53366	3.71059	-4.21029
H	-3.06471	3.32106	-5.02661
H	-2.72988	2.65180	-3.40843
C	-1.11481	2.03894	-6.44249
H	-0.44587	2.91897	-6.42467
H	-0.56435	1.20696	-6.90951
H	-1.97687	2.27627	-7.09276

Int1 (Ni)

Ni	0.23563	0.62441	-1.46738
P	-1.53048	-0.19311	-2.23612
N	1.63307	-0.65267	-4.85212
N	-0.45992	-1.21110	-5.41468
C	-0.12998	-0.26182	-3.16194
C	0.75704	0.70880	0.63107
H	0.36489	0.00812	1.37194
C	0.12531	1.92808	0.21062
H	-0.81723	2.32509	0.59486
C	0.96796	2.55197	-0.76978
H	0.77349	3.49819	-1.27965
C	2.08491	1.69204	-0.98701
H	2.90572	1.86056	-1.68348
C	1.96590	0.56241	-0.11066
H	2.67521	-0.26326	-0.04350
C	0.32678	-0.68437	-4.39146
C	1.75182	-1.35717	-6.13010
H	2.10263	-2.39908	-5.97117
H	2.47133	-0.85440	-6.79922
C	0.30972	-1.31836	-6.65438
H	0.13730	-0.43675	-7.30694
H	0.03188	-2.22317	-7.22278
C	2.77300	-0.45739	-4.01679
C	3.14185	-1.44517	-3.07355
C	4.28812	-1.20668	-2.29186
H	4.59275	-1.94956	-1.54573
C	5.04689	-0.04407	-2.45837
H	5.93738	0.12079	-1.84021
C	4.66896	0.91691	-3.40677
H	5.26412	1.82918	-3.51608
C	3.52173	0.73304	-4.19617
C	2.35956	-2.74434	-2.90910
H	1.50967	-2.71833	-3.61039
C	1.75853	-2.88384	-1.49924
H	1.15276	-3.80561	-1.43074
H	2.54799	-2.94127	-0.72756
H	1.11203	-2.02122	-1.26829
C	3.22478	-3.96366	-3.28175
H	2.62516	-4.89144	-3.23716
H	3.63871	-3.86839	-4.30204
H	4.07577	-4.08196	-2.58643
C	3.02110	1.80790	-5.15658
H	2.48338	1.28953	-5.97025
C	1.98671	2.71057	-4.45151
H	1.19715	2.11380	-3.96621
H	2.47375	3.31543	-3.66562
H	1.51670	3.40213	-5.17492
C	4.14239	2.63684	-5.79813
H	4.90690	1.99299	-6.26897
H	3.72750	3.30441	-6.57463
H	4.65094	3.28112	-5.05776
C	-1.88219	-1.09390	-5.41978
C	-2.64625	-2.26789	-5.20451
C	-4.04626	-2.14833	-5.16583
H	-4.66289	-3.03618	-4.99139
C	-4.66403	-0.90337	-5.33690
H	-5.75736	-0.82675	-5.29850
C	-3.89248	0.24330	-5.55632
H	-4.38957	1.21066	-5.69184
C	-2.48853	0.17482	-5.60107
C	-1.95090	-3.60296	-4.95707
H	-0.97217	-3.55361	-5.46693
C	-1.65744	-3.79128	-3.45472
H	-1.09303	-4.72634	-3.28149
H	-1.06649	-2.95105	-3.05666
H	-2.59841	-3.84381	-2.87687
C	-2.71269	-4.80657	-5.53232
H	-2.10173	-5.72269	-5.44100
H	-3.65793	-4.99500	-4.99121
H	-2.95741	-4.66020	-6.59972
C	-1.66895	1.43688	-5.85503
H	-0.60450	1.18182	-5.73038
C	-1.97082	2.55303	-4.83943
H	-1.31893	3.42504	-5.02802
H	-3.01850	2.89752	-4.90825
H	-1.78450	2.21166	-3.80721
C	-1.86156	1.93054	-7.30290
H	-1.21226	2.80187	-7.50683
H	-1.61758	1.13942	-8.03488
H	-2.90760	2.23836	-7.48427

TS2 (Ni)

Ni	0.00369	-1.60153	1.79024
Ni	-0.30950	1.18443	-1.50774
P	0.63470	-0.70903	-1.47180

P	-1.49091	-2.65442	-1.04354
N	-2.77494	-2.02565	2.39939
N	-1.54223	-3.82886	2.70470
N	0.61383	-1.28021	-4.89408
N	-0.85274	0.41222	-5.18658
C	-0.66328	-1.87359	0.10909
C	-0.22190	-0.15385	-2.85798
C	-1.50506	-2.50420	2.40084
C	-3.77886	-3.08589	2.63200
H	-4.49478	-3.12520	1.79587
H	-4.34680	-2.87996	3.55682
C	-2.91786	-4.35950	2.71919
H	-3.10318	-4.94749	3.63204
H	-3.06291	-5.02306	1.84628
C	-3.15347	-0.63835	2.47060
C	-2.86387	0.09593	3.65827
C	-3.31045	1.42517	3.74610
H	-3.08884	2.00714	4.64613
C	-4.06323	2.00804	2.72158
H	-4.41153	3.04339	2.81425
C	-4.37047	1.26268	1.58338
H	-4.96507	1.71760	0.78438
C	-3.91317	-0.05985	1.42372
C	-2.19886	-0.54265	4.87319
H	-1.55753	-1.35692	4.50553
C	-1.29598	0.41317	5.66876
H	-0.71531	-0.15215	6.41843
H	-0.58525	0.93984	5.01330
H	-1.87963	1.17331	6.21837
C	-3.27141	-1.15078	5.80109
H	-2.80934	-1.62412	6.68566
H	-3.96323	-0.36556	6.15582
H	-3.87465	-1.91590	5.28550
C	-4.28801	-0.80641	0.15186
H	-3.71675	-1.75059	0.13867
C	-3.89070	-0.02926	-1.11258
H	-4.21485	-0.57383	-2.01082
H	-4.37085	0.96405	-1.15381
H	-2.80021	0.11447	-1.18297
C	-5.79251	-1.14553	0.13715
H	-6.03448	-1.78263	-0.73329
H	-6.10941	-1.67601	1.05376
H	-6.40477	-0.22805	0.06052
C	-0.43316	-4.64700	3.11958
C	-0.14934	-4.74335	4.51060
C	0.93143	-5.54513	4.91696
H	1.16912	-5.62639	5.98306
C	1.70455	-6.24508	3.98451
H	2.54748	-6.86065	4.32078
C	1.38961	-6.16646	2.62598
H	1.98520	-6.72828	1.89819
C	0.31668	-5.37861	2.16748
C	-1.01002	-4.06460	5.56996
H	-1.63657	-3.32011	5.05626
C	-0.18474	-3.32102	6.63450
H	-0.84952	-2.75382	7.30922
H	0.39440	-4.02097	7.26214
H	0.52769	-2.61483	6.17928
C	-1.95035	-5.08178	6.25080
H	-2.64603	-4.56790	6.93920
H	-2.54919	-5.64895	5.51699
H	-1.37122	-5.81757	6.83809
C	-0.00605	-5.35568	0.68496
H	-0.79544	-4.60358	0.52941
C	1.20007	-4.90951	-0.15579
H	0.91551	-4.80102	-1.21332
H	1.56525	-3.92822	0.18283
H	2.03155	-5.63633	-0.10156
C	-0.55871	-6.71343	0.21368
H	-0.85913	-6.65390	-0.84811
H	0.19796	-7.51475	0.30665
H	-1.44179	-7.01844	0.80492
C	1.08024	-0.00927	2.88022
H	0.58165	0.90106	3.21435
C	1.71046	-0.21010	1.63295
H	1.77729	0.51291	0.81961
C	2.24735	-1.55990	1.60002
H	2.78137	-1.99361	0.75214
C	1.93495	-2.19217	2.81832
H	2.16536	-3.21990	3.10206
C	1.14865	-1.25773	3.58731
H	0.74312	-1.44411	4.58119
C	-1.55946	2.50947	-0.29558
H	-2.42559	2.18036	0.27612
C	-1.55059	2.94482	-1.65291
H	-2.41789	3.00190	-2.31295
C	-0.19242	3.26357	-2.00606
H	0.15502	3.62548	-2.97450
C	0.63202	3.01932	-0.86437
H	1.71451	3.15735	-0.81158
C	-0.21157	2.52674	0.18442
H	0.10218	2.23937	1.18873
C	-0.17639	-0.34008	-4.21080
C	0.40785	-1.21507	-6.33852
H	-0.24193	-2.03426	-6.70742
H	1.37227	-1.28453	-6.87006
C	-0.25635	0.16259	-6.50464
H	0.50012	0.93553	-6.74996
H	-1.02782	0.17323	-7.28989
C	1.65006	-2.03981	-4.28797

C	1.49359	-3.44478	-4.17247
C	2.52272	-4.18501	-3.56312
H	2.42480	-5.27250	-3.47005
C	3.66299	-3.54991	-3.06040
H	4.44915	-4.13959	-2.57338
C	3.80426	-2.16155	-3.18223
H	4.70702	-1.67753	-2.79469
C	2.81295	-1.38122	-3.80134
C	0.26512	-4.14500	-4.74265
H	-0.47959	-3.35821	-4.94640
C	-0.38898	-5.13480	-3.76680
H	-1.30368	-5.56483	-4.21497
H	0.28122	-5.97710	-3.51689
H	-0.67527	-4.63065	-2.82876
C	0.60838	-4.83308	-6.07930
H	-0.29818	-5.25866	-6.54870
H	1.06716	-4.12680	-6.79298
H	1.32764	-5.65804	-5.92067
C	3.01756	0.11700	-4.01199
H	2.02165	0.57692	-4.10754
C	3.70540	0.81700	-2.82980
H	3.17217	0.60906	-1.88636
H	4.75943	0.50465	-2.71220
H	3.70123	1.91086	-2.98496
C	3.78199	0.36491	-5.32787
H	3.26055	-0.08951	-6.18858
H	3.88599	1.44840	-5.52341
H	4.79593	-0.07436	-5.28336
C	-2.21585	0.81599	-5.03296
C	-3.23027	-0.16152	-4.81564
C	-4.55359	0.27826	-4.64213
H	-5.34697	-0.45715	-4.47214
C	-4.88152	1.63746	-4.70549
H	-5.92013	1.95933	-4.56141
C	-3.88667	2.58129	-4.97843
H	-4.15678	3.63978	-5.05451
C	-2.54518	2.19374	-5.15177
C	-2.93368	-1.65663	-4.92239
H	-1.88320	-1.81128	-4.63450
C	-3.76536	-2.55486	-3.99606
H	-3.42287	-3.60119	-4.08778
H	-3.64651	-2.26187	-2.94224
H	-4.84118	-2.53882	-4.24996
C	-3.11342	-2.11433	-6.38617
H	-2.78820	-3.16390	-6.51252
H	-4.17700	-2.04838	-6.68129
H	-2.54091	-1.49007	-7.09175
C	-1.48685	3.22317	-5.53174
H	-0.52163	2.82263	-5.17086
C	-1.70499	4.61407	-4.91157
H	-0.81884	5.24922	-5.09061
H	-2.56739	5.13023	-5.37188
H	-1.87646	4.56863	-3.82572
C	-1.40738	3.37043	-7.06831
H	-0.59696	4.06660	-7.35407
H	-1.22681	2.41036	-7.57583
H	-2.35810	3.77717	-7.46033

Int2 (Ni)

Ni	0.03628	-0.46697	1.90095
Ni	0.44337	1.38897	-1.95559
P	1.38083	-0.37405	-1.04613
P	-1.28582	-1.04818	-1.16085
N	-2.90549	-0.14858	2.05162
N	-2.11047	-1.98150	2.95698
N	1.63138	-0.65800	-4.36739
N	-0.57383	-0.63304	-4.76631
C	-0.11675	-0.67712	0.05883
C	0.13498	-0.57505	-2.36560
C	-1.74649	-0.82959	2.31452
C	-4.09013	-0.84115	2.61838
H	-4.93488	-0.82176	1.92133
H	-4.40056	-0.33721	3.55322
C	-3.55427	-2.24037	2.85990
H	-3.94002	-2.71907	3.77177
H	-3.75250	-2.91280	2.00174
C	-3.08773	1.22032	1.64956
C	-2.62850	2.28049	2.48464
C	-2.92260	3.60148	2.10273
H	-2.56464	4.43067	2.71936
C	-3.67674	3.88196	0.96059
H	-3.88740	4.92073	0.68107
C	-4.16626	2.83156	0.18495
H	-4.77481	3.05240	-0.69820
C	-3.89083	1.49029	0.50750
C	-1.90375	2.03021	3.80345
H	-1.29928	1.12311	3.66668
C	-0.94543	3.16428	4.20161
H	-0.28043	2.82992	5.01729
H	-0.31716	3.48323	3.35365
H	-1.49062	4.05273	4.56914
C	-2.90129	1.77391	4.95196
H	-2.36158	1.59794	5.90051
H	-3.56683	2.64519	5.09381
H	-3.53613	0.89393	4.76366
C	-4.53401	0.39939	-0.34234
H	-4.06086	-0.56086	-0.07340
C	-4.30560	0.58819	-1.84665
H	-4.71738	-0.27119	-2.39784
H	-4.80613	1.49479	-2.23051

H	-3.23266	0.64236	-2.08367
C	-6.05381	0.32191	-0.06891
H	-6.49664	-0.55120	-0.58251
H	-6.29723	0.25250	1.00609
H	-6.56014	1.22705	-0.45094
C	-1.25947	-2.95515	3.56850
C	-1.00266	-2.85248	4.96237
C	-0.22990	-3.86047	5.56446
H	-0.01073	-3.80514	6.63567
C	0.27451	-4.93034	4.81419
H	0.88196	-5.70181	5.30252
C	0.00162	-5.01599	3.44577
H	0.39348	-5.85891	2.86585
C	-0.77875	-4.03864	2.80061
C	-1.54395	-1.69386	5.79405
H	-1.71138	-0.85532	5.09838
C	-0.56610	-1.21571	6.88245
H	-0.90834	-0.25204	7.30061
H	-0.50878	-1.93063	7.72315
H	0.45466	-1.07910	6.48971
C	-2.90130	-2.04048	6.43992
H	-3.29633	-1.17362	7.00063
H	-3.65791	-2.33024	5.69292
H	-2.79212	-2.88400	7.14618
C	-1.12090	-4.17789	1.32734
H	-1.58855	-3.23585	0.99628
C	0.12395	-4.35797	0.45397
H	-0.17659	-4.37280	-0.60349
H	0.82043	-3.51496	0.59217
H	0.65719	-5.30260	0.66834
C	-2.13288	-5.32036	1.10932
H	-2.44289	-5.36469	0.04989
H	-1.69319	-6.29921	1.37642
H	-3.03816	-5.18522	1.72833
C	1.41211	0.98741	2.92308
H	1.18486	2.04990	2.98293
C	2.12772	0.34485	1.88983
H	2.55442	0.82741	1.00866
C	2.23005	-1.06684	2.20602
H	2.72407	-1.81438	1.58174
C	1.57024	-1.29082	3.42896
H	1.45491	-2.24669	3.93928
C	1.00358	-0.03173	3.84397
H	0.43725	0.13883	4.75883
C	-0.40344	2.79060	-0.50278
H	-0.73613	2.45498	0.47887
C	-1.19552	2.88873	-1.66156
H	-2.26193	2.68158	-1.71687
C	-0.34100	3.29083	-2.76225
H	-0.66570	3.49158	-3.78411
C	0.97211	3.48541	-2.26358
H	1.82670	3.85250	-2.83033
C	0.96124	3.09700	-0.88045
H	1.81368	3.13352	-0.19780
C	0.37600	-0.60794	-3.75314
C	1.50659	-0.83432	-5.81508
H	1.69343	-1.88970	-6.10263
H	2.23024	-0.20433	-6.35833
C	0.05947	-0.41826	-6.07283
H	0.00962	0.64576	-6.37166
H	-0.43844	-1.02266	-6.84858
C	2.87208	-1.03076	-3.74525
C	3.16386	-2.40431	-3.54705
C	4.40991	-2.75810	-3.00089
H	4.63906	-3.81538	-2.82568
C	5.35591	-1.78290	-2.67847
H	6.32443	-2.07353	-2.25376
C	5.06485	-0.43361	-2.90059
H	5.81470	0.32202	-2.64834
C	3.82797	-0.02876	-3.43241
C	2.16533	-3.50287	-3.87169
H	1.28355	-3.03249	-4.32976
C	1.68314	-4.17826	-2.58119
H	0.92126	-4.94611	-2.80167
H	2.51311	-4.67221	-2.04501
H	1.24379	-3.42880	-1.90612
C	2.72396	-4.51920	-4.88332
H	1.95142	-5.26291	-5.15031
H	3.05887	-4.02238	-5.81177
H	3.58756	-5.07061	-4.47001
C	3.54556	1.44529	-3.68231
H	2.44670	1.56579	-3.63686
C	4.15558	2.34774	-2.60016
H	3.79660	2.05242	-1.60053
H	5.25980	2.31188	-2.60498
H	3.87326	3.39988	-2.77170
C	4.02870	1.87710	-5.08043
H	3.57555	1.26635	-5.87978
H	3.76637	2.93407	-5.27210
H	5.12682	1.77619	-5.16810
C	-1.98851	-0.81494	-4.66600
C	-2.50070	-2.08954	-4.29007
C	-3.88845	-2.29922	-4.35814
H	-4.29790	-3.27284	-4.07255
C	-4.75304	-1.30313	-4.82324
H	-5.83023	-1.49678	-4.89130
C	-4.23947	-0.05707	-5.18587
H	-4.92236	0.72667	-5.53085
C	-2.86379	0.22181	-5.09099
C	-1.57728	-3.25635	-3.95688

H	-0.67035	-2.84469	-3.48966
C	-2.17660	-4.25231	-2.95380
H	-1.41674	-4.99991	-2.66895
H	-2.50538	-3.73091	-2.03919
H	-3.03270	-4.80805	-3.37753
C	-1.16474	-3.96797	-5.26118
H	-0.48076	-4.80990	-5.05412
H	-2.05385	-4.36943	-5.78099
H	-0.65453	-3.27744	-5.95390
C	-2.36932	1.61135	-5.48053
H	-1.32481	1.69706	-5.13518
C	-3.16480	2.73153	-4.78598
H	-2.72762	3.71698	-5.02623
H	-4.21714	2.75087	-5.12111
H	-3.15647	2.61053	-3.69355
C	-2.41487	1.81043	-7.01061
H	-1.95872	2.77669	-7.29413
H	-1.88836	1.00785	-7.55360
H	-3.46120	1.81496	-7.36716

TS3 (Ni)

Ni	-0.16712	-0.47584	2.00239
Ni	0.49091	1.32260	-1.11131
P	1.34953	-0.76063	-0.85401
P	-1.24725	-0.12694	-1.25858
N	-3.11709	-0.19668	2.06703
N	-2.33150	-1.95317	3.12060
N	1.66513	-0.75417	-4.35847
N	-0.53021	-0.32999	-4.64956
C	-0.17394	-0.55954	0.13149
C	0.23706	-0.55628	-2.30514
C	-1.96063	-0.86742	2.36993
C	-4.28545	-0.77207	2.77616
H	-5.17825	-0.78729	2.14514
H	-4.51179	-0.16195	3.67145
C	-3.78830	-2.15695	3.13434
H	-4.13583	-2.51038	4.11538
H	-4.06245	-2.91620	2.37410
C	-3.30652	1.06336	1.39976
C	-2.78458	2.26562	1.95846
C	-3.08492	3.47780	1.31175
H	-2.69386	4.41420	1.71832
C	-3.87967	3.51838	0.16218
H	-4.08846	4.47638	-0.32843
C	-4.41884	2.33715	-0.34663
H	-5.06170	2.37535	-1.23137
C	-4.15844	1.09427	0.25779
C	-1.96069	2.27133	3.24264
H	-1.26865	1.41941	3.16184
C	-1.10292	3.53074	3.42982
H	-0.43335	3.39791	4.29776
H	-0.47657	3.73942	2.54707
H	-1.71731	4.42714	3.63333
C	-2.82759	2.04890	4.49719
H	-2.19934	2.08444	5.40615
H	-3.60547	2.82953	4.58932
H	-3.32925	1.06946	4.48761
C	-4.89522	-0.13322	-0.27838
H	-4.42600	-1.02857	0.16607
C	-4.81378	-0.30058	-1.80162
H	-5.30068	-1.24364	-2.10397
H	-5.33517	0.51448	-2.33176
H	-3.77285	-0.32454	-2.15268
C	-6.38756	-0.07582	0.13073
H	-6.89549	-1.02886	-0.10559
H	-6.53900	0.14330	1.20209
H	-6.90154	0.72570	-0.42987
C	-1.50969	-3.00781	3.62704
C	-1.33516	-3.10931	5.03264
C	-0.59676	-4.19636	5.53182
H	-0.44244	-4.29273	6.61213
C	-0.04253	-5.14802	4.66824
H	0.53925	-5.98411	5.07443
C	-0.23413	-5.03426	3.28746
H	0.19670	-5.78793	2.61935
C	-0.98024	-3.97485	2.73962
C	-1.91423	-2.08019	5.99785
H	-2.24361	-1.21825	5.39102
C	-0.87636	-1.56666	7.01494
H	-1.28158	-0.69918	7.56685
H	-0.62610	-2.34100	7.76231
H	0.06193	-1.25821	6.52736
C	-3.14043	-2.64166	6.74792
H	-3.59672	-1.86601	7.39010
H	-3.91797	-3.01668	6.06014
H	-2.84477	-3.48649	7.39656
C	-1.22086	-3.91394	1.23836
H	-1.63989	-2.92443	0.99742
C	0.08922	-4.03226	0.45117
H	-0.08554	-3.81335	-0.61219
H	0.82610	-3.30286	0.82014
H	0.52685	-5.04430	0.52106
C	-2.24154	-4.98256	0.80052
H	-2.44703	-4.89845	-0.28135
H	-1.85968	-6.00161	0.99645
H	-3.19893	-4.87740	1.34220
C	1.20279	0.81100	3.09649
H	1.02135	1.88402	3.07677
C	1.97534	0.07645	2.15868
H	2.49768	0.49551	1.29738

C	2.04764	-1.30524	2.59595
H	2.58287	-2.10269	2.07558
C	1.26158	-1.43387	3.74571
H	1.07281	-2.35336	4.29895
C	0.67472	-0.13466	4.02598
H	0.02547	0.10134	4.87071
C	1.35493	3.06485	-0.00490
H	1.73281	3.02776	1.01797
C	0.04416	3.37421	-0.40814
H	-0.80199	3.61238	0.23450
C	-0.02253	3.25425	-1.85548
H	-0.90691	3.45386	-2.45943
C	1.27055	2.92355	-2.34173
H	1.54523	2.76739	-3.38438
C	2.10037	2.70002	-1.19333
H	3.14556	2.38226	-1.20704
C	0.44190	-0.59266	-3.66526
C	1.41921	-0.83769	-5.79855
H	1.29245	-1.89120	-6.13028
H	2.24381	-0.39926	-6.37536
C	0.11101	-0.06462	-5.93889
H	0.30270	1.01980	-6.08879
H	-0.52143	-0.42485	-6.76636
C	2.95976	-0.93560	-3.78950
C	3.26872	-2.13435	-3.08099
C	4.53450	-2.25389	-2.48135
H	4.78081	-3.15696	-1.91620
C	5.49473	-1.24558	-2.60152
H	6.47451	-1.35876	-2.12206
C	5.20742	-0.10215	-3.35125
H	5.97309	0.67298	-3.46483
C	3.95066	0.07749	-3.95580
C	2.29463	-3.30909	-3.05376
H	1.27840	-2.89373	-2.94082
C	2.52942	-4.28213	-1.89141
H	1.71849	-5.02903	-1.85799
H	3.47707	-4.83862	-2.00975
H	2.55153	-3.75540	-0.92406
C	2.34751	-4.07961	-4.38920
H	1.60359	-4.89689	-4.39575
H	2.14141	-3.42730	-5.25142
H	3.34816	-4.52573	-4.53842
C	3.72591	1.30922	-4.82723
H	2.63470	1.42191	-4.95463
C	4.26502	2.61399	-4.21312
H	3.92838	2.75479	-3.17643
H	5.36965	2.63635	-4.21648
H	3.92150	3.48266	-4.80320
C	4.37187	1.10934	-6.21776
H	4.06310	0.16448	-6.69628
H	4.11344	1.94293	-6.89673
H	5.47266	1.07681	-6.12323
C	-1.91775	-0.62668	-4.61247
C	-2.37453	-1.96912	-4.52793
C	-3.74753	-2.22043	-4.69648
H	-4.11340	-3.25227	-4.63649
C	-4.64639	-1.18632	-4.98332
H	-5.70849	-1.40831	-5.14163
C	-4.18784	0.13319	-5.04227
H	-4.89901	0.94452	-5.23680
C	-2.83251	0.43916	-4.82948
C	-1.42003	-3.13358	-4.30852
H	-0.40114	-2.72362	-4.25614
C	-1.69361	-3.82101	-2.96093
H	-0.95369	-4.61888	-2.77000
H	-1.63634	-3.08881	-2.13774
H	-2.69851	-4.28225	-2.93942
C	-1.44891	-4.12816	-5.48214
H	-0.69719	-4.92450	-5.33339
H	-2.43622	-4.61406	-5.58588
H	-1.22353	-3.62004	-6.43730
C	-2.36328	1.88828	-4.82126
H	-1.30353	1.87234	-4.51231
C	-3.12635	2.71254	-3.76970
H	-2.75718	3.75449	-3.75059
H	-4.20952	2.75340	-3.98617
H	-2.99135	2.27954	-2.76556
C	-2.45406	2.53126	-6.21735
H	-2.03805	3.55562	-6.20560
H	-1.90011	1.94413	-6.97064
H	-3.50469	2.59898	-6.55631

{Ni(SIDipp)(Cp)}$_2$(η^1,η^1-C_2P_2)

Ni	0.42312	0.17011	-1.82650
C	0.49612	0.19982	0.00673
P	0.64413	-1.39730	1.07795
C	0.61408	-0.54268	-3.94084
H	0.24087	0.01231	-4.80161
C	1.93599	-0.44095	-3.38859
H	2.72512	0.23096	-3.72765
C	1.99377	-1.31984	-2.27960
H	2.84350	-1.47050	-1.61093
C	0.72361	-2.00623	-2.18035
H	0.46746	-2.74732	-1.42062
C	-0.11245	-1.54996	-3.22552
H	-1.13180	-1.87828	-3.42655
C	-0.15349	1.94301	-1.82859
N	0.69492	2.97113	-2.11244
N	-1.33127	2.48654	-1.40643
C	0.18926	4.26122	-1.61155

H	0.40324	5.07811	-2.31768
H	0.67760	4.49496	-0.64595
C	-1.30076	3.96827	-1.44030
H	-1.91029	4.33543	-2.28799
H	-1.70928	4.39344	-0.51410
C	1.95519	2.88370	-2.79375
C	1.95763	2.93642	-4.21528
C	3.15970	2.81433	-2.05496
C	3.19734	2.90884	-4.87666
H	3.22696	2.94381	-5.97036
C	4.37523	2.78530	-2.76475
H	5.31669	2.72362	-2.20755
C	4.39834	2.83218	-4.16068
H	5.35476	2.80842	-4.69684
C	0.66402	3.03651	-5.01637
H	-0.12640	2.57505	-4.40456
C	0.71310	2.28307	-6.35717
H	-0.30385	2.19627	-6.77979
H	1.32915	2.81584	-7.10402
H	1.12801	1.26766	-6.24596
C	0.27022	4.50701	-5.26485
H	-0.68216	4.56653	-5.82195
H	1.04845	5.01927	-5.85988
H	0.14670	5.07053	-4.32579
C	3.17196	2.77650	-0.53756
H	2.12817	2.69602	-0.19472
C	3.88254	1.52647	0.00338
H	3.78909	1.48803	1.10223
H	4.95818	1.51451	-0.25334
H	3.40617	0.61642	-0.39490
C	3.75824	4.07042	0.05480
H	3.66014	4.06203	1.15574
H	4.83109	4.18194	-0.19016
H	3.23490	4.96512	-0.32949
C	-2.59645	1.82401	-1.23231
C	-3.22557	1.20422	-2.35198
C	-3.26953	1.92017	0.01611
C	-4.50630	0.65200	-2.17706
H	-5.00166	0.16862	-3.02433
C	-4.55658	1.36129	0.12834
H	-5.07423	1.42106	1.09060
C	-5.17242	0.72752	-0.94991
H	-6.17315	0.29260	-0.83866
C	-2.60562	1.21369	-3.74590
H	-1.51412	1.16217	-3.61744
C	-3.01025	0.01062	-4.61203
H	-2.36387	-0.04594	-5.50560
H	-4.05301	0.09059	-4.96960
H	-2.91473	-0.93735	-4.05865
C	-2.94833	2.52376	-4.48423
H	-2.50974	2.52541	-5.49824
H	-4.04333	2.63656	-4.58527
H	-2.56849	3.41132	-3.95331
C	-2.69430	2.63320	1.23272
H	-1.63507	2.85314	1.01984
C	-2.69654	1.74326	2.48721
H	-2.14964	2.24363	3.30408
H	-3.71874	1.53665	2.84711
H	-2.19893	0.77873	2.30353
C	-3.44388	3.95232	1.50964
H	-2.95765	4.50499	2.33452
H	-4.49097	3.75835	1.80729
H	-3.47670	4.61322	0.62423
C	3.27400	-2.74332	2.95003
C	3.87851	-4.03202	2.36480
H	3.35536	-4.93115	2.73872
H	4.94797	-4.13637	2.62726
H	3.79793	-4.02183	1.26249
H	2.23494	-2.67083	2.59133
C	3.23595	-2.78318	4.46634
C	2.01944	-2.87622	5.18272
N	0.77371	-2.99057	4.47854
C	0.29837	-4.29269	3.97864
H	0.80035	-4.52127	3.01885
H	0.52210	-5.10190	4.69062
C	-1.19658	-4.03176	3.79087
H	-1.80881	-4.41602	4.62896
H	-1.58383	-4.46168	2.85734
N	-1.25923	-2.55138	3.76147
C	-2.53550	-1.91330	3.57967
C	-3.19049	-2.00965	2.32258
C	-2.57545	-2.68104	1.10263
C	-3.28260	-4.01321	0.78231
H	-3.30701	-4.69705	1.65046
H	-2.77051	-4.53091	-0.04965
H	-4.33112	-3.84218	0.47549
H	-1.51379	-2.87724	1.32853
C	-2.58237	-1.75827	-0.12718
H	-1.99774	-2.21129	-0.94492
H	-3.60307	-1.57780	-0.50449
H	-2.12537	-0.78259	0.09717
C	-4.49013	-1.48262	2.20165
H	-4.99593	-1.54333	1.23321
C	-5.13380	-0.87796	3.28038
C	-4.48238	-0.79673	4.51515
H	-4.99801	-0.33296	5.36137
H	-6.14418	-0.46766	3.16272
C	-3.19027	-1.31880	4.69821
C	-2.58263	-1.32461	6.09719
C	-2.91139	-2.64304	6.82683

H	-2.51483	-3.52253	6.29460
H	-2.48112	-2.64294	7.84432
H	-4.00541	-2.77203	6.91849
H	-1.49108	-1.25473	5.97745
C	-3.01256	-0.13139	6.96470
H	-4.05734	-0.22768	7.31208
H	-2.37532	-0.07029	7.86454
H	-2.92464	0.82015	6.41632
C	-0.09604	-1.98245	4.18687
Ni	0.43253	-0.19798	4.17645
C	0.51671	-0.23626	2.34263
P	0.50240	1.36359	1.27407
C	0.57959	0.53695	6.28854
H	0.21218	-0.02122	7.14987
C	1.90804	0.46264	5.74826
H	2.71062	-0.18611	6.10100
C	1.95417	1.33557	4.63351
H	2.80604	1.50224	3.97143
C	-0.16433	1.52309	5.56105
H	-1.19321	1.82751	5.75109
C	0.66923	1.99228	4.52052
H	0.40113	2.72122	3.75325
C	1.99800	-2.92761	6.60410
C	0.69393	-3.05732	7.38317
C	0.33618	-4.53672	7.63355
H	0.23654	-5.10622	6.69523
H	-0.61963	-4.61948	8.18163
H	1.12158	-5.02642	8.23805
H	-0.09814	-2.62069	6.75574
C	0.69966	-2.29485	8.71976
H	-0.32658	-2.23210	9.12373
H	1.31594	-2.80655	9.48100
H	1.08996	-1.26965	8.60893
C	3.22484	-2.87456	7.28771
H	3.23516	-2.90872	8.38178
C	4.43689	-2.77532	6.59372
H	5.38284	-2.73233	7.14698
C	4.43778	-2.73026	5.19756
H	5.38775	-2.65082	4.65735
C	3.98556	-1.48814	2.42287
H	5.05863	-1.47477	2.69014
H	3.90226	-1.44405	1.32341
H	3.50347	-0.58157	2.82239

{Ni(Cp)}$_2$(η^2,η^2-C_2P_2(SIDipp)$_2$)

Ni	1.10514	0.22443	-1.84332
P	-1.27210	1.61494	-0.26657
N	1.11574	-1.85376	0.65724
N	-1.02207	-1.93945	-0.08417
C	0.08511	0.30202	-0.10000
C	1.58111	1.01770	-3.80354
H	1.51691	2.07435	-4.04669
C	0.59310	0.04264	-4.07364
H	-0.37347	0.22240	-4.53811
C	1.09954	-1.22914	-3.63928
H	0.56836	-2.18190	-3.70136
C	2.42972	-1.04792	-3.16385
H	3.10003	-1.83802	-2.82614
C	2.72070	0.34955	-3.21798
H	3.66211	0.82592	-2.94370
C	0.05398	-1.07816	0.16933
C	0.98119	-3.17607	0.04707
H	1.44400	-3.95838	0.66638
H	1.46420	-3.17910	-0.95169
C	-0.54877	-3.33343	-0.09515
H	-0.82071	-3.83048	-1.04317
H	-0.97776	-3.92089	0.73330
C	2.10323	-1.50289	1.65430
C	1.77436	-1.60010	3.04209
C	2.73258	-1.20987	3.99571
H	2.48128	-1.25629	5.05836
C	4.00365	-0.77360	3.61764
H	4.72880	-0.46097	4.37847
C	4.34673	-0.76064	2.26705
H	5.35379	-0.45416	1.96986
C	3.42234	-1.13065	1.27306
C	0.46955	-2.21272	3.53523
H	-0.31695	-1.90024	2.83232
C	0.04986	-1.77258	4.94694
H	-0.95337	-2.16360	5.17621
H	0.73333	-2.17808	5.71457
H	0.01407	-0.67768	5.04952
C	0.55244	-3.75446	3.50424
H	-0.40710	-4.20210	3.82056
H	0.79481	-4.14503	2.50522
H	1.33728	-4.10665	4.19787
C	3.89528	-1.22153	-0.16424
H	3.00221	-1.10365	-0.79805
C	4.88251	-0.11714	-0.56654
H	4.45769	0.88260	-0.37901
H	5.84183	-0.19659	-0.02301
H	5.12019	-0.20098	-1.64056
C	4.50879	-2.61365	-0.41926
H	3.80557	-3.42700	-0.17284
H	4.80541	-2.72545	-1.47856
H	5.41287	-2.75901	0.20114
C	-2.41806	-1.55432	-0.06150
C	-3.22357	-1.82755	1.08682
C	-4.52187	-1.28949	1.15075
H	-5.13426	-1.48200	2.03661

C	-5.04318	-0.51714	0.11293
H	-6.04377	-0.07643	0.19677
C	-4.29181	-0.34422	-1.04794
H	-4.71930	0.22433	-1.87622
C	-2.99431	-0.87603	-1.17439
C	-2.80860	-2.78470	2.19814
H	-1.71217	-2.83414	2.20762
C	-3.25951	-2.35806	3.60501
H	-2.83403	-3.04311	4.35869
H	-2.93791	-1.33246	3.84591
H	-4.35678	-2.40850	3.71559
C	-3.34792	-4.19820	1.88355
H	-2.97349	-4.93536	2.61785
H	-4.45232	-4.20380	1.92617
H	-3.05932	-4.53676	0.87360
C	-2.28850	-0.79930	-2.51978
H	-1.22219	-0.59039	-2.32915
C	-2.82843	0.31526	-3.42394
H	-2.24887	0.36656	-4.35920
H	-3.87740	0.13409	-3.72083
H	-2.76554	1.29615	-2.92559
C	-2.38267	-2.16257	-3.23378
H	-1.86336	-2.12724	-4.20821
H	-1.92777	-2.96901	-2.63510
H	-3.43846	-2.43510	-3.41884
C	-1.33628	0.82886	3.00927
C	-0.22491	1.46957	3.61118
H	0.76352	1.03603	3.75064
C	-0.62647	2.81458	3.92682
H	-0.00600	3.57092	4.41079
Ni	-0.86221	2.40901	1.72014
C	0.17081	2.70945	-0.02464
P	1.53101	1.40942	0.00505
C	0.22379	4.11521	-0.03574
N	1.33637	4.88589	0.28433
C	2.70518	4.43724	0.32556
C	3.24553	3.92502	1.53611
C	4.58719	3.50648	1.54415
H	5.01298	3.08549	2.45839
C	5.38933	3.60854	0.40395
H	6.43165	3.26935	0.43279
C	4.85602	4.13903	-0.77064
H	5.48650	4.22218	-1.66248
C	2.40842	3.85205	2.80326
C	2.90813	2.79282	3.79325
H	2.99890	1.80634	3.31116
H	3.89075	3.06248	4.22243
H	2.20706	2.69930	4.63877
H	1.38911	3.56260	2.49454
C	2.32836	5.22934	3.48975
H	3.33677	5.58663	3.76972
H	1.72226	5.17023	4.41223
H	1.86824	5.98957	2.83832
C	3.51333	4.55656	-0.83557
C	2.99272	5.12263	-2.14815
C	3.26055	4.16621	-3.31968
H	2.91334	3.15451	-3.06886
H	4.33591	4.10760	-3.56433
H	2.73482	4.50515	-4.22985
H	1.90166	5.21901	-2.05566
C	3.58080	6.51928	-2.42909
H	4.67528	6.46048	-2.57066
H	3.14440	6.95165	-3.34817
H	3.39479	7.22025	-1.59629
C	1.01987	6.31736	0.19697
C	-0.51324	6.31344	0.17376
H	-0.91801	6.42822	1.19961
H	-0.94088	7.10120	-0.46595
H	1.44164	6.76596	-0.72120
H	1.42927	6.86521	1.06201
N	-0.81140	4.98479	-0.36338
C	-2.00008	4.65613	-1.09353
C	-1.88608	4.35308	-2.48257
C	-0.55845	4.47730	-3.22218
C	-0.51694	3.71293	-4.55180
H	-0.78771	2.65442	-4.41771
H	0.49891	3.75662	-4.97935
H	-1.19668	4.15687	-5.30115
H	0.22782	4.04877	-2.57687
C	-0.24185	5.96653	-3.47445
H	0.71950	6.08017	-4.00552
H	-1.03144	6.42067	-4.10032
H	-0.18430	6.54350	-2.53884
C	-3.05060	4.02530	-3.19522
H	-2.98242	3.77525	-4.25675
C	-4.30523	4.02671	-2.57851
H	-5.20138	3.76686	-3.15464
C	-4.41014	4.37007	-1.23174
H	-5.39614	4.38570	-0.75806
C	-3.27210	4.68141	-0.46282
C	-3.45829	5.06897	0.99781
C	-4.54200	4.22565	1.69094
H	-4.38096	3.15273	1.50650
H	-4.52938	4.39927	2.78042
H	-5.55386	4.49112	1.33577
H	-2.50284	4.86509	1.51272
C	-3.80489	6.56747	1.13073
H	-3.86622	6.86202	2.19480
H	-4.78573	6.77494	0.66435
H	-3.06524	7.21863	0.63717

C	-1.99877	2.98460	3.57549
H	-2.59457	3.88077	3.74805
H	-1.36932	-0.18599	2.61765
C	-2.43648	1.77014	2.96687
H	-3.43430	1.54076	2.58986

TS1 (Sc)

Sc	-0.05612	0.34930	-3.86946
C	-0.40486	-0.03795	-1.72121
P	-0.61779	-0.28552	-0.19646
C	-2.22524	-0.70746	-4.30428
C	-1.32040	-1.76243	-4.03578
C	-0.35123	-1.77694	-5.06750
C	-0.68187	-0.76417	-6.00008
C	-1.83255	-0.08884	-5.51800
C	-3.42743	-0.36050	-3.48469
H	-4.27743	-0.99702	-3.74634
H	-3.74692	0.67157	-3.63943
H	-3.23642	-0.48401	-2.41805
C	-1.44481	-2.78467	-2.95494
H	-1.97354	-2.39948	-2.08518
H	-0.47273	-3.13319	-2.60438
H	-1.99298	-3.65786	-3.32139
C	0.74791	-2.78227	-5.21779
H	1.11644	-3.13233	-4.25203
H	1.60258	-2.38284	-5.76644
H	0.40574	-3.66578	-5.76478
C	-0.07796	-0.63368	-7.36112
H	1.01011	-0.71459	-7.35355
H	-0.33458	0.30838	-7.84124
H	-0.44784	-1.43256	-8.01037
C	-2.59438	0.98560	-6.22905
H	-1.97634	1.51079	-6.95683
H	-2.99715	1.73443	-5.54471
H	-3.44400	0.56831	-6.77681
C	1.01819	2.35052	-2.90848
C	2.04300	1.41572	-3.18965
C	2.10509	1.24110	-4.59609
C	1.12230	2.07734	-5.18465
C	0.43165	2.73580	-4.13875
C	0.70098	2.95126	-1.57873
H	1.19064	3.92441	-1.47887
H	1.04010	2.32554	-0.75530
H	-0.36929	3.10856	-1.43860
C	2.94808	0.77912	-2.18417
H	3.82046	1.40871	-1.98914
H	3.32391	-0.18855	-2.52207
H	2.44247	0.61556	-1.23289
C	3.13247	0.43044	-5.32287
H	2.81061	0.16714	-6.33077
H	3.36997	-0.50064	-4.80455
H	4.07055	0.98363	-5.42198
C	1.00306	2.42163	-6.63313
H	-0.03053	2.55713	-6.95416
H	1.45156	1.66934	-7.27900
H	1.52026	3.36398	-6.83631
C	-0.63752	3.76988	-4.30425
H	-1.19174	3.64445	-5.23569
H	-0.21444	4.77866	-4.31803
H	-1.36192	3.74402	-3.48809
Sc	0.12965	-0.60481	3.79084
C	1.62000	0.70228	2.80966
P	2.68146	1.63221	2.14209
C	-1.40919	1.31908	3.57120
C	-0.60536	1.58596	4.70377
C	-0.89298	0.61057	5.68663
C	-1.89699	-0.24681	5.17276
C	-2.19572	0.17327	3.85160
C	-1.49056	2.18898	2.35972
H	-2.06414	3.09396	2.58023
H	-1.98332	1.68880	1.52624
H	-0.50209	2.49718	2.01753
C	0.26850	2.78111	4.89794
H	0.65924	3.15641	3.95407
H	1.12842	2.56212	5.53180
H	-0.29335	3.58914	5.37670
C	-0.36091	0.61410	7.08479
H	0.69657	0.88281	7.12545
H	-0.46673	-0.35776	7.56581
H	-0.89470	1.33967	7.70551
C	-2.69733	-1.21443	5.98017
H	-2.10762	-1.70806	6.75381
H	-3.15714	-1.98984	5.37020
H	-3.50903	-0.68769	6.49121
C	-3.24524	-0.41830	2.96125
H	-3.39386	-1.48228	3.15487
H	-2.99277	-0.31829	1.90436
H	-4.21274	0.07067	3.10780
C	1.61239	-2.35309	2.86516
C	2.01536	-2.11577	4.20417
C	0.97034	-2.54164	5.05953
C	-0.07895	-3.04491	4.25028
C	0.31138	-2.90993	2.89676
C	2.45879	-2.18719	1.64576
H	2.93013	-3.13755	1.37760
H	3.24763	-1.45447	1.80312
H	1.88227	-1.85044	0.78289
C	3.34355	-1.58909	4.64473
H	4.06296	-2.40239	4.77806
H	3.28017	-1.06251	5.59884

H	3.76042	-0.88795	3.92234
C	1.04243	-2.61232	6.55188
H	0.05272	-2.60361	7.00919
H	1.60537	-1.78264	6.98173
H	1.53595	-3.53299	6.87625
C	-1.25497	-3.84647	4.70474
H	-2.15228	-3.64805	4.11611
H	-1.50233	-3.67286	5.75000
H	-1.04086	-4.91469	4.60140
C	-0.45825	-3.45104	1.73273
H	-1.53128	-3.26761	1.82094
H	-0.32602	-4.53458	1.65353
H	-0.13438	-3.01633	0.78765

Int1 (Sc)

P	-0.09512	0.98958	4.46593
C	-0.08186	-0.37294	3.71384
Sc	-0.09611	-2.11948	2.34749
C	-2.16237	-1.10074	1.51224
C	-2.52067	-1.91212	2.61442
C	-2.25665	-3.26134	2.26957
C	-1.76719	-3.28693	0.94140
C	-1.68053	-1.94966	0.48611
C	-2.33108	0.37746	1.38902
H	-3.27012	0.62079	0.88588
H	-1.52921	0.83929	0.81218
H	-2.33417	0.87730	2.35443
C	-3.15501	-1.45477	3.88640
H	-3.01724	-0.38640	4.03295
H	-2.73943	-1.95145	4.76419
H	-4.22943	-1.65314	3.87714
C	-2.56735	-4.45106	3.12420
H	-2.45229	-4.23368	4.18697
H	-1.92393	-5.30147	2.89690
H	-3.59776	-4.78529	2.98085
C	-1.62664	-4.49824	0.07947
H	-1.52095	-5.41218	0.66021
H	-0.77725	-4.43846	-0.59948
H	-2.51919	-4.61692	-0.54003
C	-1.30883	-1.50893	-0.89481
H	-0.68282	-2.23916	-1.40617
H	-0.76972	-0.56045	-0.89324
H	-2.19948	-1.36235	-1.51060
C	2.18810	-2.25443	3.25593
C	1.62230	-3.54693	3.34500
C	1.42588	-4.03673	2.03222
C	1.83205	-3.02510	1.12585
C	2.29192	-1.92089	1.88478
C	2.74326	-1.46956	4.39509
H	3.73098	-1.84962	4.66824
H	2.11209	-1.51180	5.28166
H	2.85684	-0.41915	4.13794
C	1.37516	-4.31021	4.60853
H	2.24764	-4.90411	4.89245
H	0.53770	-5.00238	4.51229
H	1.15597	-3.64743	5.44602
C	1.08830	-5.45551	1.70757
H	0.83609	-5.59674	0.65935
H	0.25474	-5.83649	2.29930
H	1.94450	-6.10122	1.91783
C	1.92627	-3.14173	-0.36224
H	1.65541	-2.21646	-0.87119
H	1.28309	-3.92641	-0.75685
H	2.94661	-3.38520	-0.66750
C	2.83769	-0.63814	1.33943
H	2.57987	-0.50247	0.28921
H	3.92771	-0.61007	1.40479
H	2.45833	0.23031	1.87980
P	-0.89121	3.98585	1.81107
C	-0.57595	3.88113	3.33661
Sc	-0.11348	3.70819	5.50185
C	-2.08037	2.66214	6.64665
C	-2.58700	3.46052	5.59525
C	-2.27680	4.81100	5.89162
C	-1.60163	4.84516	7.13424
C	-1.46576	3.51182	7.59284
C	-2.33927	1.20721	6.87450
H	-3.13563	1.07463	7.61275
H	-1.46393	0.67520	7.24981
H	-2.65667	0.69807	5.96670
C	-3.44812	2.98273	4.47454
H	-3.13888	2.00426	4.10624
H	-3.42068	3.65821	3.62261
H	-4.48785	2.89602	4.80146
C	-2.66750	6.00150	5.07463
H	-2.65103	5.77998	4.00847
H	-1.99261	6.84331	5.23471
H	-3.67290	6.34704	5.32965
C	-1.33004	6.07710	7.93406
H	-1.10206	6.93968	7.31091
H	-0.50331	5.94990	8.63086
H	-2.20874	6.33967	8.52895
C	-1.01195	3.05784	8.94312
H	-0.51222	3.84736	9.49965
H	-0.32693	2.20897	8.89531
H	-1.86646	2.73585	9.54489
C	2.25702	3.51377	4.74775
C	1.93143	4.88672	4.68787
C	1.69287	5.34284	6.00221
C	1.88092	4.25046	6.88471

C	2.21341	3.11540	6.10807
C	2.70168	2.70135	3.57589
H	3.67902	3.03899	3.22140
H	2.00429	2.77433	2.73972
H	2.80157	1.64716	3.82738
C	2.00183	5.75810	3.47793
H	2.94667	6.30958	3.46039
H	1.19506	6.49057	3.44704
H	1.93518	5.17946	2.55948
C	1.52042	6.78407	6.36310
H	1.28726	6.92322	7.41626
H	0.73180	7.26858	5.78442
H	2.44071	7.33889	6.16275
C	2.00993	4.32765	8.37031
H	1.76671	3.38584	8.85903
H	1.37708	5.09669	8.81001
H	3.04014	4.57093	8.64451
C	2.60165	1.78471	6.67224
H	1.96505	1.48714	7.50789
H	3.62883	1.80178	7.04587
H	2.53901	0.98942	5.93187

TS2 (Sc)

Sc	-0.52985	0.18022	-4.57118
C	-0.24063	-0.95679	-2.66711
P	-0.03923	-1.74859	-1.34114
C	-2.51920	1.57082	-4.25106
C	-2.80829	0.31624	-3.66686
C	-2.81806	-0.65232	-4.69864
C	-2.56631	0.01038	-5.92575
C	-2.39862	1.39020	-5.64759
C	-2.47274	2.88352	-3.53440
H	-3.46664	3.33369	-3.46861
H	-1.83085	3.60510	-4.04107
H	-2.10307	2.78091	-2.51435
C	-3.19453	0.10036	-2.24349
H	-2.55240	0.64154	-1.55022
H	-3.14415	-0.95022	-1.96741
H	-4.22061	0.43721	-2.07476
C	-3.09861	-2.11103	-4.51916
H	-2.59505	-2.51392	-3.64015
H	-2.76913	-2.69669	-5.37749
H	-4.16862	-2.29580	-4.39981
C	-2.63142	-0.61685	-7.28278
H	-2.25560	-1.64021	-7.28552
H	-2.05440	-0.06304	-8.02226
H	-3.66110	-0.65551	-7.64621
C	-2.36262	2.50797	-6.63657
H	-2.14242	2.16479	-7.64424
H	-1.63027	3.27481	-6.38165
H	-3.33618	3.00274	-6.67378
C	1.85825	0.65438	-4.39672
C	1.74684	-0.61945	-5.00129
C	1.13010	-0.45441	-6.26390
C	0.89721	0.92709	-6.46184
C	1.31693	1.60991	-5.29353
C	2.50630	0.96076	-3.08674
H	3.58115	1.11323	-3.21139
H	2.36401	0.16081	-2.36350
H	2.11342	1.86754	-2.62734
C	2.28129	-1.91133	-4.47786
H	3.21375	-2.17435	-4.98351
H	1.58618	-2.73883	-4.62433
H	2.48701	-1.85286	-3.41268
C	0.92840	-1.53969	-7.27348
H	0.16784	-1.28280	-8.01015
H	0.62812	-2.48198	-6.81206
H	1.85156	-1.74034	-7.82334
C	0.55781	1.55287	-7.77407
H	0.21671	2.58052	-7.67344
H	-0.20377	1.00379	-8.32727
H	1.44879	1.57528	-8.40671
C	1.31961	3.09278	-5.08448
H	0.57683	3.59655	-5.70294
H	2.29065	3.52459	-5.33842
H	1.11836	3.36850	-4.04840
P	0.37850	0.37441	2.32328
C	0.36378	-1.11249	1.85133
Sc	0.34264	-3.24984	1.17291
C	-1.93611	-3.98243	0.43534
C	-2.10991	-2.94330	1.37743
C	-1.63604	-3.40906	2.62782
C	-1.18540	-4.73965	2.45756
C	-1.37078	-5.09211	1.09894
C	-2.46896	-4.02720	-0.96060
H	-3.34974	-4.67401	-1.00997
H	-1.74491	-4.41725	-1.67813
H	-2.77294	-3.04314	-1.31267
C	-2.80678	-1.65083	1.12149
H	-2.45583	-1.15315	0.21750
H	-2.65339	-0.95756	1.94228
H	-3.88411	-1.80398	1.01643
C	-1.69225	-2.67833	3.93119
H	-1.76174	-1.60238	3.78546
H	-0.80323	-2.85125	4.54012
H	-2.55541	-2.99593	4.52166
C	-0.84373	-5.67049	3.57449
H	-0.35582	-5.16165	4.40448
H	-0.19167	-6.48479	3.26288
H	-1.75225	-6.12866	3.97467

C	-1.29370	-6.45519	0.49154
H	-0.78627	-7.17125	1.13326
H	-0.78400	-6.45826	-0.47329
H	-2.30015	-6.84564	0.31613
C	2.69884	-2.96306	0.44194
C	2.72802	-3.00474	1.85101
C	2.31593	-4.28880	2.26803
C	2.05699	-5.05875	1.10883
C	2.26568	-4.23178	-0.02134
C	3.19667	-1.81332	-0.37048
H	4.27841	-1.70572	-0.25663
H	2.74291	-0.86597	-0.07299
H	3.00302	-1.95371	-1.43184
C	3.28841	-1.95194	2.74230
H	4.34966	-2.13880	2.93182
H	2.78572	-1.92444	3.70852
H	3.19677	-0.96174	2.30397
C	2.38605	-4.76827	3.68280
H	1.98188	-5.77119	3.80272
H	1.84577	-4.11592	4.37195
H	3.42283	-4.79808	4.02710
C	1.90596	-6.54326	1.06548
H	1.41157	-6.88731	0.15977
H	1.34924	-6.93950	1.91279
H	2.89228	-7.01455	1.08669
C	2.18377	-4.68098	-1.44654
H	1.39509	-5.41950	-1.59990
H	3.12001	-5.14340	-1.77016
H	1.97984	-3.85558	-2.12830
P	2.49318	3.18297	0.10508
C	1.63236	3.23747	1.40588
Sc	0.40759	3.31501	3.25665
C	-2.02478	2.91086	2.94524
C	-1.51202	3.29335	1.68265
C	-1.07550	4.63619	1.78567
C	-1.32677	5.08312	3.10320
C	-1.92439	4.01607	3.81705
C	-2.71952	1.63779	3.30246
H	-3.79194	1.80366	3.43835
H	-2.34591	1.19531	4.22900
H	-2.60493	0.89022	2.52322
C	-1.55309	2.49808	0.42011
H	-1.74833	1.44207	0.60431
H	-0.61399	2.55365	-0.13214
H	-2.35069	2.86711	-0.23100
C	-0.57824	5.48812	0.66405
H	-0.16967	4.88721	-0.14562
H	0.21432	6.16808	0.97856
H	-1.38843	6.09954	0.25632
C	-1.18914	6.49750	3.56487
H	-0.29414	6.97695	3.16909
H	-1.14454	6.57629	4.65031
H	-2.04313	7.09551	3.23614
C	-2.63591	4.08924	5.12817
H	-2.38538	4.98643	5.68806
H	-2.43595	3.23073	5.77141
H	-3.71664	4.11020	4.96133
C	2.31483	2.41537	4.55951
C	2.70726	3.70840	4.15418
C	1.85202	4.64179	4.78109
C	0.95433	3.92699	5.61004
C	1.21551	2.54499	5.44503
C	3.06304	1.16658	4.23596
H	3.96541	1.09338	4.84909
H	3.37460	1.14033	3.19158
H	2.47303	0.27124	4.42075
C	3.93427	4.04990	3.37720
H	4.75977	4.27445	4.05897
H	3.78821	4.91727	2.73515
H	4.24631	3.23157	2.73272
C	2.02956	6.12563	4.71994
H	1.17749	6.66078	5.13440
H	2.16963	6.48186	3.69759
H	2.91122	6.43734	5.28621
C	0.12320	4.53655	6.68876
H	-0.67350	3.88014	7.03174
H	-0.33415	5.47911	6.39194
H	0.75181	4.75095	7.55729
C	0.53318	1.42238	6.16387
H	-0.48191	1.68739	6.46252
H	1.07074	1.14179	7.07330
H	0.45988	0.52611	5.54527

Int2 (Sc)

Sc	-0.90394	0.26098	-4.31164
C	-0.44152	-0.90048	-2.45544
P	-0.11957	-1.70911	-1.16311
C	-2.94281	1.51200	-3.78495
C	-3.14477	0.20292	-3.29171
C	-3.15510	-0.68166	-4.39595
C	-3.00511	0.08616	-5.57667
C	-2.88397	1.44663	-5.19662
C	-2.91610	2.76882	-2.97333
H	-3.90501	3.23242	-2.92769
H	-2.23754	3.51362	-3.39265
H	-2.60053	2.58780	-1.94566
C	-3.47855	-0.15079	-1.88344
H	-2.86820	0.38655	-1.16135
H	-3.33773	-1.21200	-1.69670
H	-4.52509	0.08460	-1.67386

C	-3.35472	-2.16341	-4.32196
H	-2.80680	-2.60573	-3.48872
H	-3.02216	-2.66472	-5.23134
H	-4.40971	-2.41542	-4.19055
C	-3.14497	-0.43758	-6.97022
H	-2.70508	-1.42740	-7.09232
H	-2.67862	0.21451	-7.70688
H	-4.19908	-0.52395	-7.24471
C	-2.94136	2.63982	-6.09271
H	-2.79221	2.38025	-7.13799
H	-2.20562	3.40268	-5.83456
H	-3.92322	3.11433	-6.02041
C	1.49902	0.77077	-4.31487
C	1.34332	-0.48640	-4.94204
C	0.62211	-0.29323	-6.14393
C	0.35624	1.09049	-6.27863
C	0.86875	1.74444	-5.13047
C	2.28815	1.04327	-3.07504
H	3.35952	0.99992	-3.28401
H	2.07758	0.32963	-2.28042
H	2.08418	2.03407	-2.67446
C	1.93632	-1.78224	-4.49824
H	2.81316	-2.03240	-5.10121
H	1.23623	-2.61437	-4.58304
H	2.25354	-1.73593	-3.45981
C	0.35811	-1.35698	-7.16182
H	-0.40370	-1.06092	-7.88141
H	0.03094	-2.29402	-6.70713
H	1.26325	-1.58553	-7.73027
C	-0.10911	1.76016	-7.52979
H	-0.48376	2.76587	-7.35312
H	-0.88972	1.20472	-8.04777
H	0.72584	1.85403	-8.22904
C	0.87178	3.22180	-4.88283
H	0.06768	3.72897	-5.41571
H	1.80829	3.67671	-5.21415
H	0.76206	3.46756	-3.82552
P	0.61749	0.40624	2.28401
C	0.56469	-1.09373	1.87103
Sc	0.48922	-3.23820	1.28061
C	-1.84646	-3.97386	0.80069
C	-1.92637	-2.94610	1.76955
C	-1.31043	-3.42215	2.95196
C	-0.87463	-4.74744	2.72018
C	-1.20075	-5.08615	1.38524
C	-2.51836	-4.00800	-0.53427
H	-3.43005	-4.61082	-0.48902
H	-1.88865	-4.43917	-1.31447
H	-2.80721	-3.01440	-0.86939
C	-2.66294	-1.65746	1.62428
H	-2.61803	-1.26863	0.60761
H	-2.25144	-0.88832	2.27377
H	-3.71830	-1.78490	1.87951
C	-1.21651	-2.69640	4.25581
H	-1.28444	-1.61927	4.11806
H	-0.27315	-2.89172	4.76871
H	-2.01895	-2.99689	4.93457
C	-0.40836	-5.68282	3.78773
H	0.18992	-5.18110	4.54636
H	0.18592	-6.50755	3.39803
H	-1.26638	-6.12494	4.30089
C	-1.15933	-6.43980	0.75172
H	-0.56166	-7.14970	1.31816
H	-0.76159	-6.41767	-0.26438
H	-2.16838	-6.85557	0.68369
C	2.77635	-2.84419	0.37034
C	2.92319	-2.89816	1.77292
C	2.58542	-4.19824	2.20521
C	2.24989	-4.96482	1.06194
C	2.34551	-4.12141	-0.07143
C	3.17168	-1.66929	-0.46253
H	4.25331	-1.51944	-0.42087
H	2.70512	-0.74275	-0.12209
H	2.90936	-1.80605	-1.50984
C	3.52427	-1.83858	2.63262
H	4.59282	-2.02804	2.77182
H	3.06961	-1.80762	3.62204
H	3.41863	-0.84553	2.19996
C	2.79984	-4.69559	3.59855
H	2.39949	-5.69592	3.74905
H	2.34550	-4.04489	4.34846
H	3.86747	-4.74019	3.82813
C	2.12477	-6.45204	1.01603
H	1.57442	-6.79828	0.14304
H	1.63411	-6.86272	1.89677
H	3.11773	-6.90674	0.96423
C	2.15336	-4.56080	-1.48951
H	1.32569	-5.26492	-1.59486
H	3.04701	-5.05930	-1.87380
H	1.93987	-3.72357	-2.15319
P	3.93836	2.42145	1.17724
C	2.60911	2.71589	1.94322
Sc	0.71355	3.13576	3.03552
C	-1.18842	3.09197	1.40231
C	-0.05314	3.55156	0.69797
C	0.31925	4.80217	1.24993
C	-0.59815	5.11987	2.27849
C	-1.52286	4.05122	2.38276
C	-2.04691	1.92173	1.05192
H	-2.92604	2.25061	0.48800

H	-2.41656	1.39531	1.93235
H	-1.51927	1.18893	0.44244
C	0.53432	2.90834	-0.51285
H	0.49551	1.81927	-0.46632
H	1.57910	3.18126	-0.64421
H	-0.00920	3.21744	-1.41310
C	1.43307	5.67607	0.77060
H	2.26986	5.08715	0.39798
H	1.82296	6.31063	1.56725
H	1.10078	6.33761	-0.03390
C	-0.72258	6.45416	2.93957
H	0.23691	6.95494	3.05441
H	-1.17715	6.39300	3.92692
H	-1.35444	7.11449	2.33968
C	-2.79690	4.01844	3.16481
H	-2.85481	4.81380	3.90471
H	-2.94282	3.07274	3.69021
H	-3.65535	4.14171	2.49854
C	1.81920	2.07689	5.00502
C	2.38096	3.36564	4.87859
C	1.36444	4.31616	5.11761
C	0.16853	3.61468	5.41030
C	0.44411	2.22979	5.31868
C	2.60030	0.80545	4.95700
H	3.22747	0.70813	5.84704
H	3.25446	0.76581	4.08590
H	1.95619	-0.07194	4.91783
C	3.82705	3.69058	4.70110
H	4.28181	3.94363	5.66335
H	3.97843	4.53715	4.03188
H	4.38055	2.85538	4.27824
C	1.61526	5.78358	5.25631
H	0.69421	6.35473	5.34545
H	2.17369	6.19113	4.41130
H	2.20770	5.98874	6.15173
C	-1.07248	4.19877	6.00001
H	-1.95840	3.60699	5.77832
H	-1.26298	5.21451	5.65814
H	-0.98392	4.24369	7.08893
C	-0.52396	1.13560	5.64444
H	-1.52654	1.34104	5.26394
H	-0.61558	0.99729	6.72502
H	-0.21467	0.17978	5.22259

TS3 (Sc)

Sc	-0.01136	0.21696	-3.80242
C	-0.03284	-0.95099	-1.89648
P	-0.01138	-1.96514	-0.69120
C	-2.20272	1.39385	-3.95619
C	-2.46518	0.09657	-3.46010
C	-2.13639	-0.83166	-4.47731
C	-1.70610	-0.10566	-5.61182
C	-1.72729	1.27206	-5.28097
C	-2.56230	2.69132	-3.30666
H	-3.49150	3.08670	-3.72701
H	-1.79659	3.45601	-3.44569
H	-2.71345	2.58102	-2.23468
C	-3.15372	-0.21801	-2.17777
H	-2.80503	0.40481	-1.35624
H	-2.99361	-1.25157	-1.88072
H	-4.23252	-0.06736	-2.27259
C	-2.30328	-2.31551	-4.38994
H	-2.04196	-2.69125	-3.40057
H	-1.67082	-2.83987	-5.10715
H	-3.33419	-2.61564	-4.59459
C	-1.53832	-0.67973	-6.97984
H	-1.17234	-1.70417	-6.95745
H	-0.85601	-0.10238	-7.60041
H	-2.50082	-0.69729	-7.49799
C	-1.54486	2.43153	-6.20782
H	-1.12897	2.13182	-7.16657
H	-0.88981	3.20103	-5.79429
H	-2.50484	2.91295	-6.41237
C	2.44129	0.33050	-3.42489
C	2.18622	-0.90729	-4.05457
C	1.72257	-0.65168	-5.36278
C	1.69273	0.75280	-5.54740
C	2.10980	1.35995	-4.34060
C	3.11575	0.49138	-2.10498
H	4.16692	0.19936	-2.17239
H	2.65149	-0.11908	-1.33288
H	3.09218	1.52318	-1.76193
C	2.50912	-2.26157	-3.51801
H	3.42132	-2.65279	-3.97731
H	1.71424	-2.98468	-3.70586
H	2.66630	-2.23398	-2.44268
C	1.56271	-1.70851	-6.40764
H	1.11955	-1.32499	-7.32342
H	0.94576	-2.54384	-6.06961
H	2.53611	-2.12770	-6.67637
C	1.55771	1.46805	-6.85052
H	1.20001	2.48906	-6.73090
H	0.88814	0.96562	-7.54594
H	2.53282	1.52992	-7.34141
C	2.28786	2.83144	-4.13493
H	1.49436	3.41085	-4.61011
H	3.23369	3.17996	-4.55732
H	2.28313	3.10324	-3.07986
P	0.01521	0.41886	2.02349
C	-0.03625	-1.13489	1.77205

Sc	-0.01751	-3.36817	1.70820
C	-2.19995	-4.10032	0.74468
C	-2.47039	-3.03159	1.62893
C	-2.14439	-3.45959	2.93868
C	-1.70534	-4.80120	2.86634
C	-1.71694	-5.18930	1.50356
C	-2.55527	-4.18042	-0.70487
H	-3.45158	-4.79129	-0.84415
H	-1.76459	-4.62772	-1.30948
H	-2.76400	-3.19921	-1.12620
C	-3.15134	-1.75439	1.28101
H	-2.78628	-1.32630	0.34934
H	-2.99856	-1.00352	2.05217
H	-4.22943	-1.90531	1.17804
C	-2.32062	-2.65396	4.18680
H	-2.02934	-1.61358	4.04162
H	-1.71742	-3.04169	5.00838
H	-3.36016	-2.65981	4.52432
C	-1.54117	-5.71817	4.03402
H	-1.22714	-5.19475	4.93501
H	-0.82036	-6.51177	3.84878
H	-2.49413	-6.20189	4.26393
C	-1.51378	-6.56269	0.94993
H	-1.08674	-7.24256	1.68286
H	-0.86139	-6.57026	0.07416
H	-2.46754	-6.99421	0.63435
C	2.43718	-3.08813	1.45063
C	2.16696	-3.01743	2.83480
C	1.70308	-4.28050	3.26084
C	1.69707	-5.14398	2.13765
C	2.11936	-4.39829	1.01302
C	3.10417	-2.02163	0.65020
H	4.13895	-1.88537	0.97560
H	2.59979	-1.06166	0.73983
H	3.13692	-2.27619	-0.40705
C	2.46476	-1.87398	3.74663
H	3.26970	-2.13012	4.44142
H	1.60172	-1.57272	4.34154
H	2.78631	-0.99902	3.18681
C	1.51427	-4.65009	4.69707
H	1.13715	-5.66278	4.82011
H	0.82862	-3.97886	5.21798
H	2.46628	-4.59980	5.23271
C	1.58204	-6.63227	2.17407
H	1.26946	-7.05304	1.22001
H	0.88993	-6.99107	2.93311
H	2.55642	-7.07117	2.40590
C	2.31293	-4.94505	-0.36718
H	1.58888	-5.72764	-0.59850
H	3.30698	-5.38236	-0.49023

H	2.19766	-4.17645	-1.13077
P	0.00598	1.60610	-1.41287
C	-0.01163	2.17178	0.05750
Sc	0.01066	3.16841	2.06436
C	-2.16798	2.67300	3.17777
C	-2.44328	2.90862	1.81092
C	-2.12441	4.25974	1.53349
C	-1.68855	4.86537	2.73532
C	-1.69635	3.87525	3.74849
C	-2.51586	1.45185	3.96667
H	-3.44201	1.60915	4.52688
H	-1.74305	1.18848	4.69016
H	-2.66751	0.58394	3.32900
C	-3.11254	1.95103	0.88513
H	-2.65169	0.96472	0.90080
H	-3.07358	2.30280	-0.14298
H	-4.16664	1.82966	1.14828
C	-2.28920	4.94922	0.21551
H	-2.12031	4.26721	-0.61712
H	-1.58264	5.77143	0.09742
H	-3.29173	5.36996	0.10417
C	-1.52380	6.33417	2.94683
H	-1.20565	6.85159	2.04354
H	-0.80536	6.56919	3.72919
H	-2.47720	6.77796	3.24543
C	-1.49938	4.07711	5.21601
H	-1.08710	5.05679	5.44565
H	-0.83628	3.33126	5.65911
H	-2.45310	4.00146	5.74547
C	2.46245	2.79949	1.98408
C	2.20858	3.97765	1.24857
C	1.74330	4.96506	2.14301
C	1.71287	4.39925	3.44112
C	2.12947	3.05174	3.33824
C	3.12697	1.58168	1.43796
H	4.18176	1.77802	1.22777
H	2.66036	1.25007	0.51264
H	3.08956	0.74952	2.13774
C	2.53054	4.21748	-0.18802
H	3.48230	4.74670	-0.28865
H	1.76857	4.81664	-0.68761
H	2.60910	3.28286	-0.73736
C	1.57908	6.40545	1.77446
H	1.17623	7.00111	2.59026
H	0.92328	6.54570	0.91325
H	2.54483	6.84344	1.50904
C	1.58742	5.15315	4.72384
H	1.23022	4.53394	5.54437
H	0.92339	6.01298	4.65318
H	2.56674	5.53752	5.02174

C	2.30378	2.10602	4.48599
H	1.59428	2.30724	5.28977
H	3.30532	2.18080	4.91734
H	2.15600	1.06899	4.18609

$\{Sc(Cp^*)_2\}_3(\mu_3\text{-}C_3P_3)$ TS_{rot}

Sc	0.00736	-1.20238	-0.15497
C	-0.01471	-0.58257	2.00217
P	-0.03376	-1.84190	3.19002
C	-2.23384	-0.27585	-0.65681
C	-2.42824	-1.31194	0.28214
C	-2.04916	-2.53008	-0.33154
C	-1.67447	-2.25017	-1.66916
C	-1.77981	-0.85216	-1.86542
C	-2.58212	1.16303	-0.45836
H	-3.64953	1.33486	-0.62006
H	-2.04355	1.81276	-1.14874
H	-2.34380	1.50269	0.54963
C	-3.10847	-1.16822	1.59869
H	-2.82835	-0.24944	2.11012
H	-2.86682	-1.99169	2.26823
H	-4.19393	-1.16322	1.46562
C	-2.12308	-3.88165	0.31103
H	-1.76923	-3.85990	1.34363
H	-1.52023	-4.61917	-0.21965
H	-3.14793	-4.26091	0.32790
C	-1.46086	-3.27250	-2.73626
H	-0.99945	-4.18304	-2.35848
H	-0.83479	-2.90920	-3.54981
H	-2.41782	-3.56171	-3.17848
C	-1.67663	-0.11174	-3.16064
H	-1.25500	-0.72366	-3.95465
H	-1.06419	0.78906	-3.08861
H	-2.66669	0.20752	-3.49490
C	2.43218	-0.81375	0.17045
C	2.27923	-2.20441	-0.01828
C	1.83781	-2.42530	-1.34254
C	1.70663	-1.16477	-1.97463
C	2.04925	-0.16696	-1.02886
C	3.08152	-0.17321	1.34711
H	4.17021	-0.21907	1.24983
H	2.80780	-0.66188	2.28044
H	2.80712	0.87593	1.44284
C	2.64017	-3.26056	0.97507
H	3.70370	-3.21940	1.22178
H	2.43586	-4.26011	0.59201
H	2.08735	-3.15253	1.91097
C	1.75641	-3.76665	-2.00065
H	1.32099	-3.70558	-2.99510
H	1.16421	-4.48581	-1.43222
H	2.75367	-4.19865	-2.11483
C	1.51032	-0.93901	-3.43732
H	1.08939	0.04025	-3.65717
H	0.85947	-1.68121	-3.89681
H	2.46995	-0.99382	-3.95828
C	2.09381	1.31108	-1.26619
H	1.46438	1.61046	-2.10500
H	3.10757	1.65078	-1.49206
H	1.75241	1.87051	-0.39340
P	-0.02411	0.40072	5.32712
C	-0.02968	-1.25242	4.81741
Sc	-0.01974	-2.82222	6.41833
C	-2.24768	-3.71648	5.82267
C	-2.45559	-2.39627	6.27776
C	-2.08792	-2.34744	7.64516
C	-1.71206	-3.65209	8.04791
C	-1.79542	-4.49424	6.91374
C	-2.55211	-4.26060	4.46445
H	-3.45038	-4.88390	4.48232
H	-1.74204	-4.87599	4.06825
H	-2.72262	-3.46606	3.74163
C	-3.12010	-1.29496	5.52759
H	-2.92127	-1.34185	4.45885
H	-2.77835	-0.31820	5.86569
H	-4.20337	-1.33483	5.67060
C	-2.18173	-1.13374	8.51728
H	-1.83014	-0.23643	8.00391
H	-1.58863	-1.23671	9.42680
H	-3.21179	-0.94294	8.82901
C	-1.51787	-4.09999	9.45936
H	-1.09731	-3.32102	10.09287
H	-0.86869	-4.97027	9.53773
H	-2.47847	-4.38068	9.89902
C	-1.67773	-5.98421	6.87906
H	-1.21119	-6.38399	7.77577
H	-1.10156	-6.34113	6.02299
H	-2.66809	-6.44038	6.80060
C	2.41204	-2.71281	5.95363
C	2.22656	-2.19658	7.25505
C	1.78150	-3.24683	8.09041
C	1.67903	-4.41734	7.30111
C	2.03844	-4.07816	5.97337
C	3.07868	-1.99911	4.82907
H	4.16421	-2.01944	4.96142
H	2.77048	-0.95744	4.75831
H	2.86238	-2.46164	3.86804
C	2.56677	-0.81413	7.70597
H	3.64158	-0.70661	7.87520
H	2.06631	-0.55507	8.63931
H	2.27409	-0.06626	6.96982

C	1.67671	-3.15392	9.57966
H	1.22394	-4.04062	10.01621
H	1.09121	-2.29402	9.91085
H	2.66898	-3.04748	10.02484
C	1.49888	-5.80733	7.81684
H	1.07799	-6.48014	7.07209
H	0.85852	-5.85076	8.69620
H	2.46576	-6.22514	8.10925
C	2.10180	-5.01755	4.80885
H	1.45224	-5.88260	4.94924
H	3.11333	-5.40243	4.65644
H	1.79538	-4.53458	3.87955
P	-0.02665	1.12429	2.31448
C	-0.02843	1.51270	4.00147
Sc	-0.00877	3.68540	4.55674
C	-2.24483	3.60376	5.62354
C	-2.44703	3.36380	4.24740
C	-2.06965	4.53523	3.54450
C	-1.69335	5.51672	4.49444
C	-1.78800	4.93280	5.77915
C	-2.57633	2.68658	6.75608
H	-3.44404	3.05361	7.31096
H	-1.75802	2.58143	7.47163
H	-2.81621	1.68636	6.40436
C	-3.11710	2.17636	3.64698
H	-2.85141	1.24817	4.14859
H	-2.84983	2.05215	2.59897
H	-4.20366	2.28846	3.69521
C	-2.14809	4.71365	2.05943
H	-1.81131	3.82316	1.52494
H	-1.53231	5.54531	1.71570
H	-3.17072	4.91855	1.73317
C	-1.48660	6.96774	4.20906
H	-1.07958	7.14848	3.21573
H	-0.81945	7.44776	4.92333
H	-2.44007	7.50031	4.26007
C	-1.67990	5.62298	7.10077
H	-1.23569	6.61210	7.01715
H	-1.08923	5.05793	7.82430
H	-2.67140	5.75428	7.54238
C	2.42195	3.21436	4.66532
C	2.23730	4.11046	3.59024
C	1.79939	5.34827	4.11516
C	1.70238	5.21412	5.52213
C	2.06175	3.88558	5.85797
C	3.08944	1.88658	4.56789
H	4.17454	2.01375	4.51090
H	2.77157	1.32935	3.68926
H	2.88303	1.26160	5.43492
C	2.56372	3.82795	2.15861
H	3.60512	3.51683	2.04747
H	2.42361	4.70922	1.53323
H	1.94463	3.03048	1.74044
C	1.69335	6.61455	3.32508
H	1.29599	7.43520	3.91797
H	1.05602	6.51752	2.44434
H	2.67851	6.92646	2.97003
C	1.52056	6.33080	6.49735
H	1.08232	5.99868	7.43633
H	0.88914	7.13005	6.11239
H	2.48726	6.78011	6.73931
C	2.13785	3.30160	7.23566
H	1.57075	3.88801	7.95921
H	3.16831	3.26029	7.59731
H	1.74372	2.28383	7.26886

4. References

[1] D. W. N. Wilson, S. J. Urwin, E. S. Yang, J. M. Goicoechea, *J. Am. Chem. Soc.* **2021**, *143*, 10367–10373.

[2] D. J. Berg, C. J. Burns, R. A. Andersen, A. Zalkin, *Organometallics* **1989**, *8*, 1865–1870.

[3] R. A. Kelly, N. M. Scott, S. Díez-González, E. D. Stevens, S. P. Nolan, *Organometallics* **2005**, *24*, 3442–3447.

[4] G. S. Girolami, A. P. Satterberger, **n.d.**, 333.

[5] CrysAlisPro, Agilent Technologies, Version 1.171.35.8.

[6] (a) G. M. Sheldrick in SHELXL97, Programs for Crystal Structure Analysis (Release

97-2), Institut für Anorganische Chemie der Universität, Tammanstrasse 4, D-3400 Göttingen, Germany, 1998; (b) G. M. Sheldrick, *Acta Crystallogr. Sect. A* **1990**, *46*, 467−473; (c) G. M. Sheldrick, *Acta Crystallogr. Sect. A* **2008**, *64*, 112−122.

[7] F. Neese, *Wiley Interdiscip. Rev. Comput. Mol. Sci.* **2012**, *2*, 73–78.

[8] F. Neese, *Wiley Interdiscip. Rev. Comput. Mol. Sci.* **2018**, *8*, 1–6.

[9] F. Neese, F. Wennmohs, U. Becker, C. Riplinger, *J. Chem. Phys.* **2020**, *152*, 224108.

[10] S. Grimme, *J. Comput. Chem.* **2006**, *27*, 1787–1799.

[11] F. Weigend, R. Ahlrichs, *Phys. Chem. Chem. Phys.* **2005**, *7*, 3297–3305.

[12] Y. S. Lin, G. De Li, S. P. Mao, J. Da Chai, *J. Chem. Theory Comput.* **2013**, *9*, 263–272.

[13] D. A. Pantazis, X. Y. Chen, C. R. Landis, F. Neese, *J. Chem. Theory Comput.* **2008**, *4*, 908–919.

[14] M. Bühl, C. Reimann, D. A. Pantazis, T. Bredow, F. Neese, *J. Chem. Theory Comput.* **2008**, *4*, 1449–1459.

[15] C. Adamo, V. Barone, *J. Chem. Phys.* **1999**, *110*, 6158–6170.

[16] E. D. Glendening, J. K. Badenhoop, A. E. Reed, J. E. Carpenter, J. A. Bohmann, C. M. Morales, P. Karafiloglou, C. R. Landis, F. Weinhold, **2018**.

[17] T. Lu, F. Chen, *J. Comput. Chem.* **2012**, *33*, 580–592.
